# Anti-SARS-CoV-2 Activity and Cytotoxicity of Amaryllidaceae Alkaloids from *Hymenocallis littoralis*

**DOI:** 10.3390/molecules28073222

**Published:** 2023-04-04

**Authors:** Ngoc-Thao-Hien Le, Steven De Jonghe, Kristien Erven, Tom Vermeyen, Aliou M. Baldé, Wouter A. Herrebout, Johan Neyts, Christophe Pannecouque, Luc Pieters, Emmy Tuenter

**Affiliations:** 1 Natural Products and Food Research and Analysis (NatuRA), Department of Pharmaceutical Sciences, University of Antwerp, Universiteitsplein 1, B-2610 Antwerp, Belgium; 2 Laboratory of Virology and Chemotherapy, Department of Microbiology, Immunology and Transplantation, Rega Institute for Medical Research, KU Leuven, Herestraat 49, B-3000 Leuven, Belgium; 3 Department of Chemistry, University of Antwerp, Groenenborgerlaan 171, B-2020 Antwerp, Belgium; 4 Ghent Quantum Chemistry Group, Department of Chemistry, Ghent University, Krijgslaan 281, B-9000 Ghent, Belgium; 5 Department of Pharmacy, University Gamal Abdel Nasser of Conakry, Conakry 00224, Guinea

**Keywords:** Amaryllidaceae alkaloids, *Hymenocallis littoralis*, lycorine-type, galanthamine-type, DFT calculation, SARS-CoV-2

## Abstract

The Amaryllidaceae species are well-known as a rich source of bioactive compounds in nature. Although *Hymenocallis littoralis* has been studied for decades, its polar components were rarely explored. The current phytochemical investigation of Amaryllidaceae alkaloids from *H. littoralis* led to the identification of three previously undescribed compounds: *O*-demethyl-norlycoramine (**1**), (−)-2-*epi*-pseudolycorine (**2**) and (+)-2-*epi*-pseudolycorine (**3**), together with eight known compounds: 6α-hydroxyhippeastidine (**4**), 6β-hydroxyhippeastidine (**5**), lycorine (**6**), 2-*epi*-lycorine (**7**), zephyranthine (**8**), ungeremine (**9**), pancratistatin (**10**) and 9-*O*-demethyl-7-*O*-methyllycorenine (**11**). Among the eight previously reported compounds, five were isolated from *H. littoralis* for the first time (compounds **4**, **5**, **7**, **8,** and **9**). Compounds **1**, **4**, **5**, **7**, **8**, and **11** exhibited weak anti-SARS-CoV-2 activity (EC_50_ = 40–77 µM) at non-cytotoxic concentrations. Assessment of cytotoxicity on the Vero-E6 cell line revealed lycorine and pancratistatin as cytotoxic substances with CC_50_ values of 1.2 µM and 0.13 µM, respectively. The preliminary structure-activity relationship for the lycorine-type alkaloids in this study was further investigated, and as a result ring C appears to play a crucial role in their anti-SARS-CoV-2 activity.

## 1. Introduction

Amaryllidaceae alkaloids (AAs) belong to the large group of isoquinoline alkaloids, and they are almost exclusively found in the Amaryllidaceae family [1,2]. These alkaloids show a wide variety of pharmacological activities, such as anticancer, antiplasmodial, anti-inflammatory, antioxidant, etc. One of them, galanthamine, is a worldwide registered drug for the treatment of cognitive decline in mild to moderate Alzheimer’s disease and various other memory impairments [3,4]. To date, over 600 Amaryllidaceae alkaloids have been reported up to the end of 2018, and the number is still increasing [5].

*Hymenocallis littoralis* (Jacq.) Salisb. or ‘spider lily’ (Amaryllidaceae) is widely distributed by the sea and in swamps in tropical, sub-tropical and temperate climates throughout the world [6]. Alongside its medicinal value, it is also known for its horticultural and ornamental appeal [7]. This plant species has been the subject of research for decades and up to now, to our knowledge, 37 alkaloids have been isolated from *H. littoralis*, including four lycorine-type alkaloids (lycorine, littoraline, diacetyllycorine, norpluviine), four lycorenine-type alkaloids (lycorenine, homolycorine, hippeastrine, 6-*O*-methyllycorenine), seven haemanthamine- and crinine-type alkaloids (haemanthamine, vittatine, 11-hydroxyvittatine, crinine, *O*-acetyldihydrocrinine, 8-*O*-demethylmaritidine, bowdensine), five pretazettine-type alkaloids (tazettine, pretazettine, macronine, tazettamide, hymenolitatine G), five phenanthridone-type alkaloids (pancratistatin, narciclasine, lycoricidine, 7-deoxy-*trans*-dihydronarciclasine, isocarbostyril), two phenanthridine-type alkaloids (trisphaeridine, 5,6-dihydrobicolorine), one galanthamine-type alkaloid (lycoramine), five plicamine-type (6-oxo-5,6-dihydroplicane; 5,6-dihydroplicane, hymenolitatines A-C), two secoplicamine-type (hymenolitatines D and E), one belladine-type (hymenolitatine F), and an unclassified alkaloid (hymenolitatine) [7,8,9,10,11,12,13]. The most recent study of *H. littoralis* was published by the authors concerning the application of DFT calculations for structure elucidation of an epimeric mixture of 6α-hydroxyhippeastidine and 6β-hydroxyhippeastidine [14].

A brief overview of some pharmacological studies on *H. littoralis* is summarized hereafter. Lin et al. (1995) first reported that the alkaloidal extract of *H. littoralis* showed in vitro cytotoxic activity and further investigations of the phytochemical constituents proved that two out of their 14 isolated compounds (lycorine and haemanthamine) exhibited cytotoxicity against 11 cultured cell lines [10]. Afterwards, several studies conducted in the period 2005–2016 showed that the phenanthridone-type alkaloids of this plant species, including narciclasine, lycoricidine, and especially pancratistatin, exhibited potent selective toxicity against human tumor cells, as well as antiparasitic activity [15,16,17]. In 2014, Ji et al. studied the anti-tumor activity of a mixture of three alkaloids isolated from *H. littoralis* (5,6-dihydrobicolorine, 7-deoxy-*trans*-dihydronarciclasine and littoraline), pointing out a possible mode of action for apoptosis induction through the Fas-signaling pathway [18]. In 2016, Chen et al. reported a moderate in vitro cytotoxic activity of hymenolitatine against four cell lines [12]. Lastly, antiproliferation was further assessed by Ma et al. revealing jonquailine, 6-*O*-methylpretazettine, and hymenolitatine F as moderate antiproliferative agents [13].

Natural products as antiviral substances have been a subject of study for decades, and in particular, weak-to-potent antiviral activities were reported for many Amaryllidaceae alkaloids against viruses in Arenaviridae, Retroviridae, Piconaviridae, Togaviridae, Flaviviridae, Phenuiviridae, Paramyxoviridae, Coronaviridae, Rhabdoviridae, Orthomyxoviridae, Filoviridae, Virgaviridae, Herpesviridae and Poxviridae families [19,20]. Since the severe acute respiratory syndrome-related coronavirus-2 (SARS-CoV-2) outbreak commenced in December 2019, medicinal plants as a complementary and/or alternative medicine to fight against SARS-CoV-2 have remained an intriguing research topic and a common practice at many places around the world, alongside the application of vaccines and synthetic drugs [21,22,23,24]. In the present study, three unreported Amaryllidaceae alkaloids were identified, including two lycorine-type and one galanthamine-type alkaloids, alongside eight known alkaloids from *H. littoralis*. The chemical structures of all compounds were elucidated based on extensive 1D- and 2D-NMR spectroscopy and HRMS (High Resolution Mass Spectrometry) data. Propositions of relative configurations were based on *J*-coupling and nuclear Overhauser effects (NOE). Comparison between experimental and calculated optical rotation has benefited the determination of absolute configuration recently [25], and this technique was also applied in this study. At last, the anti-SARS-CoV-2 activity and cytotoxicity were assessed in vitro for all purified compounds to shed some light on the applicability of Amaryllidaceae alkaloids in SARS-CoV-2 treatment. Results obtained were also compared with recently published data of related compounds.

## 2. Results and Discussion

### 2.1. Structure Elucidation

**Figure 1:**
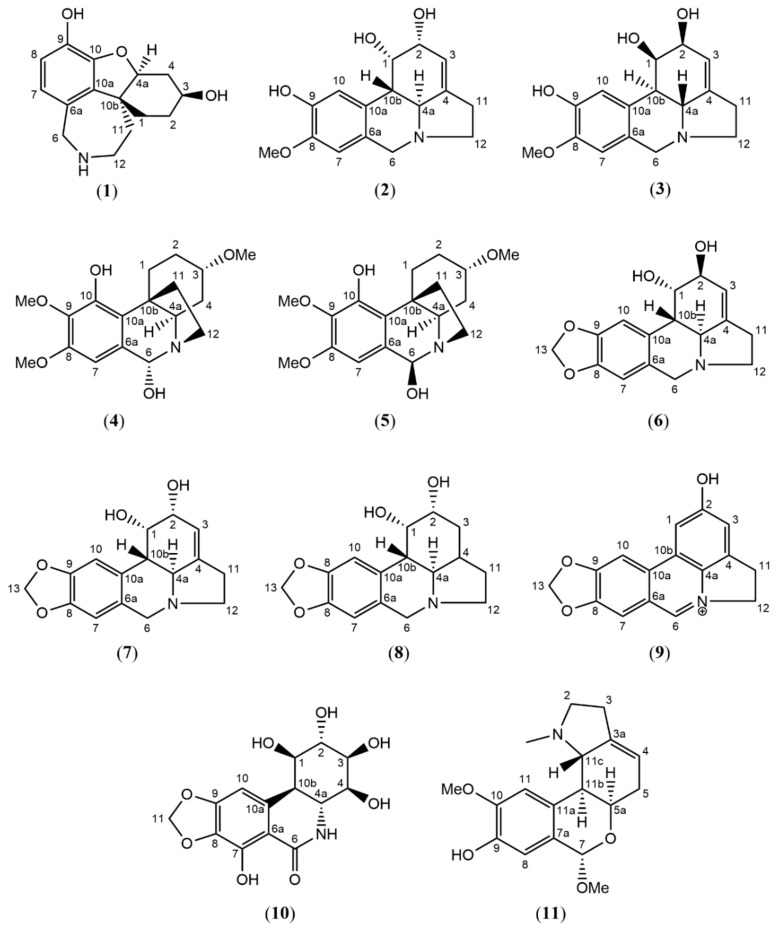
Structures of compounds **1**–**11** isolated from the bulbs of *Hymenocallis littoralis*.

The current phytochemical study of *H. littoralis* resulted in three unreported compounds, which were all isolated from the *n*-BuOH extract, alongside eight known compounds (see Figure 1).

One of the common ^1^H NMR spectroscopic patterns for Amaryllidaceae alkaloids is the presence of a methylenedioxy proton resonance around 5.95 ppm (-O-CH_2_-O- signal) [26,27]. In the absence of this characteristic signal, substituents such as hydroxy- or methoxy groups are prevalent instead of the methylenedioxy moiety. Using this NMR pattern as a starting point, five compounds (**6**–**10**) were found to possess the methylenedioxy moiety, and five compounds (**2**–**5**, **11**) were found to bear other substituents (Figure 1). The detailed structure elucidation procedure of the three unreported compounds (**1**–**3**) is described hereafter, and the NMR data of known compounds can be consulted in the Supporting Information.

**Figure 2:**
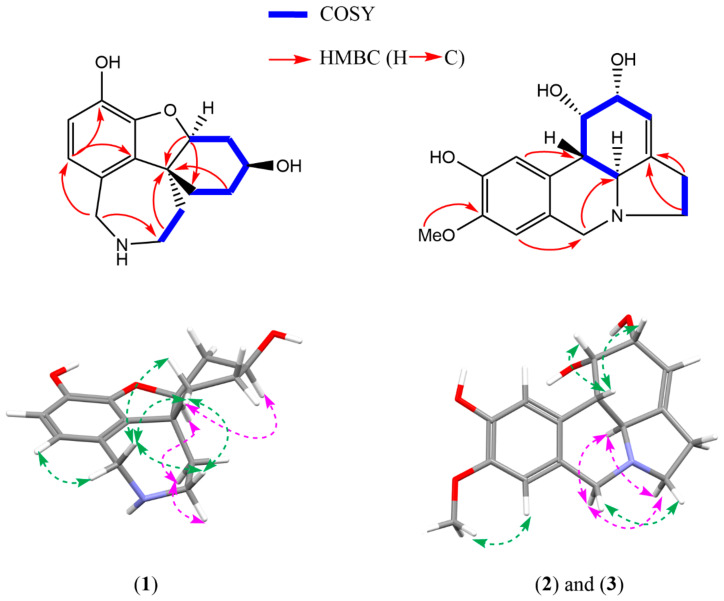
Key HMBC and COSY correlations for compounds **1**–**3** and selected NOESY correlations for compounds **1**–**3**. Green arrows: NOESY correlations above the plane (β-orientation); Pink arrows: NOESY correlations behind the plane (α-orientation).

**Figure 3:**
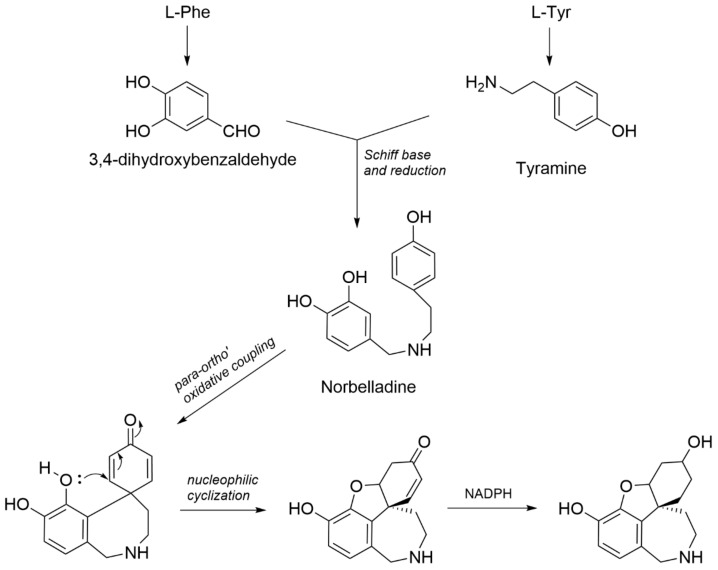
Proposed biosynthetic scheme of compound **1**.

Compound **1** was obtained as an off-white and amorphous solid. HRMS-based elemental analysis suggested a chemical formula of C_15_H_19_NO_3_ (*m*/*z* 262.1446 [M+H]^+^, calculated for C_15_H_20_NO_3_). Its ^1^H NMR spectrum showed two ortho-oriented aromatic hydrogens (δ_H_ 6.51 and δ_H_ 6.55, *J* = 8.0 Hz), which are typical for the galanthamine-type skeleton [27]. Indeed, the ^1^H and ^13^C NMR spectra were similar to those reported for galanthamine, but two clear differences could be observed: (1)—Compound **1** does not have two olefinic protons around 6 ppm like galanthamine, implying the absence of the double bond in the structure of galanthamine, and (2)—Compound **1** does not show any signals typical for -OCH_3_ or -NCH_3_ groups, indicating the absence of the methoxy and the N-methyl groups in the structure of galanthamine. Therefore, compound **1** was identified as *O*-demethyl-norlycoramine (or 1,2-dihydro-*O*-demethyl-*N*-demethylgalanthamine). Correlations of the 2D NMR spectra were in agreement with the proposed structure (Figure 2). Four methylene groups resonated in the upfield region (seven protons in the range of 1.4–2.0 ppm). In COSY, correlations between H-1 and H-2, H-2 and H-3, H-3 and H-4, and H-4 and H-4a confirmed that they are part of the same spin system (see Figure 2). Similarly, another spin system was observed for H-11 and H-12 on COSY. These COSY correlations were crucial to correctly assign the positions of the overlapping proton signals (see Table 1). Assignment of H-7 was confirmed by the NOE correlations between H-7 and H-6. Key HMBC correlations listed as follows aided in confirming the 2D structure (Figure 2): C-10b with H-2, H-4a, H-12; C-1 with H-4a; C-12 with H-6; C-7 with H-6; C-9 with H-7; C-10a with H-7. Finally, the molecular formula C_15_H_19_NO_3_ was confirmed by HRMS-based elemental analysis. Remarkably, a search based on the chemical structure of this compound gave an exact match on Scifinder (checked on 24 March 2023), but not a single reference reporting this compound could be found. Thus, to the best of our knowledge, compound **1** was reported for the first time as a natural product in this study. Biosynthetically, since the substituent of C-9 is a phenolic hydroxyl instead of a methoxy, compound **1** could originate directly from a para-ortho’ oxidative phenol coupling of norbelladine [5,28]. Enzymes responsible for methylation might not be involved in its biosynthetic pathway (Figure 3).

Owing to the stereocenter at C-10b, the 3D structure of compound **1** does not show a planar scaffold, from which two sides of a plane can easily be distinguished, like compounds **2** and **3** (see Figure 2). The NOESY spectrum of compound **1** showed strong cross-peaks between δ_H_-1.67, δ_H_-1.85, δ_H_-3.97 and δ_H_-3.19, indicating their spatial proximity. Next, the NOE effect observed between H-4a (δ_H_-4.31) and δ_H_-1.75 implied that they are spatially close to each other. Another important NOE effect was observed between δ_H_-1.67 and H-3 (δ_H_-4.09), indicating δ_H_-1.67, δ_H_-4.09, δ_H_-4.31 and δ_H_-1.75, and δ_H_-3.26 are co-facial (see Table 1 and Figure 2). Finally, the configuration of the 3-OH was assigned on the same side with δ_H_-1.67 due to a weak NOESY cross-peak between them. Combining all data together, the relative configuration of compound **1** was proposed as (3*S*,4a*S*,10b*R*)*-O*-demethyl-norlycoramine or its enantiomer. 

As a follow-up, DP4+ probability was assessed to compare the closeness between the experimental and calculated chemical shifts of all possible diastereomers. The computed diastereomers were chosen as follows: For compound **1**, the presence of three stereocenters infers eight possible configurations (four pairs of enantiomers). As the DP4+ method can only be used to assign a relative configuration, due to the incapacity of NMR to determine absolute configuration, the chemical shift values of only one member of every pair of enantiomers needed to be computed. As shown in Table 2, the highest probability was found for diastereomer (4a*S*,3*S*,10b*R*), which is in line with the configuration deduced from NOE correlations. Therefore, the relative configuration of compound **1** was confirmed, and the absolute configuration is either (4a*S*,3*S*,10b*R*) or (4a*R*,3*R*,10b*S*). It is important to state that the probability of 73% alone is not conclusive and validation by interatomic distances obtained through the NOESY experiment was required. This practice was recommended by the authors of a recent review on the topic of DP4+ probability [29]. As listed in Table 1, calculated chemical shifts of the diastereomer (4a*S*,3*S*,10b*R*) showed a strong resemblance with the experimental ones. In addition, this ascertained that the proposed planar structure was correct.

Next, specific optical rotations of the two configurations (4a*S*,3*S*,10b*R*) and (4a*R*,3*R*,10b*S*) were computed. Since a pair of enantiomers will have opposite optical activity, the experimental optical rotation was expected to match with only one of the two. According to Table 3, the configuration (4a*S*,3*S*,10b*R*) is levorotatory, and the configuration (4a*R*,3*R*,10b*S*) is dextrorotatory. Apart from the sign, there is a noticeable difference between the magnitudes of the experimental and the computed OR of the configuration (4a*S*,3*S*,10b*R*), which could be due to impurities in the sample (see ^1^H spectrum in Supplementary Materials). However, since the purity of the sample is approximately 90%, it can be assumed that compound **1** determines the overall sign of the sample. Therefore, the absolute configuration of compound **1** is defined as (4a*S*,3*S*,10b*R*).

Compounds **2** and **3** were obtained as a yellowish solid mixture. A chemical formula of C_16_H_19_NO_4_ was deduced from the *m/z* 290.1392 [M+H]^+^ (calculated for C_16_H_20_NO_4_). The NMR spectra were initially recorded in CD_3_OD and showed the general characteristics of lycorine-type alkaloids [27]. In the downfield region, two singlets corresponding to the para-oriented aromatic protons appeared at δ_H_ greater than 6.0 ppm (H-10/6.88 ppm and H-7/6.76 ppm), together with a broad singlet at δ_H_ 5–6 ppm (H-3/5.59 ppm), which represented the olefinic proton attached to C-3. Furthermore, similar to many lycorine-type alkaloids, compounds **2** and **3** also showed two doublets corresponding to an AB system of the benzylic methylene at C-6 (H-6α and H-6β, *J* = 14.1 Hz) [27]. Taking into consideration the fact that compounds **2** and **3** do not have a methylenedioxy moiety as discussed above, and the fact that a single methoxy-group (δ_H_ 3.84 and δ_C_ 56.5) is present, compounds **2** and **3** appear to have one hydroxy- and one methoxy-group in positions 8 and 9, instead of the methylenedioxy moiety. The position of the methoxy-group was deduced from a NOESY cross-peak between δ_H_ 3.84 and H-7 (δ_H_ 6.76) and an HMBC correlation between δ_H_ 3.84 and δ_C_ 147.8 (Figure 2). Another two key HMBC correlations were between H-7 and C-6 and between H-10 and C-10b. Furthermore, the COSY spectrum showed strong cross-peaks between three couples of protons: H-2 and H-1, H-1 and H-10b, and H-10b and H-4a (Figure 2). Taking these data together and by comparison with reported NMR data of lycorine-type alkaloids, it was found that the planar structures of compounds **2** and **3** were identical to the structure of pseudolycorine. This was confirmed by the molecular formula C_16_H_19_NO_4_ obtained by HRMS-based elemental analysis. However, several hydrogens of compounds **2** and **3** at the upfield region (2.50–4.00 ppm) were more deshielded than those of pseudolycorine (Figure S19), which could be indicative for a differing stereochemistry.

DP4+ probability was examined thereafter for compounds **2** and **3** in order to obtain proof for their relative configuration. Nonetheless, no decisive probability (90–100%) was observed. Although the SRRS configuration had the highest probability (61%), the dihedral angle between H-10b and H-4a of this configuration can only produce a J_H-10b/H-4a_ of 5.52 Hz (Table 2). Therefore, using the experimental J_H-10b/H-4a_ of 11.0 Hz as a constraint, the SRRS configuration was ruled out, and based on the calculated *J*-couplings, SRRR, SRSS, SSRR or SSSS could be the possible configuration (Table 2). Since SSSS is the known configuration of pseudolycorine, only three possibilities remained: SRRR, SRSS and SSRR. Although these three configurations possessed a DP4+ probability of 0% (Table 2), the use of J_H-10b/H-4a_ as a restriction in this case is of more reliable since it is well-known that a *trans*-conjunction between two fused rings of lycorine-type skeleton exhibits a typical *J*-coupling of 10–11 Hz.^27^ The DP4+ probability, however, only takes into account experimental and calculated chemical shifts as inputs, and the coupling constants are not incorporated in the stereochemistry determination by DP4+ [30]. In addition, the accuracy of DP4+ probability can be affected by many reasons and unsuccessful examples were also reported by the authors of this method [29]. 

Further analysis of NOE interactions also inferred that compounds **2** and **3** are diastereomers of pseudolycorine. As can be seen in Figure 2, spatial proximities were observed for H-1, H-2 and H-10b in the NOESY spectrum, indicating that these three hydrogens are on the same side of the plane. H-1 and H-2 appeared as two broad singlets with no noticeable splitting, suggesting a *cis*-orientation. In addition, H-10b and H-4a shared a *J*-coupling of 11.1 Hz, typical for the *trans*-orientation between these two hydrogens reported for the lycorine-type scaffold as mentioned above [27]. Therefore, combining NOE correlations and *J*-coupling information, it can be deduced that H-1, H-2 and H-10b were on the same side and were on the opposite side of H-4a. Among the three remaining possibilities as discussed above, the SRSS is the sole configuration that can show all these NOESY correlations. Hence, the relative configuration of compounds **2** and **3** was established as (1*S*,2*R*,4a*S*,10b*S*)-pseudolycorine, or its enantiomer. The structures of compounds **2** and **3** were elucidated with the configuration of one chiral center (at position H-2) differing from the configuration reported for pseudolycorine, and thus, correspond to 2-*epi*-pseudolycorine. To be noticed, C-4 was not observed in CD_3_OD, whereas it could be observed in (CD_3_)_2_SO in both HMBC and ^13^C-NMR spectra. Table 4 shows NMR data of compounds **2** and **3** in CD_3_OD and (CD_3_)_2_SO.

As reported in Table 3, the experimental optical rotation of 2-*epi*-pseudolycorine (compounds **2** and **3**) was only −8.0 and was significantly different from the computed values, ranging from 227.9 to 230.6, if dextrorotatory, and from −230.4 to −227.7, if levorotatory. Based on these data, it was not possible to deduce the absolute configuration of compounds **2** and **3**. At this point, it was hypothesized that the sample consisted of a racemic mixture. The fact that the measured value is not 0, but −8, could be caused by the presence of some impurities and/or the measurement or weighing errors due to the small amount of material. In order to determine whether the sample consisted of a racemate, an enantioseparation was performed, and the obtained HPLC chromatogram of the mixture of compounds **2** and **3** indeed showed two peaks with almost equal peak areas (Figure S20). Hence, the mixture of compounds **2** and **3** was confirmed to be a racemate. Compounds **2** and **3**, occurring as an enantiomeric mixture, were reported for the first time and the name 2-*epi*-pseudolycorine was adopted. Vibrational circular dichroism (VCD) spectrum of compounds **2** and **3** in (CD_3_)_2_SO was devoid of any signal, confirming the existence of a racemic mixture (Figure S23). Finally, the assignment of (−)-2-*epi*-pseudolycorine (**2**) to the configuration (1*S*, 2*R*, 4a*S*, 10b*S*) and (+)-2-*epi*-pseudolycorine (**3**) to the configuration (1*R*, 2*S*, 4a*R*, 10b*R*) was made according to their simulated optical rotations (see Table 3).

### 2.2. Anti-SARS-CoV-2 Activity and Cytotoxicity

**Figure 4:**
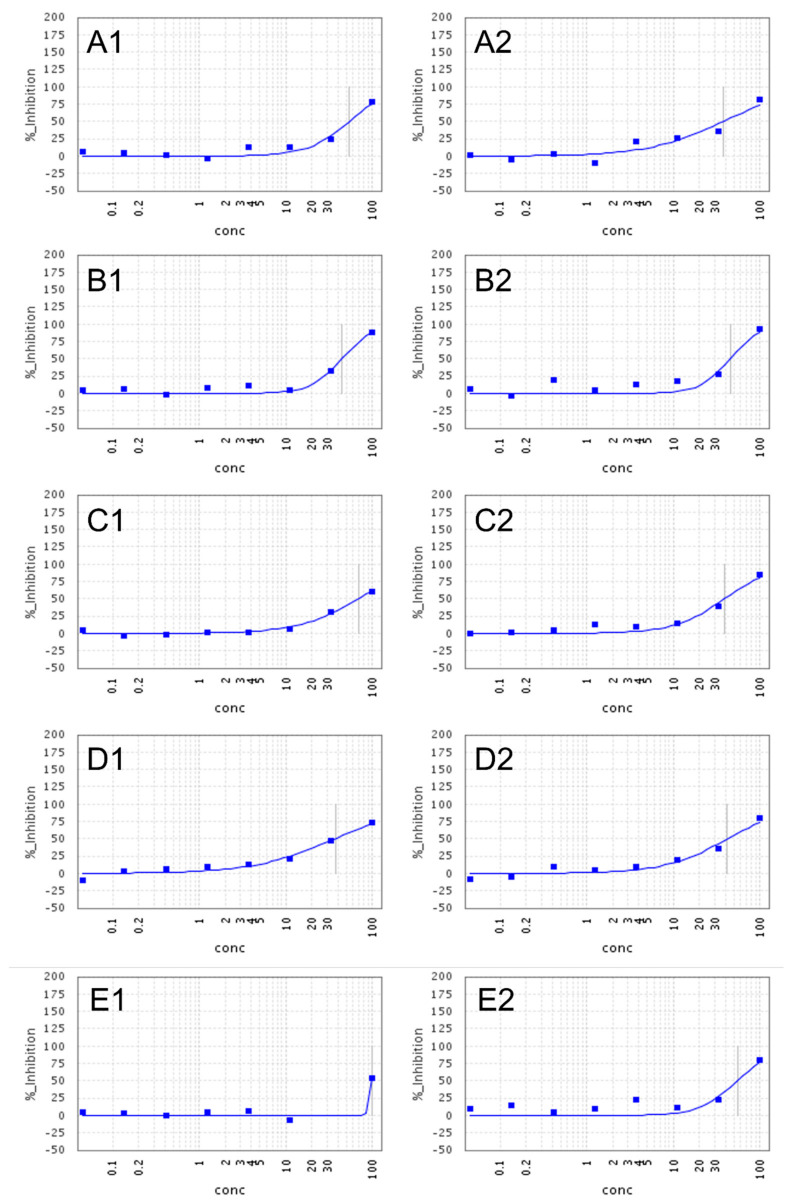
Dose-dependent inhibition SARS-CoV-2 replication of compounds **1** (**A**), **4** and **5** (**B**), **7** (**C**), **8** (**D**) and **11** (**E**). Each experiment was performed in duplicate; concentrations are in µM.

Compounds **1**, **4**, **5**, **7**, **8**, and **11** (*O*-demethyl-norlycoramine, 6α-hydroxyhippeastidine, 6β-hydroxyhippeastidine, 2-*epi*-lycorine, zephyranthine, and 9-*O*-demethyl-7-*O*-methyllycorenine, respectively) exhibited weak inhibition of SARS-CoV-2 (EC_50_ = 40–77 µM), at non-cytotoxic concentrations (CC_50_ > 100 µM) (see Figure 4). Compounds **1**, **4**, **5**, and **8** were the most potent ones among all tested compounds, displaying EC_50_ values of 45 µM, 44 µM, and 39 µM, respectively. Compounds **6** and **10** (lycorine and pancratistatin, respectively) lacked selective antiviral activity, since they were cytotoxic for the Vero-E6 cells as evidenced by CC_50_ values of 1.2 µM and 0.13 µM, respectively. Thus, our finding for lycorine is in contrast with the results of Zhang et al., Jin et al. and Ren et al., published in the last two years. Zhang reported lycorine as having potent anti-SARS-CoV-2 activity with an EC_50_ of 0.18 µM and no cytotoxicity (CC_50_ > 40 µM) [31]. Similarly, lycorine displayed a highly potent activity (EC_50_ = 0.878 ± 0.022 µM in Vero cells, CC_50_ > 10 µM on HEK293) according to Jin et al. [32]. Also in 2021, Ren et al. again reported a high potency of lycorine (EC_50_ = 0.439 ± 0.122 µM) in the Vero-E6 cell and varying cytotoxicity values in Vero-E6 (CC_50_ > 1000 µM), Huh-7 (CC_50_ = 0.834 ± 0.0630 µM), HEK293T (CC_50_ = 1.044 ± 0.0734 µM) [33]. Although these antiviral assays also run in Vero-E6 cells, the RT-PCR read-out was after 18–24 h, whereas we used a high-content imaging read-out after 5 days, which might explain the increased cytotoxicity in our assay. Compounds **2**, **3,** and **9**, ((±)-2-*epi*-pseudolycorine and ungeremine, respectively) lacked antiviral activity, as well as cytotoxicity. Detailed results can be found as Supplementary Materials.

**Figure 5:**
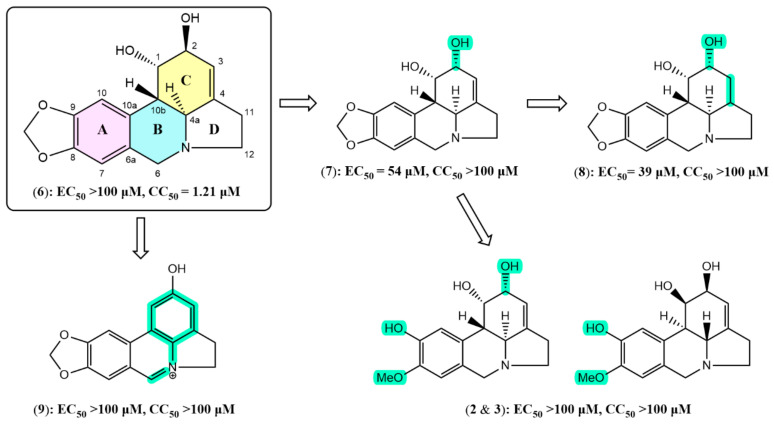
Structure-activity relationship of the lycorine-type alkaloids: lycorine (**6**), 2-*epi*-lycorine (**7**), zephyranthine (**8**), ungeremine (**9**), (±)-2-*epi*-pseudolycorine (**2** and **3**). Structural differences compared to lycorine are highlighted in green.

Preliminary structure-activity relationship (SAR) was examined for six lycorine-type alkaloids isolated in this study (Figure 5). Using lycorine as the standing stone, 2-*epi*-lycorine only has one stereocenter at C-2 that differs from lycorine, but this entirely inverted its activity as well as its cytotoxicity compared to lycorine with an EC_50_ of 54 µM at non-cytotoxic concentration (CC_50_ > 100 µM). This suggested that ligand-host interaction occurs at ring C in the structure of 2-*epi*-lycorine, and that the chirality of C-2 plays an important role in improving the potency and removing the cytotoxicity on the Vero-E6 cell line. Furthermore, an enhancement of activity was observed for zephyranthine (EC_50_ = 39 µM, CC_50_ > 100 µM) compared to 2-*epi*-lycorine implying that the double bond at C-3 and C-4 is not necessary for the activity. The disappearance of the double bond increases the flexibility of ring C, and thus could favorize the formation of interactions between two hydroxyl groups and residues at the binding site. On the other hand, the opening of the dioxymethylene moiety at C-8 and C-9 resulted in the loss of the antiviral activity of (±)-2-*epi*-pseudolycorine in comparison with 2-*epi*-lycorine. Looking closely at their structures, oxygen attached to C-9 can solely be a hydrogen-bonding acceptor when the dioxymethylene moiety remains, but 9-OH in the case of (±)-2-*epi*-pseudolycorine is a phenolic hydroxyl which could participate in an ion interaction and/or hydrogen bonding as either a hydrogen-bonding acceptor or a hydrogen-bonding donor. Therefore, 9-OH might generate unfavorable interactions with residues at the active site, which in turn cancels the antiviral activity. This suggested that ring A, especially substituent at C-9, might be involved in the pharmacophore for the antiviral activity. Another observation is that aromatization of rings B and C as can be seen in the structure of ungeremine eliminated cytotoxicity on the Vero-E6 cell line, but did not improve its anti-SARS-CoV-2 potency.

Our findings are in agreement with previously reported SAR studies about anticancer activity and the inhibitory effects of lycorine-type alkaloids against dengue and hepatitis C viruses [34,35,36,37]. In general, ring C with substitution on C-1 and C-2 is of key importance for the activities and selectivity; and next comes to the role of substitution on ring A. However, with the limited structures in the current study, the effects of chirality at C-1 and different types of substituents are not covered. This remains an interesting question to be addressed in the future.

## 3. Materials and Methods

### 3.1. General Experimental Procedure

Analytical-grade solvents, including methanol (MeOH), dichloromethane (DCM), ethyl acetate (EtOAc), *n*-butanol (*n*-BuOH), ethanol (EtOH) and acetonitrile (ACN) were purchased from Acros Organics (Geel, Belgium) or from Fisher Scientific (Loughborough, UK). UPLC-grade methanol, acetonitrile and formic acid were purchased from Biosolve (Dieuze, France). All reagents: ammonia 25% (NH_4_OH 25%), glacial acetic acid (AA 99.8%), dimethyl sulfoxide (DMSO), formic acid (FA 98%), hydrochloric acid 25% (HCl 25%), potassium iodide (KI 99%), diethyl amine (DEA 99%) and sodium nitrite (NaNO_2_ 99.5%) were purchased from either Acros Organics (Geel, Belgium) or Sigma-Aldrich (St. Louis, MO, USA). Bismuth (III) nitrate (Bi(NO_3_)_3_ 99.5%) was purchased from Merck (Darmstadt, Germany). MiliQ water was obtained from a Direct-Pure Up Ultrapure and RO water system (Rephile Bioscience, Ghent, Belgium). Deuterated solvents used in NMR experiments (methanol-*d_4_* (CD_3_OD—99.8% D) and dimethyl sulfoxide-*d_6_* ((CD_3_)_2_SO—99.9% D) were purchased from Sigma-Aldrich (Merck, Germany).

TLC was performed on pre-coated silica gel F254 plates (Merck, Darmstadt, Germany), and the spots were observed under UV light (254 and 366 nm). After subsequently spraying with the Dragendorff and NaNO_2_ 10% reagents, TLC plates were observed under visible light. Dragendorff reagent was prepared by combining a mixture A and a solution B. Mixture A was prepared by suspending 0.85 g of bismuth subnitrate (Bi_5_O(OH)_9_(NO_3_)_4_) in 40 mL of water and 10 mL of glacial acetic acid. Solution B was prepared by dissolving 8 g of potassium iodide (KI) in 20 mL of water.

The following instruments were used for fractionation, isolation and structure elucidation. For flash chromatography, a Reveleris iES system from Grace (Columbia, MD, USA) using the Reveleris Navigator^TM^ v.1 software and commercially packed Claricep flash columns containing 40 g irregular deactivated silica gel (Agela Technologies, Wilmington, DE, USA). For analytical purposes, an Agilent 1200 series HPLC-DAD system (Agilent Technologies, Santa Clara, CA, USA) equipped with OpenLAB v.A.01.05 software was used, together with a Phenomenex Kinetex EVO C18 (250 × 4.6 mm, 5 µm) column. For purification, a preparative HPLC system coupled with DAD and MS detectors was used. All compartments of this system were supplied by Waters (Milford, MA, USA), including MassLynx v.4.1 software. Separation was carried out on a semi-preparative Phenomenex Kinetex C18 column (250 × 10 mm, 5 µm). NMR spectra were recorded on two different NMR instruments (400 MHz): an Avance Nanobay III and a DRX-400 system (Bruker BioSpin, Rheinstetten, Germany). NMR data processing was performed with TopSpin v.4.0.6 from Bruker. Finally, accurate mass measurements were conducted for all isolated compounds using a Xevo G2-XS QToF mass spectrometer (Waters, Milford, MA, USA) coupled to an Acquity UPLC system and an Acquity HSS T3 UPLC column (100 × 2.1 mm, 1.8 μm) was used. Optical rotation were recorded on a Jasco P-2000 spectropolarimeter (Easton, MD, USA) at 589 nm (20 °C). The system was equipped with Spectra Manager^TM^ software. 

### 3.2. Plant Material

The fresh bulbs of *Hymenocallis littoralis* (Jacq.) Salisb. were collected in April 2017 in Dubréka (Guinea-Conakry) and provided by Prof. A. M. Baldé (University Gamal Abdel Nasser of Conakry, Guinea). The voucher specimen (No. 2aHK2) is kept at the Institute for Research and Development of Medicinal and Food Plants of Guinea, Dubréka. After cleaning and lyophilization, the total weight of the plant material was 3.3 kg.

### 3.3. Extraction and Isolation

Freeze-dried, powdered bulbs were macerated and percolated with approximately 30 L of 80% methanol. The extract was filtered and dried under reduced pressure. Then, liquid-liquid partition was applied in order to obtain subfractions. In short, the crude extract was suspended in water and was acidified with 10% HCl to a pH < 3 before performing liquid-liquid partition with DCM (I). Next, the pH of the acidified phase was increased to a pH ≥ 9 by adding NH_4_OH (25%), followed by a subsequent liquid-liquid partition with DCM (II), EtOAc and *n*-BuOH. In this way, four fractions (DCM (I), DCM (II), EtOAc and *n*-BuOH) were obtained and were examined by TLC with the mobile phase DCM-MeOH-NH_4_OH (90:10:1). Observation of the TLC plates after spraying with the Dragendorff + NaNO_2_ 10% reagents indicated that all fractions contained alkaloids, and the majority of alkaloids were present in the DCM (I) fraction.

The DCM (I) (3.1 g) and DCM (II) extracts (3.0 g) were fractionated by flash chromatography. After TLC investigation, silica gel was chosen as the suitable stationary phase. The percentage of NH_4_OH was fixed at 0.1% in the solvent systems. Other parameters were set as follows: (1)—Detectors: ELSD and UV (254 and 270 nm), (2)—Flow rate: 25 mL/min. The step-wise gradient used for fractionating the DCM (I) and DCM (II) extracts was: 0–5 min (0% MeOH), 5–15 min (5% MeOH), 15–30 min (10% MeOH), 30–40 min (15% MeOH), 40–45 min (20% MeOH), 45–50 min (25% MeOH), 55–60 min (70% MeOH), and the gradient was held when a peak was eluted. As for the fractionation of the EtOAc extract (2.2 g), column chromatography was used with a sample/stationary phase (silica gel) ratio of 1/50. Isocratic elution was applied, using DCM-MeOH-NH_4_OH (90/10/1, *v*/*v*) as the solvent system, by virtue of the result of TLC analysis. Finally, MeOH was used to elute any remaining compounds of interest from the column. The *n*-BuOH fraction (4.6 g) was also fractionated by column chromatography on a silica gel column. On the basis of TLC investigation, a three-step gradient was chosen to elute compounds from the column: DCM-MeOH-H_2_O-NH_4_OH (80:20:10:1, *v*/*v*), DCM-MeOH-H_2_O-NH_4_OH (60:40:10:1, *v*/*v*), and DCM-MeOH-H_2_O-NH_4_OH (40:60:10:1, *v*/*v*). Finally, MeOH was used to elute any remaining compounds of interest from the column.

Then, purification was carried out using semi-preparative HPLC-DAD-MS, silica gel column chromatography and recrystallization. Compounds **2** and **3** (2.4 mg), **4** (4.0 mg), **5** (4.5 mg), **8** (3.0 mg) and **11** (6.0 mg) were isolated by semi-preparative HPLC, of which compounds **2**, **3**, and **8** originated from the *n*-BuOH extract, compounds **4** and **5** from the DCM (I) extract and compound **11** from the DCM (II) extract. 

With regard to the recrystallization, in the current work, impure crystals were first collected and were subsequently rinsed with DCM, DCM-MeOH (99:1, *v*/*v*) and DCM-MeOH (95:5, *v*/*v*) to remove impurities. Next, the crystals were dissolved in a minimal volume of DCM-MeOH (50:50, *v*/*v*). Finally, the solution obtained was left to dry in open air, resulting in the formation of solid crystals of the desired constituent. In this way, 1.0 g and 18.0 mg were purified of compounds **6** and **10**, respectively. Compound **6** was found to be the most abundant alkaloid and was present in the DCM (II) and EtOAc extracts, and compound **10** was only purified from the EtOAc extract.

Compounds **1** (5.8 mg), **7** (1.5 mg), and **9** (1.5) were purified by silica gel column chromatography. Compound **1** was isolated from the *n*-BuOH extract and compounds **7** and **9** from the DCM (II) extract. The solvent systems used were deduced from TLC analysis. In the case of compound **1**, the elution was performed with DCM-MeOH-H_2_O-NH_4_OH (80:20:5:1, *v*/*v*), followed by DCM-MeOH-H_2_O-NH_4_OH (60:40:5:1, *v*/*v*). In the case of compounds **7** and **9**, the elution was carried out with DCM-MeOH-NH_4_OH (90:10:1, *v*/*v*), followed by DCM-MeOH-NH_4_OH (70:30:1, *v*/*v*).

Chiral HPLC separation was performed for compounds **2** and **3** on a Daicel Chiralpak IB column (250 × 4.6 mm) (Chiral Technologies Europe, Iilkirch Cedex, France), eluted with solvent A—MeOH:DEA (100:0.1, *v*/*v*) and solvent B—EtOH:DEA (100:0.1, *v*/*v*) using the following gradient: 0–10 min (50%B), 10–36 min (50–80% B); flow rate 1.0 mL/min. 

*O*-dimethyl-norlycoramine (**1**)

White amorphous powder (5.8 mg); UV λ_max_ 210, 254 nm; ${\left[\alpha \right]}_{D}^{25}$ −63.6 (*c* 0.9, MeOH) and ${\left[\alpha \right]}_{D}^{25}$ −60.2 (*c* 0.10, MeOH); ^1^H and ^13^C NMR in DMSO-*d_6_* (400 and 100 MHz): see Table 1; Positive HRESIMS *m/z* 262.1446 [M+H]^+^ (calcd for C_15_H_20_NO_3_, 262.1443)

(±)-2-*epi*-pseudolycorine (**2** and **3**)

Yellowish amorphous powder (2.4 mg); UV λ_max_ 210, 245, 300 nm; ${\left[\alpha \right]}_{D}^{25}$ −8.0 (*c* 0.4, MeOH); ^1^H and ^13^C NMR in DMSO-*d_6_* (400 and 100 MHz): see Table 4; Positive HRESIMS *m/z* 290.1392 [M+H]^+^ (calcd for C_16_H_20_NO_4_, 290.1392)

### 3.4. Computational Details

Conformational analysis was performed by PCMODEL (version 10.0) using the Monte Carlo algorithm and the MMFF94 force field. All conformers within an energy window of 10 kcal.mol^−1^ were selected. Specific optical rotations were simulated for the sodium D-line wavelength (589.3 nm), as experimental optical rotations were measured at the same wavelength. The obtained conformers were first geometrically optimized in the gas phase. After dereplication, Boltzmann weighting was applied using the sum of electronic and thermal free energies at 298.15 K, and only conformers with energies within an energy window of 2.5 kcal.mol^−1^ from the global minimum were considered as the contributing ones.

For DP4+ probability, the B3LYP/6-31G(d)//mPW1PW91/6-311+G(d,p) level was applied to simulate shielding tensors [38]. On the other hand, two theory levels were used to compute specific optical rotation: B3LYP/6-31++G(d,p)//6-311++G(3df,2dp) and B3LYP/6-31++G(d,p)//aug-cc-pVTZ, as recommended by Yu (2012) and Stephens (2001), respectively [39,40]. The polarizable continuum model using methanol as solvent was also performed to improve the accuracy of shielding tensors and OR calculations. Coupling constants were simulated in gas-phase at the B3LYP/6-31G(d)//B3LYP/6-31G(d,p) level reported by Bally and Rablen which considers only the Fermi contact as the main contributor [41]. All the above quantum chemical calculations were performed by Gaussian16. Detailed information about the results of DP4+ probability and 3D coordinates of computed conformers can be found as Supplementary Materials.

### 3.5. Anti-SARS-CoV-2 Assay

#### 3.5.1. Cells and Virus

VeroE6-eGFP cells (provided by Dr. K. Andries, J&JPRD, Beerse, Belgium) were maintained in DMEM (Gibco cat no. 41965-039) supplemented with heat-inactivated 10% FBS and 500 µg/mL Geneticin (Gibco cat no. 10131-0275) and kept in a humidified 5% CO_2_ incubator at 37 °C. For the production of virus stocks and during virus experiments, the cells were maintained in an assay medium. The VeroE6-eGFP assay medium consisted of DMEM supplemented with 2% FBS.

The SARS-CoV-2 isolate used in this study was the BetaCov/Belgium/GHB-03021/2020 (EPI ISL407976|2020-02-03), which was isolated from a Belgian patient returning from Wuhan in February 2020. The isolate was passaged seven times on Vero-E6 cells, which introduced two series of amino acid deletions in the spike protein [42]. The infectious content of the virus stock was determined by titration on Vero-E6 cells. The SARS-CoV-2-related work was conducted in the high-containment BSL3+ facilities of the KU Leuven Rega Institute (3CAPS) under licenses AMV 30112018 SBB 219 2018 0892 and AMV 23102017 SBB 219 2017 0589 according to institutional guidelines.

#### 3.5.2. Antiviral Assay

The SARS-CoV-2 antiviral assay in Vero-E6 cells is derived from a previously established SARS-CoV-1 assay [43]. Stock solutions of the various compounds in DMSO (10 mM) were prepared. On day -1, the test compounds were serially diluted in assay medium and VeroE6-eGFP cells were plated corresponding to a final density of 25,000 cells per well in black 96-well plates (Greiner Bio-One, Vilvoorde, Belgium; Catalog 655090). The plates were incubated overnight (37 °C, 5% CO_2_ and 95% relative humidity). On day 0, the cells with compound were infected with SARS-CoV-2 (at 20 CCID_50_ per well). The plates were incubated in a humidified incubator at 37 °C and 5% CO_2_. At 4 days p.i., the wells were examined for eGFP expression using an argon laser-scanning microscope. The microscope settings were excitation at 488 nm and emission at 510 nm, and the fluorescence images of the wells were converted into signal values. The antiviral activity was expressed as EC_50_ defined as the concentration of compound achieving 50% inhibition of the virus-reduced eGFP signals as compared to the untreated virus-infected control cells. The cytotoxicity of the compounds for VeroE6 cells in the absence of virus was evaluated in a standard MTS-assay as described previously [44].

## 4. Conclusions

The phytochemical investigation of the bulbs of *H. littoralis* with advanced spectroscopic techniques led to the isolation of eleven Amaryllidaceae alkaloids. Three previously undescribed alkaloids, namely *O*-demethyl-norlycoramine (**1**), (−)-2-*epi*-pseudolycorine (**2**), (+)-2-*epi*-pseudolycorine (**3**), together with eight known alkaloids (**4**–**11**). The relative configurations of all compounds were unambiguously determined by combining *J*-coupling and NOE effects.

In vitro anti-SARS-CoV-2 screening revealed weak anti-SARS-CoV-2 activity of *O*-demethyl-norlycoramine, 2-*epi*-lycorine, 6α-hydroxyhippeastidine, 6β-hydroxyhippeastidine, zephyranthine, and 9-*O*-demethyl-7-*O*-methyllycorenine (EC_50_ = 40–77 µM) at non-cytotoxic concentrations (CC_50_ > 100 µM). Lycorine and pancratistatin exhibited cytotoxicity with a CC_50_ of 1.2 µM and 0.13 µM, respectively. Investigation of the structure-activity relationship for the lycorine-type alkaloids in this study suggested a crucial role of ring C: the meaningful ligand-host interactions appeared to be related to ring C; the spatial flexibility of ring C was required to exhibit anti-SARS-CoV-2 activity; the stereochemistry of C-2 on ring C might determine the activity and cytotoxicity. In view of the limited scope of this study, the chirality effect of C-2 was explored, but that of C-1 remains unknown. Therefore, further research on a bigger library of lycorine-type analogs with a wide chemical diversity is necessary. Besides, the potential of galanthamine-type and crinine-type scaffolds as anti-SARS-CoV-2 agents remains a compelling research topic.

## Figures and Tables

**Table 1:** Experimental and computed NMR spectroscopic data (400 MHz, CD_3_OD) for *O*-demethyl-norlycoramine (**1**).

	Experimental		Calculated	
Position	δ_C_, Type	δ_H_ (*J* in Hz)	δ_C_	δ_H_
1	24.4, CH_2_	1.67 m	25.1	1.53
1	24.4, CH_2_	1.85 *	25.1	2.11
2	26.9, CH_2_	1.81 *	26.9	2.23
2	26.9, CH_2_	1.45 m	26.9	1.15
3	64.7, CH	4.09 m	64.2	3.91
4	31.2, CH_2_	2.00 *	29.2	2.08
4	31.2, CH_2_	2.32 m	29.2	2.33
4a	88.9, CH	4.31 t (3.2)	90.2	4.50
6	52.7, CH_2_	3.83 d (15.0)	50.3	3.71
6	52.7, CH_2_	3.97 d (15.0)	50.3	3.95
6a	128.9, C	-	135.1	-
7	120.6, CH	6.51 d (8.0)	120.0	6.52
8	114.9, CH	6.55 d (8.0)	112.2	6.57
9	141.1, C	-	140.5	-
10	146.0, C	-	144.5	-
10a	135.5, C	-	134.5	-
10b	47.2, C	-	49.2	-
11	36.5, CH_2_	1.75 *	39.7	1.71
11	36.5, CH_2_	1.98 *	39.7	1.83
12	46.5, CH_2_	3.26 m	44.4	3.20
12	46.5, CH_2_	3.19 m	44.4	3.19
CMAE			1.8	0.13
Max. outlier			6.2	0.42

* overlapping signals.

**Table 2:** DP4+ probabilities of compounds **1**–**3** and computed *J*-couplings of compounds **2** and **3** estimated using CD_3_OD as solvation model. For compound 1, stereocenters’ order is (4a,3,10b). For compounds **2** and **3**, stereocenters’ order is (1,2,4a,10b).

Compound 1		Compounds 2 and 3		
Diastereomer	Probability (%)	Diastereomer	Probability (%)	J_H-4a/H-10b_
SRR	26.78	SRRR	0	10.69
SRS	0	SRRS	61.05	5.52
SSR	73.22	SRSR	0	7.36
SSS	0	SRSS	0	10.56
		SSRR	0	10.25
		SSRS	10.15	5.78
		SSSR	0	7.99
		SSSS	28.80	10.85

**Table 3:** Experimental and computed optical rotation values for compounds **1**–**3**.

	Compound 1		Compounds 2 and 3	
Experimental	−63.6		−8.0	
B3LYP/6-31++G(d,p)// 6-311++G(3df,2dp)	SSR RRS	−48.8 48.7	SRSS RSRR	−230.4 230.6
B3LYP/6-31++G(d,p)// aug-cc-pVTZ	SSR RRS	−46.8 46.6	SRSS RSRR	−227.7 227.9

**Table 4:** Experimental and computed NMR spectroscopic data (400 MHz, CD_3_OD and (CD_3_)_2_SO for (±)-2-*epi*-pseudolycorine (**2** and **3**).

	In CD_3_OD				In (CD_3_)_2_SO	
	Experimental		Calculated		Experimental	
Position	Δ_c_, Type	δ_H_ (*J* in Hz)	δ_C_	δ_H_	δ_C_	δ_H_
1	71.6, CH	4.49 s	75.1	3.94	70.8, CH	4.21 s
2	72.9, CH	4.19 *	71.2	4.31	72.2, CH	3.96 s
3	119.9, CH	5.59 s	126.7	5.62	118.9, CH	5.35 s
4	n.o.	-		-	142.4, C	-
4a	62.6, CH	3.06 d (11.0)	59.9	2.96	61.4, CH	2.58 d (10.6)
6α	57.0, CH_2_	4.19 *	56.4	4.03	57.0, CH_2_	3.99 d (13.8)
6β	57.0, CH_2_	3.71 d (14.1)	56.4	3.57	57.0, CH_2_	3.30 d (13.8)
6a	127.2, C	-	129.3	-	127.5, C	-
7	111.7, CH	6.76 s	107.8	6.73	111.6, CH	6.63 s
8	147.8, C	-	145.2	-	146.1, C	-
9	146.8, C	-	145.4	-	145.2, C	-
10	112.7, CH	6.88 s	114.2	7.07	112.4, CH	6.70 s
10a	128.8, C	-	128.5	-	129.1, C	-
10b	40.5, CH	2.76 d (11.1)	41.6	3.05	40.2, CH	2.49 *
11	29.3, CH_2_	2.68 * (2H)	31.3	2.70	28.5, CH_2_	2.46 * (2H)
12α	54.8, CH_2_	3.40 *	53.1	3.38	53.9, CH_2_	3.20 t (8.2)
12β	54.8, CH_2_	2.67 *	53.1	2.61	53.9, CH_2_	2.19 q (8.4)
8-OMe	56.5, CH_3_	3.84 s	54.5	3.97	56.2, CH_3_	3.71 s
CMAE			2.3	0.13		
Max. outlier			6.8	0.55		

* overlapping signals. n.o. = not observed.

## Data Availability

Data are contained within the article or the Supplementary Materials.

## Supplementary Materials

The following supporting information can be downloaded at: https://www.mdpi.com/article/10.3390/molecules28073222/s1. The Supporting Information File contains the NMR, HRMS and DP4+ probability results (Figures S1–S23).

## Sample Availability

Samples of the compounds **1**–**3** are available from the authors.

## References

[1] Aniszewski T. (2015). Alkaloids: Chemistry, Biology, Ecology, and Applications.

[2] Jin Z. (2005). Amaryllidaceae and Sceletium Alkaloids. Nat. Prod. Rep..

[3] Takos A.M., Rook F. (2013). Towards a Molecular Understanding of the Biosynthesis of Amaryllidaceae Alkaloids in Support of Their Expanding Medical Use. Int. J. Mol. Sci..

[4] Marco L., Carreiras C. (2006). Galanthamine, a Natural Product for the Treatment of Alzheimer’s Disease. Recent Pat. CNS Drug Discov..

[5] Berkov S., Osorio E., Viladomat F., Bastida J. (2020). Chemodiversity, Chemotaxonomy and Chemoecology of Amaryllidaceae Alkaloids.

[6] National Plant Data Center, NRCS, USDA The Plants Database: Hymenocallis littoralis (Jacq.) Salisb. https://www.itis.gov/servlet/SingleRpt/SingleRpt?search_topic=TSN&search_value=507014#null.

[7] Jaume B., Strahil B., Laura T. (2011). Chemical and Biological Aspects of Amaryllidaceae Alkaloids. Recent Adv. Pharm. Sci..

[8] Singh G., Saxena R.K. (2017). Chemistry and Medicinal Properties of *Hymenocallis littoralis*. Int. J. Sci. Res..

[9] Ding Y., Qu D., Zhang K.M., Cang X.X., Kou Z.N., Xiao W., Zhu J.B. (2017). Phytochemical and Biological Investigations of Amaryllidaceae Alkaloids: A Review. J. Asian Nat. Prod. Res..

[10] Chen N., Ji Y., Zhang W., Xu Y., Yan X., Sun Y., Song H., Xu C., Cai L., Zheng H. (2016). Chemical Constituents from *Hymenocallis littoralis*. Lett. Org. Chem..

[11] Ji Y.B. (2015). Research Progress on Chemical Compositions of *Hymenocallis littoralis*. Medicine Sciences and Bioengineering.

[12] Lin L.Z., Hu S.F., Chai H.B., Pengsuparp T., Pezzuto J.M., Cordell G.A., Ruangrungsi N. (1995). Lycorine Alkaloids from *Hymenocallis littoralis*. Phytochemistry.

[13] Ma W., Wang S., Wang Y., Zeng J., Xu J., He X. (2022). Antiproliferative Amaryllidaceae Alkaloids from the Bulbs of *Hymenocallis littoralis* (Jacq.) Salisb. Phytochemistry.

[14] Le N.T.H., Vermeyen T., Aerts R., Herrebout W.A., Pieters L., Tuenter E. (2023). Epimeric Mixture Analysis and Absolute Configuration Determination Using an Integrated Spectroscopic and Computational Approach—A Case Study of Two Epimers of 6-Hydroxyhippeastidine from *Hymenocallis littoralis*. Molecules.

[15] Ingrassia L., Lefranc F., Mathieu V., Darro F. (2008). Amaryllidaceae Isocarbostyril Alkaloids and Their Derivatives as Promising Antitumor Agents. Transl. Oncol..

[16] Fürst R. (2016). Narciclasine–an Amaryllidaceae Alkaloid with Potent Antitumor and Anti-Inflammatory Properties. Planta Med..

[17] Ndibwami A. (2007). A General Synthesis of Phenanthridinone Alkaloids. Synlett.

[18] Ji Y.B., Chen N., Zhu H.W., Ling N., Li W.L., Song D.X., Gao S.Y., Zhang W.C., Ma N.N. (2014). Alkaloids from Beach Spider Lily (*Hymenocallis littoralis*) Induce Apoptosis of HepG-2 Cells by the Fas-Signaling Pathway. Asian Pac. J. Cancer Prev..

[19] Nair J.J., van Staden J. (2022). Antiviral Alkaloid Principles of the Plant Family Amaryllidaceae. Phytomedicine.

[20] Majnooni M.B., Fakhri S., Bahrami G., Naseri M., Farzaei M.H., Echeverría J. (2021). Alkaloids as Potential Phytochemicals against SARS-CoV-2: Approaches to the Associated Pivotal Mechanisms. Evid. Based Complement. Altern. Med..

[21] Raman K., Rajagopal K., Islam F., Dhawan M., Mitra S., Aparna B., Varakumar P., Byran G., Choudhary O.P., Emran T. (2022). Bin. Role of Natural Products towards the SARS-CoV-2: A Critical Review. Ann. Med. Surg..

[22] Christy M.P., Uekusa Y., Gerwick L., Gerwick W.H. (2021). Natural Products with Potential to Treat RNA Virus Pathogens Including SARS-CoV-2. J. Nat. Prod..

[23] Chakravarti R., Singh R., Ghosh A., Dey D., Sharma P., Velayutham R., Roy S., Ghosh D. (2021). A Review on Potential of Natural Products in the Management of COVID-19. RSC Adv..

[24] Van Breemen R.B., Muchiri R.N., Bates T.A., Weinstein J.B., Leier H.C., Farley S., Tafesse F.G. (2022). Cannabinoids Block Cellular Entry of SARS-CoV-2 and the Emerging Variants. J. Nat. Prod..

[25] Welsch J.T., Smalley T.B., Matlack J.K., Avalon N.E., Binning J.M., Johnson M.P., Allcock A.L., Baker B.J., Tuaimenals B.-H. (2022). Merosesquiterpenes from the Irish Deep-Sea Soft Coral Duva Florida with Bioactivity against Cervical Cancer Cell Lines. J. Nat. Prod..

[26] Cordell G.A. (2006). The Alkaloids: Chemistry and Biology, Volume 63.

[27] Cedrón J.C., Del Arco-Aguilar M., Estévez-Braun A., Ravelo Á.G. (2010). Chapter 1—Chemistry and Biology of Pancratium Alkaloids.

[28] Dewick P.M. (2002). Medicinal Natural Products: A Biosynthetic Approach.

[29] Marcarino M.O., Cicetti S., Zanardi M.M., Sarotti A.M. (2022). A Critical Review on the Use of DP4+ in the Structural Elucidation of Natural Products: The Good, the Bad and the Ugly. A Practical Guide. Nat. Prod. Rep..

[30] Grimblat N., Gavín J.A., Hernández Daranas A., Sarotti A.M. (2019). Combining the Power of J Coupling and DP4 Analysis on Stereochemical Assignments: The J-DP4 Methods. Org. Lett..

[31] Zhang Y.N., Zhang Q.Y., Li X.D., Xiong J., Xiao S.Q., Wang Z., Zhang Z.R., Deng C.L., Yang X.L., Wei H.P. (2020). Gemcitabine, Lycorine and Oxysophoridine Inhibit Novel Coronavirus (SARS-CoV-2) in Cell Culture. Emerg. Microbes Infect..

[32] Jin Y.H., Min J.S., Jeon S., Lee J., Kim S., Park T., Park D., Jang M.S., Park C.M., Song J.H. (2021). Lycorine, a Non-Nucleoside RNA Dependent RNA Polymerase Inhibitor, as Potential Treatment for Emerging Coronavirus Infections. Phytomedicine.

[33] Ren P., Shang W., Yin W., Ge H., Wang L., Zhang X., Li B., Li H., Xu Y., Xu E.H. (2022). A Multi-Targeting Drug Design Strategy for Identifying Potent Anti-SARS-CoV-2 Inhibitors. Acta Pharmacol. Sin..

[34] Roy M., Long L., Xiaojuan X., Peifu F., Mao Y., Jing L. (2018). Lycorine: A Prospective Natural Lead for Anticancer Drug Discovery. Biomed. Pharmacother..

[35] Nair J.J., Van Staden J. (2014). Cytotoxicity Studies of Lycorine Alkaloids of the Amaryllidaceae. Nat. Prod. Commun..

[36] Ji Y.B., Yang S.L., Zhang X.L., Liu Y.J. (2017). Review on the Structure Modification of Lycorine. IOP Conf. Ser. Earth Environ. Sci..

[37] Wang P., Li L.F., Wang Q.Y., Shang L.Q., Shi P.Y., Yin Z. (2014). Anti-Dengue-Virus Activity and Structure-Activity Relationship Studies of Lycorine Derivatives. ChemMedChem.

[38] Grimblat N., Zanardi M.M., Sarotti A.M. (2015). Beyond DP4: An Improved Probability for the Stereochemical Assignment of Isomeric Compounds Using Quantum Chemical Calculations of NMR Shifts. J. Org. Chem..

[39] Yu J., Cao Y., Song H., Wang X., Yao S. (2012). Calculations of Optical Rotation: Influence of Molecular Structure. J. Serb. Chem. Soc..

[40] Stephens P.J., Devlin F.J., Cheeseman J.R., Frisch M.J. (2001). Calculation of Optical Rotation Using Density Functional Theory. J. Phys. Chem. A.

[41] Bally T., Rablen P.R. (2011). Quantum-Chemical Simulation of 1H NMR Spectra. 2. Comparison of DFT-Based Procedures for Computing Proton-Proton Coupling Constants in Organic Molecules. J. Org. Chem..

[42] Boudewijns R., Thibaut H.J., Kaptein SJ F., Li R., Vergote V., Seldeslachts L., Van Weyenbergh J., De Keyzer C., Bervoets L., Sharma S. (2020). STAT2 Signaling Restricts Viral Dissemination but Drives Severe Pneumonia in SARS-CoV-2 Infected Hamsters. Nat. Commun..

[43] Ivens T., Van Den Eynde C., Van Acker K., Nijs E., Dams G., Bettens E., Ohagen A., Pauwels R., Hertogs K. (2005). Development of a Homogeneous Screening Assay for Automated Detection of Antiviral Agents Active against Severe Acute Respiratory Syndrome-Associated Coronavirus. J. Virol. Methods.

[44] Jochmans D., Leyssen P., Neyts J. (2012). A Novel Method for High-Throughput Screening to Quantify Antiviral Activity against Viruses That Induce Limited CPE. J. Virol. Methods.

