# Supplementary material for: Anti-SARS-CoV-2 Activity and Cytotoxicity of Amaryllidaceae Alkaloids from Hymenocallis littoralis
Source: Molecules. 2023 Apr 4;28(7):3222. doi: 10.3390/molecules28073222 (PMC10096449; doi:10.3390/molecules28073222)
Supplement: Supplementary file 1 [file molecules-28-03222-s001.zip › molecules-2293393-supplementary.pdf]

SUPPORTING INFORMATION

Anti-SARS-CoV-2 Activity and Cytotoxicity of Amaryllidaceae Alkaloids from *Hymenocallis littoralis*

Ngoc-Thao-Hien Le[1], Steven De Jonghe[2], Kristien Erven[2], Tom Vermeyen[3,4], Aliou M. Baldé[5], Wouter A. Herrebout[3], Johan Neyts[2], Christophe Pannecouque[2], Luc Pieters[1], Emmy Tuenter[1]*

[1]Natural Products & Food Research and Analysis (NatuRA), Department of Pharmaceutical Sciences, University of Antwerp, Universiteitsplein 1, 2610 Antwerp, Belgium.

[2]KU Leuven, Department of Microbiology, Immunology and Transplantation, Rega Institute for Medical Research, Laboratory of Virology and Chemotherapy, Herestraat 49, postbus 1043, B-3000 Leuven, Belgium

[3]Molecular Spectroscopy (MolSpec), Department of Chemistry, University of Antwerp, Groenenborgerlaan 171, 2020 Antwerp, Belgium

[4]Ghent Quantum Chemistry Group, Department of Chemistry, Ghent University, Krijgslaan 281, B-9000 Ghent, Belgium

[5]Department of Pharmacy, University Gamal Abdel Nasser of Conakry, Guinea

*lengocthaohien@gmail.com or ngocthaohien.le@uantwerpen.be

TABLE OF CONTENTS

1. NMR data of compounds 6-11 3

2. HRMS and NMR spectra of compound **1** in CD_3OD 6

3. HRMS and NMR spectra of compounds **2** and **3** in CD_3OD and $(CD_3)_2SO$ 10

4. DP4+ probability results 17

5. VCD spectrum of compounds **2** and **3** 105

6. Anti-SARS-CoV-2 activity and cytotoxicity 106

1. NMR data of compounds 6-11

*Lycorine (**6**)*

White crystals (1 g); UV λ_{max} 205, 240, 290 nm; ^{1}H and ^{13}C NMR in $(CD_3)_2SO$ (400 and 100 MHz): δ_H 6.80 (s, H-10), 6.67 (s, H-7), 5.95 (s, H-13a), 5.94 (s, H-13b), (5.39 (brs, H-3), 4.87 (d, 6.26, 2-OH), 4.76 (d, 4.27, 1-OH), 4.24 (brs, H-1), 4.02 (d, 14.3, H-6α), 3.97 (brs, H-2), 3.32 (d, 14.3, H-6β), 3.20 (t, 8.3, H-12α), 2.60 (d, 10.3, H-4a), 2.50 (overlapped, H-10b), 2.45-2.49 (overlapped, H-11, 2H), 2.20 (q, 8.3, H-12β); δ_C 145.0 (C-9), 144.7 (C-8), 141.1 (C-4), 129.1 (C-6a), 128.9 (C-10a), 117.9 (C-3), 106.5 (C-7), 104.5 (C-10), 71.2 (C-2), 69.6 (C-1), 60.2 (C-4a), 56.1 (C-6), 52.7 (C-12), 39.6 (C-10b), 27.6 (C-11); Positive HRESIMS *m/z* 288.1238 $[M+H]^+$ (calcd for $C_{16}H_{18}NO_4$, 288.1236)

*2-Epi-lycorine (**7**)*

White powder (1.5 mg); UV λ_{max} 205, 254, 290 nm; $[\alpha]_D^{25}$ −6.5 (*c* 0.2, MeOH); ^{1}H and ^{13}C NMR in $(CD_3)_2SO$ (400 and 100 MHz): δ_H 6.80 (s, H-10), 6.67 (s, H-7), 5.95 (s, H-13a), 5.94 (s, H-13b), 5.39 (brs, H-3), 4.87 (d, 6.26, 2-OH), 4.76 (d, 4.27, 1-OH), 4.24 (brs, H-1), 4.02 (d, 14.3, H-6α), 3.97 (brs, H-2), 3.32 (d, 14.3, H-6β), 3.20 (t, 8.3, H-12α), 2.60 (d, 10.3, H-4a), 2.50 (overlapped, H-10b), 2.45-2.49 (overlapped, H-11, 2H), 2.20 (q, 8.3, H-12β); δ_C 145.0 (C-9), 144.7 (C-8), 141.1 (C-4), 129.1 (C-6a), 128.9 (C-10a), 117.9 (C-3), 106.5 (C-7), 104.5 (C-10), 71.2 (C-2), 69.6 (C-1), 60.2 (C-4a), 56.1 (C-6), 52.7 (C-12), 39.6 (C-10b), 27.6 (C-11); Positive HRESIMS *m/z* 288.1232 $[M+H]^+$ (calcd for $C_{16}H_{18}NO_4$, 288.1236)

*Zephyranthine (**8**)*

White powder (3 mg); UV λ_{max} 210, 254 nm; $[\alpha]_D^{25}$ −14.6 (*c* 0.8, MeOH); ^{1}H and ^{13}C NMR in CD_3OD (400 and 100 MHz): δ_H 6.97 (s, H-10), 6.65 (s, H-7), 5.90 (d, 1.1, H-13a), 5.89 (d, 1.1, H-13b), 4.50 (brs, H-1), 4.15 (H-6α), 3.89 (m, H-2), 3.72 (H-6β), 3.26 (H-12α), 3.07 (H-4a),

2.87 (H-12β), 2.56 (H-10b), 2.53 (H-4), 2.09 (H-3α), 1.93 (overlapped, H-11, 2H), 1.86 (H-3β); δ_C 148.1 (C-8), 147.2 (C-9), 132.4 (C-6a), 128.7 (C-10a), 107.7 (C-7), 106.2 (C-10), 102.0 (C-13), 69.9 (C-1), 69.6 (C-2), 60.0 (C-4a), 54.9 (C-12), 54.1 (C-6), 38.4 (C-4), 37.0 (C-10b), 29.6 (C-3), 28.1 (C-11); Positive HRESIMS *m/z* 290.1389 $[M+H]^+$ (calcd for $C_{16}H_{18}NO_4$, 290.1392)

*Ungeremine (**9**)*

White powder (1.5 mg); UV λ_{max} 254, 365 nm; $[\alpha]_D^{25}$ −1.8 (*c* 0.2, MeOH); ^1H and ^{13}C NMR in $(CD_3)_2SO$ (400 and 100 MHz): δ_H 8.92 (brs, H-6), 7.93 (s, H-10), 7.53 (s, H-7), 7.22 (d, 1.5, H-1), 7.10 (d, 1.5, H-3), 6.31 (s, H-13, 2H), 5.11 (t, 7.0, H-12, 2H), 3.64 (t, 7.0, H-11, 2H); Positive HRESIMS *m/z* 266.0816 $[M+H]^+$ (calcd for $C_{16}H_{18}NO_4$, 266.1817)

*Pancratistatin (**10**)*

Brownish crystals (18 mg); UV λ_{max} 210, 254 nm; $[\alpha]_D^{25}$ −1.5 (*c* 0.2, MeOH); ^1H and ^{13}C NMR in $(CD_3)_2SO$ (400 and 100 MHz): δ_H 13.07 (s, 7-OH phenol), 7.50 (s, N-H), 6.49 (s, H-10), 6.06 (d, 0.9, H-11a), 6.04 (d, 0.9, H-11b), 5.35 (d, 3.97, 3-OH), 5.08 (d, 6.00, 2-OH), 4.82 (d, 7.77, 1-OH), 4.28 (m, H-1), 3.96 (m, H-3), 3.85 (m, H-2), 3.73 (m, H-4), 3.71 (m, H-4a), 2.97 (dd; 11.9, 1.4; H-10b); δ_C 169.5 (C-6), 152.1 (C-9), 145.4 (C-7), 135.7 (C-10a), 131.7 (C-8), 107.5 (C-6a), 101.5 (C-11), 97.5 (C-10), 73.1 (C-2), 69.9 (C-3), 68.2 (C-1), 50.3 (C-4a), 39.5 (C-10b); Positive HRESIMS *m/z* 326.0873 $[M+H]^+$ (calcd for $C_{16}H_{18}NO_4$, 326.0876)

*9-O-Demethyl-7-O-methyllycorenine (**11**)*

Yellowish amorphous powder (6 mg); UV λ_{max} 205, 230, 280 nm; $[\alpha]_D^{25}$ 126.7 (*c* 0.5, MeOH); ^1H and ^{13}C NMR in $(CD_3)_2SO$ (400 and 100 MHz): δ_H 6.95 (s, H-11), 6.75 (s, H-8), 5.64 (m, H-4), 5.46 (s, H-7), 4.26 (dd; 6.0, 1.7; H-5a), 3.88 (s, 13-OMe), 3.50 (s, 7-OMe), 3.22 (m, H-2), 2.88 (d, 9.6, H-11c), 2.65 (m, H-5), 2.46 (dd; 9.8, 1.8; H-11b), 2.38 (t, H-2), 2.31 (m, H-5),

2.14 (s, N-Me); δ_C 147.4 (C-10), 146.6 (C-9), 140.4 (C-3a), 128.5 (C-7a), 126.2 (C-11a), 116.8 (C-4), 114.1 (C-8), 98.1 (C-7), 67.9 (C-11c), 66.7 (C-5a), 56.0 (C-2), 55.3 (13-OMe), 54.1 (7-OMe), 43.2 (C-11b), 42.7 (N-Me), 31.1 (C-5), 28.6 (C-3); Positive HRESIMS *m/z* 318.1705 $[M+H]^+$ (calcd for $C_{16}H_{18}NO_4$, 318.1705)

2. HRMS and NMR spectra of compound **1** in CD_3OD

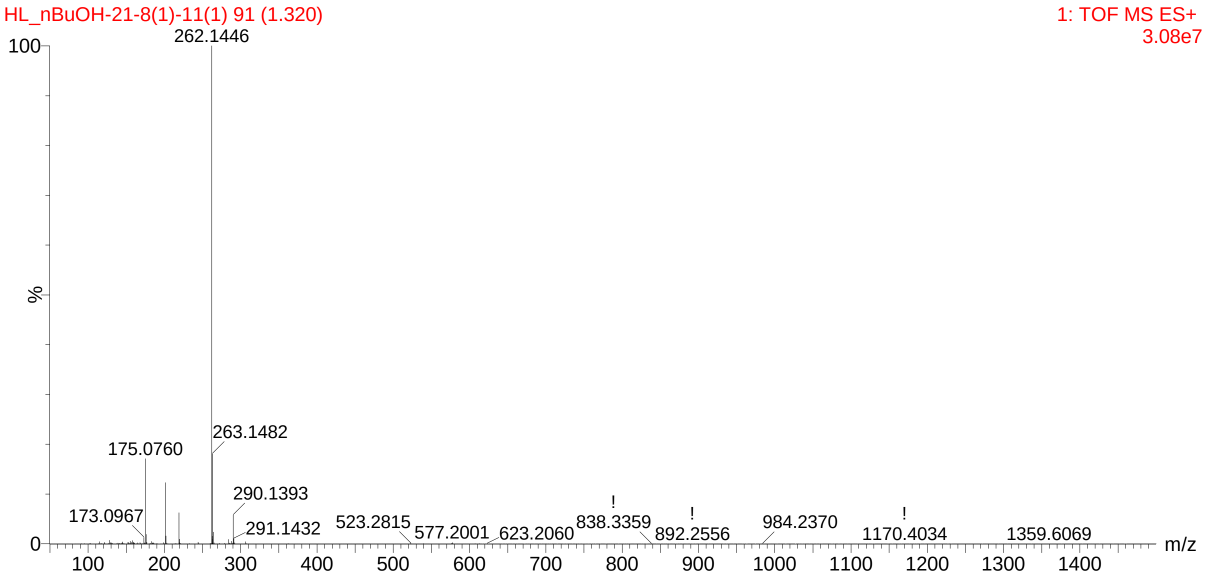

Figure S1. HRMS spectrum of compound **1**

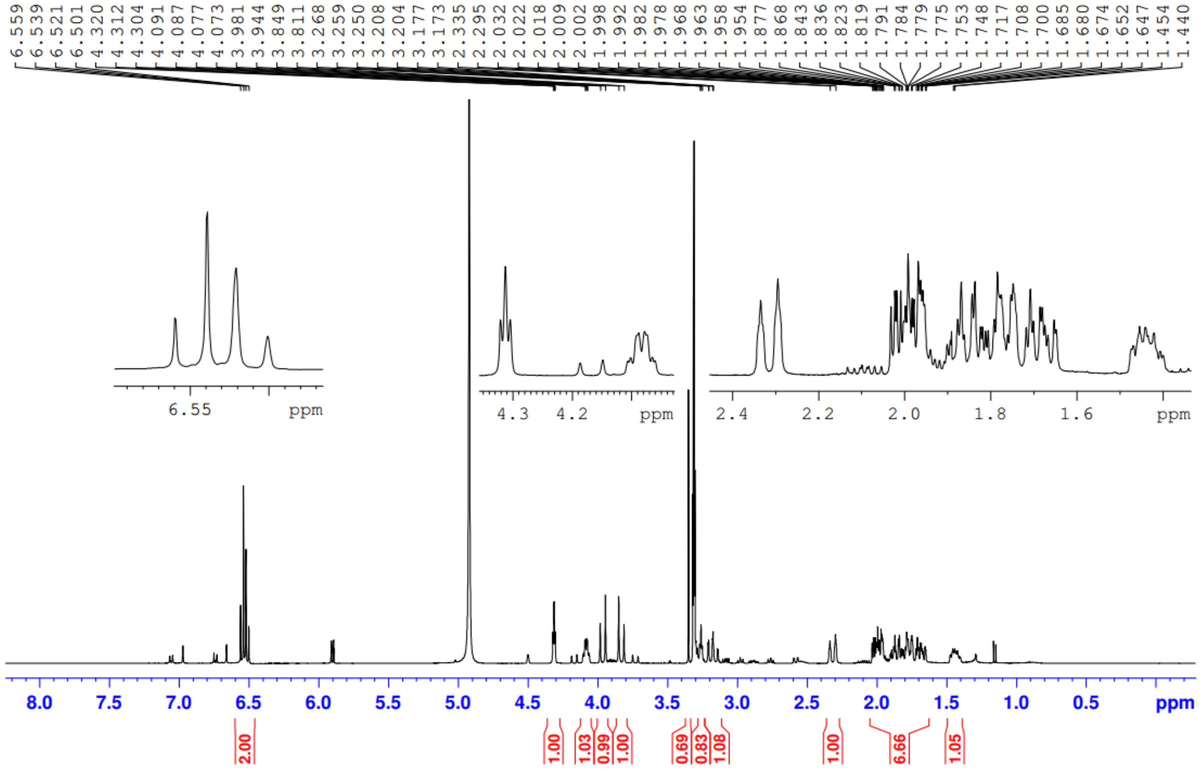

Figure S2. ¹H spectrum of compound **1** in CD_3OD

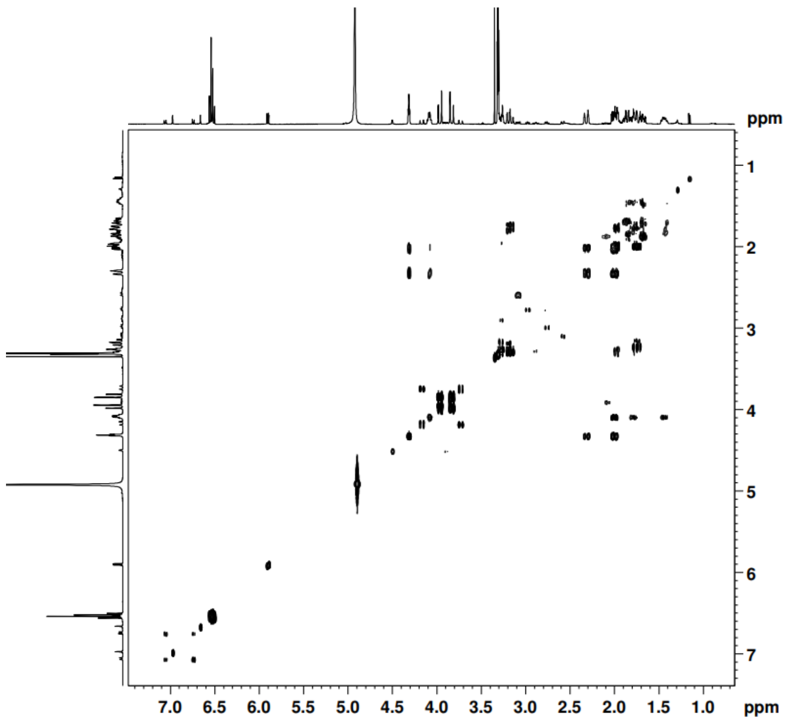

Figure S3. COSY spectrum of compound **1** in CD_3OD

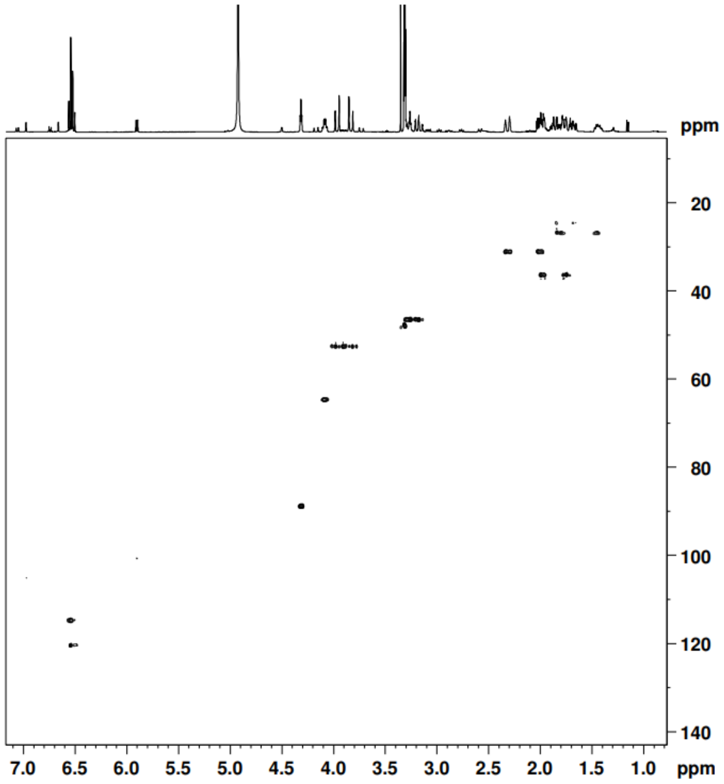

Figure S4. HSQC spectrum of compound **1** in CD_3OD

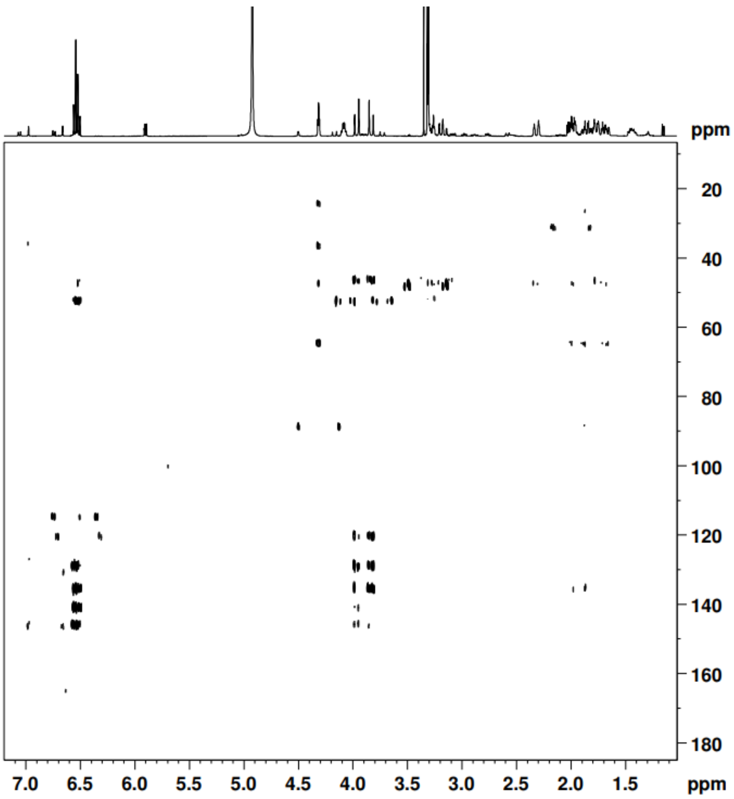

Figure S5. HMBC spectrum of compound **1** in CD_3OD

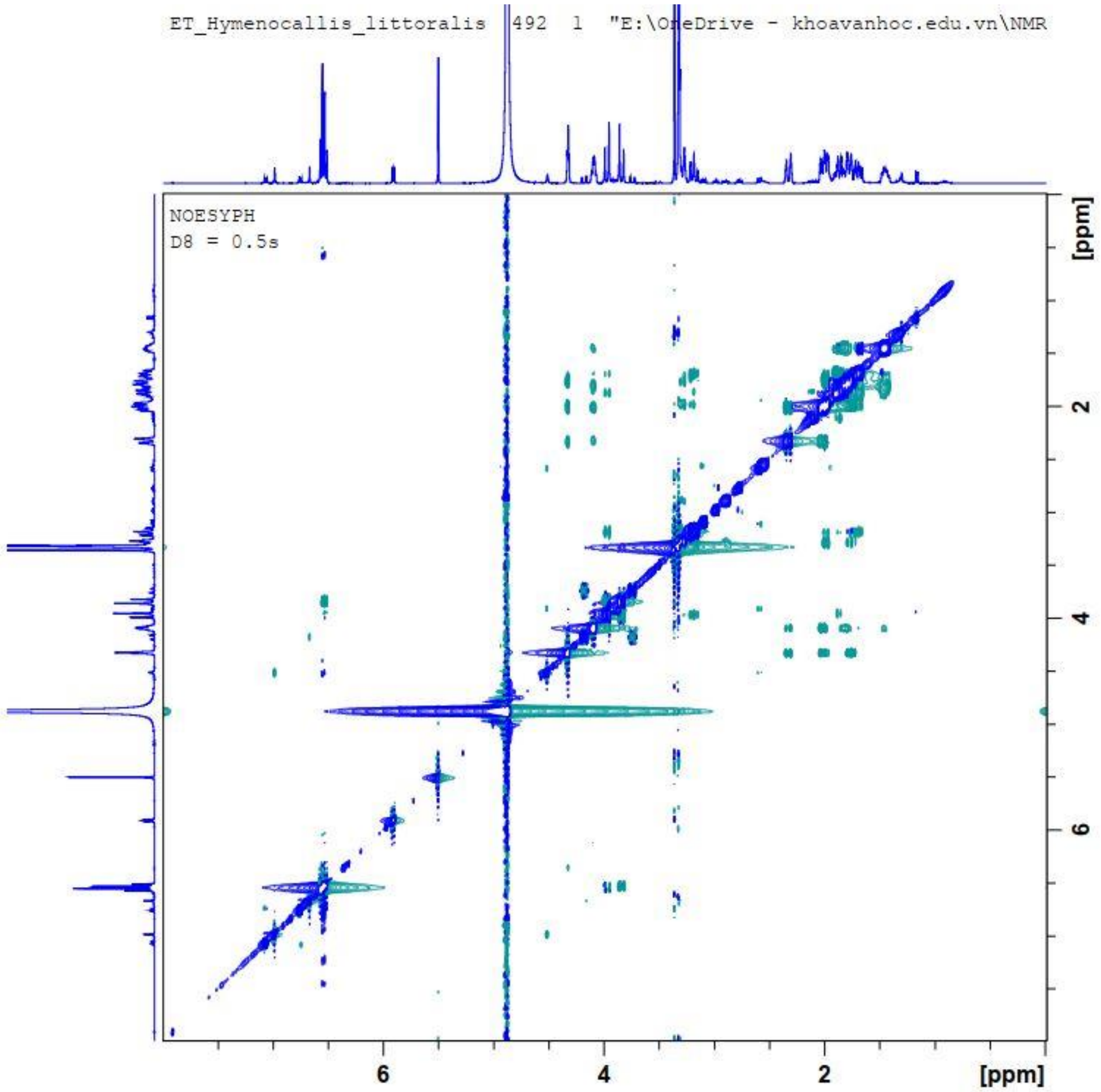

Figure S6. NOESY spectrum of compound **1** in CD_3OD

3. HRMS and NMR spectra of compounds **2** and **3** in CD_3OD and $(CD_3)_2SO$

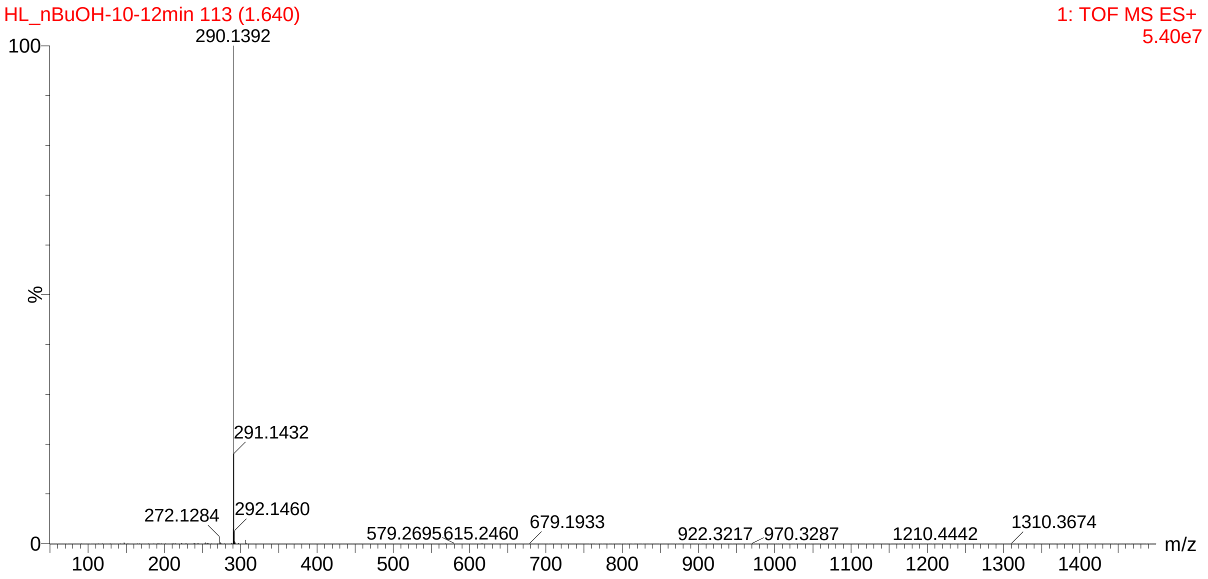

Figure S7. HRMS spectrum of compounds **2** and **3**

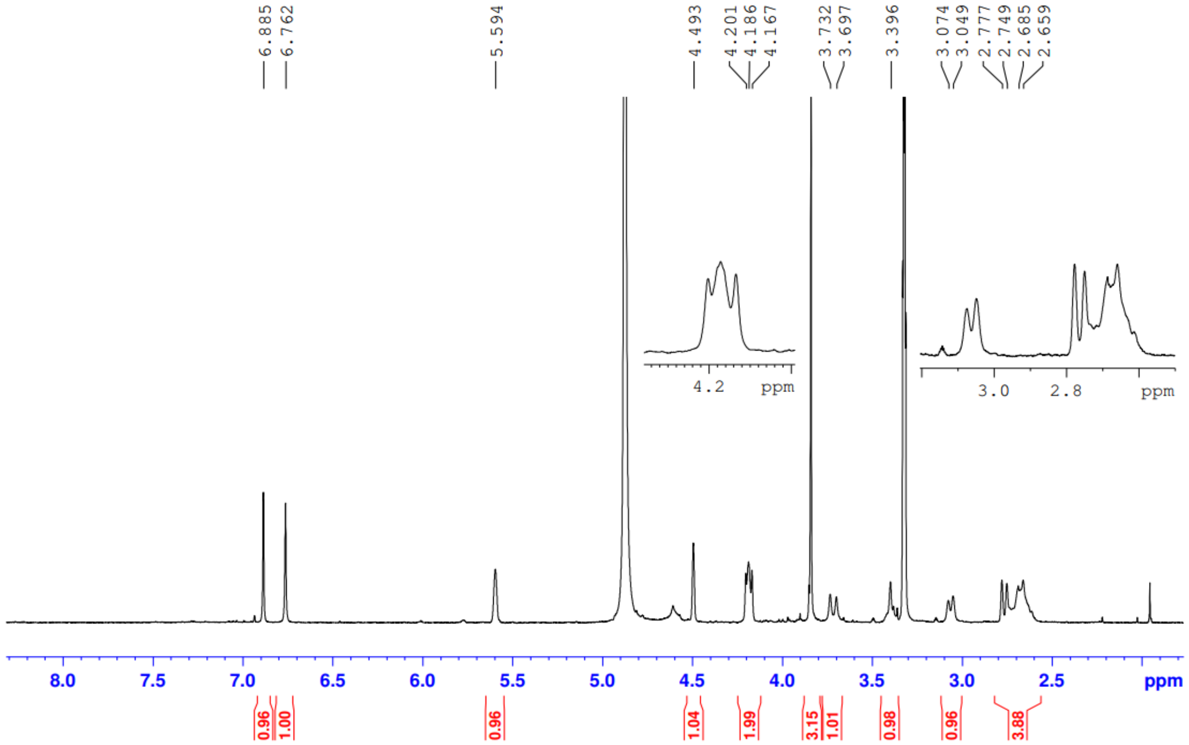

Figure S8. ^{1}H spectrum of compounds **2** and **3** in CD_3OD

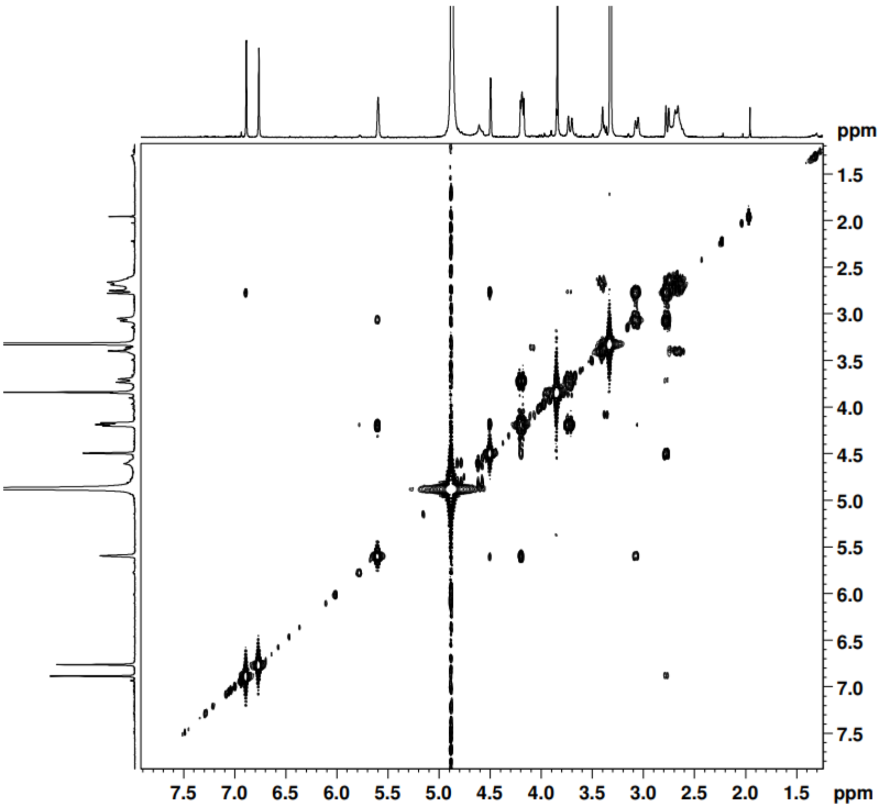

Figure S9. COSY spectrum of compounds **2** and **3** in CD_3OD

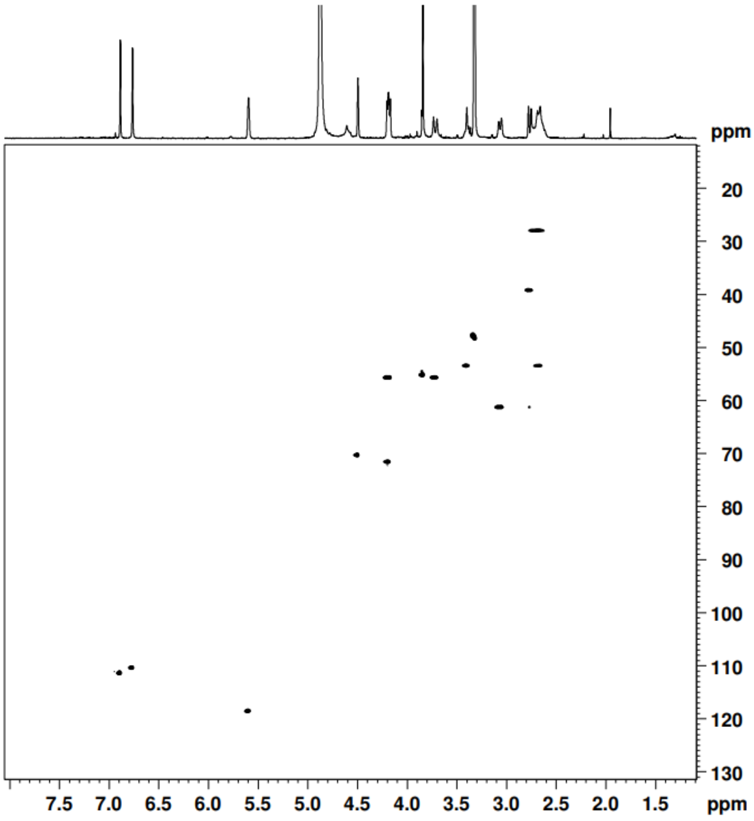

Figure S10. HSQC spectrum of compounds **2** and **3** in CD_3OD

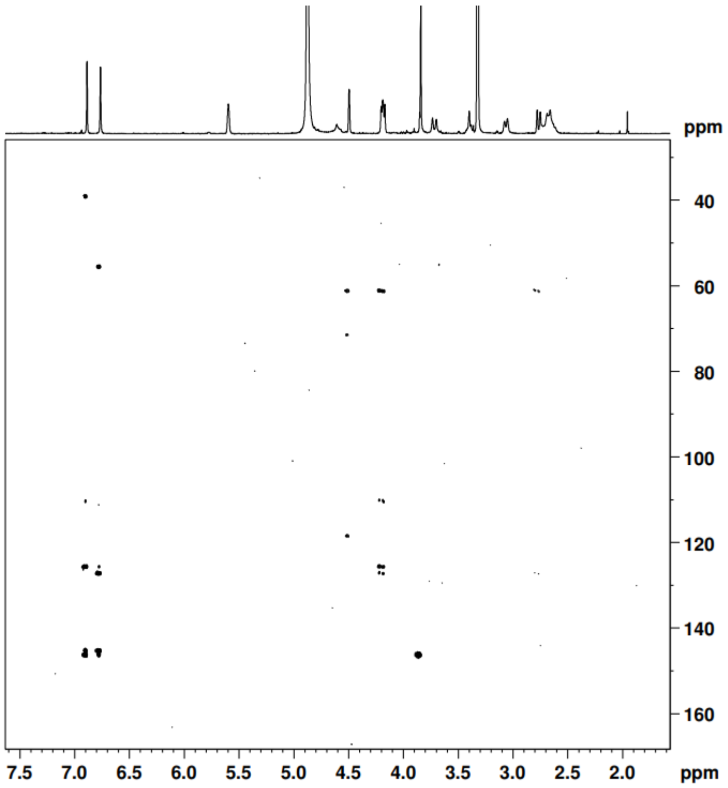

Figure S11. HMBC spectrum of compounds **2** and **3** in CD_3OD

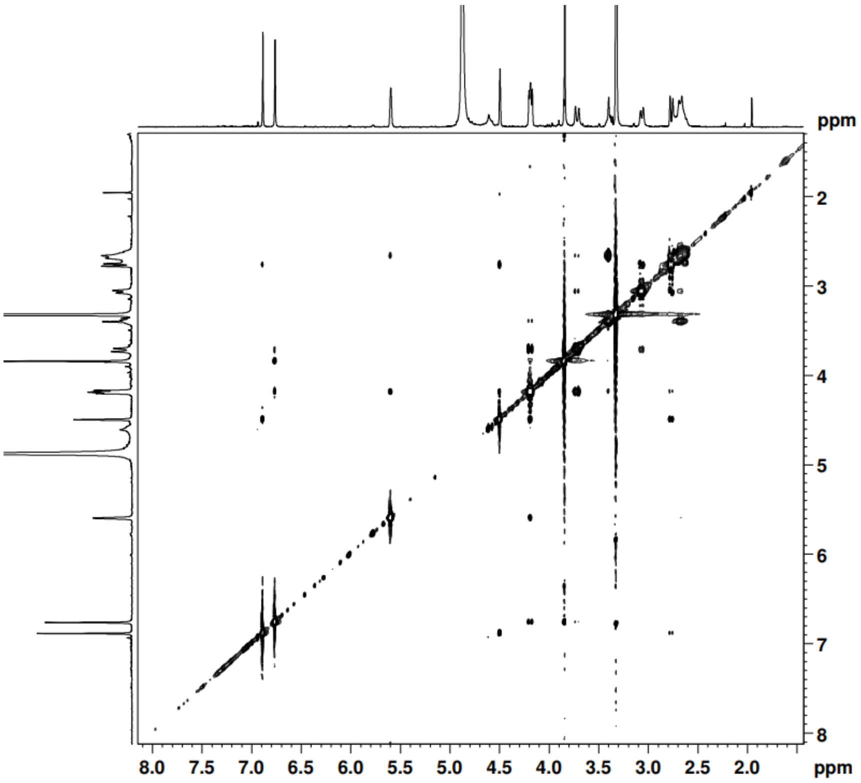

Figure S12. NOESY spectrum of compounds **2** and **3** in CD_3OD

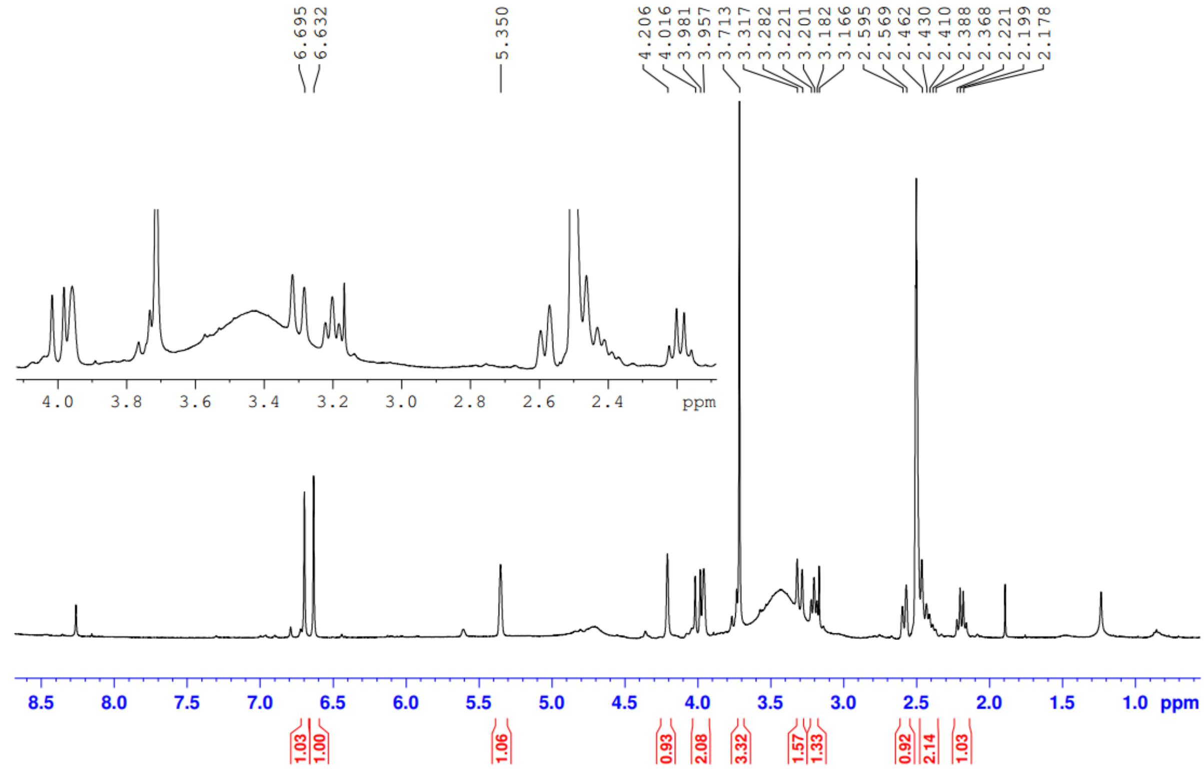

Figure S13. ^{1}H spectrum of compounds **2** and **3** in $(CD_3)_2SO$

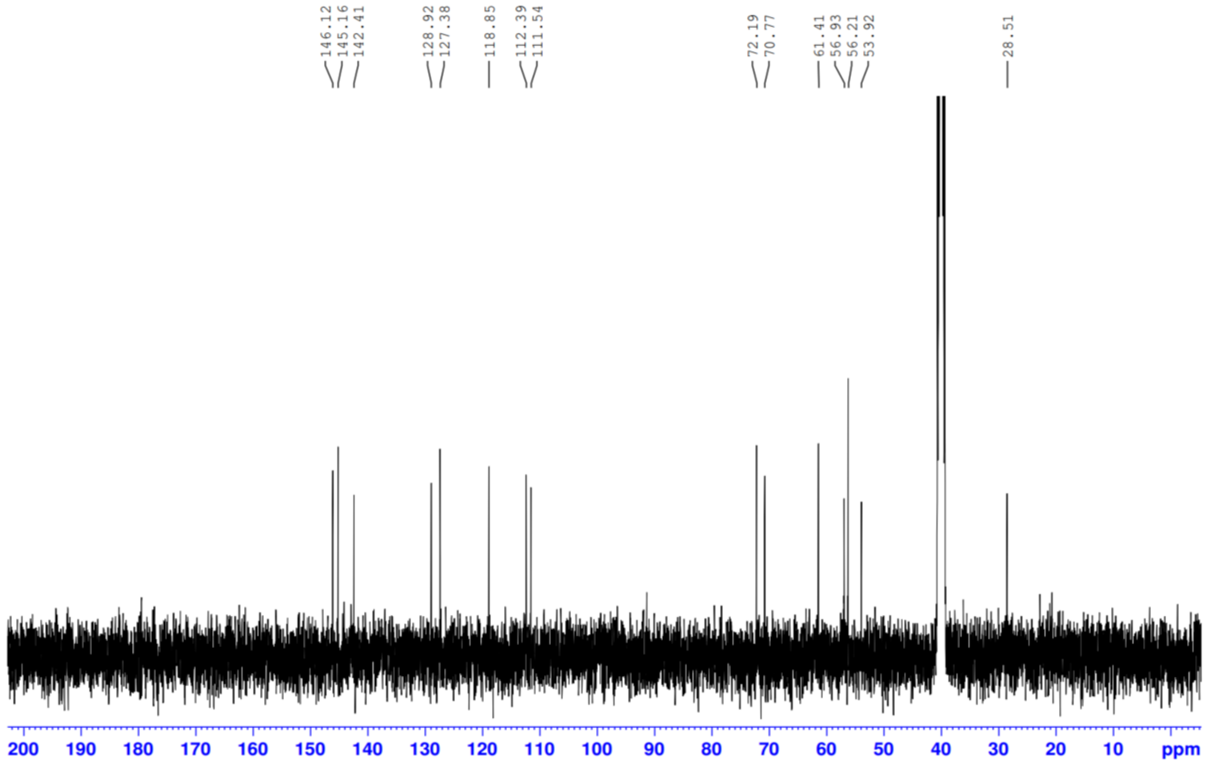

Figure S14. ^{13}C spectrum of compounds **2** and **3** in $(CD_3)_2SO$

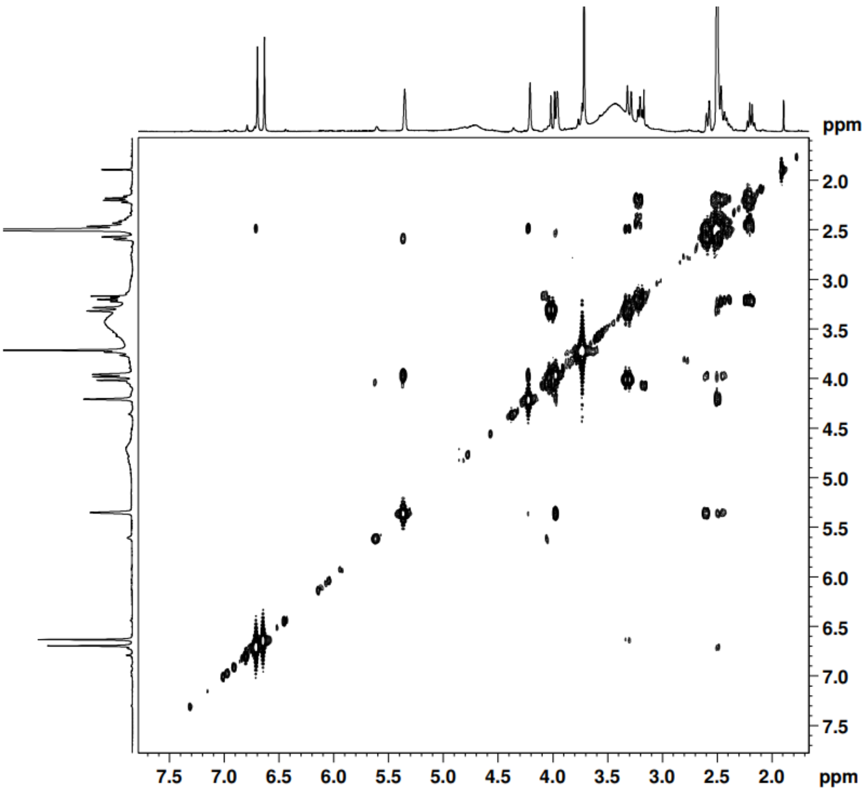

Figure S15. COSY spectrum of compounds **2** and **3** in $(CD_3)_2SO$

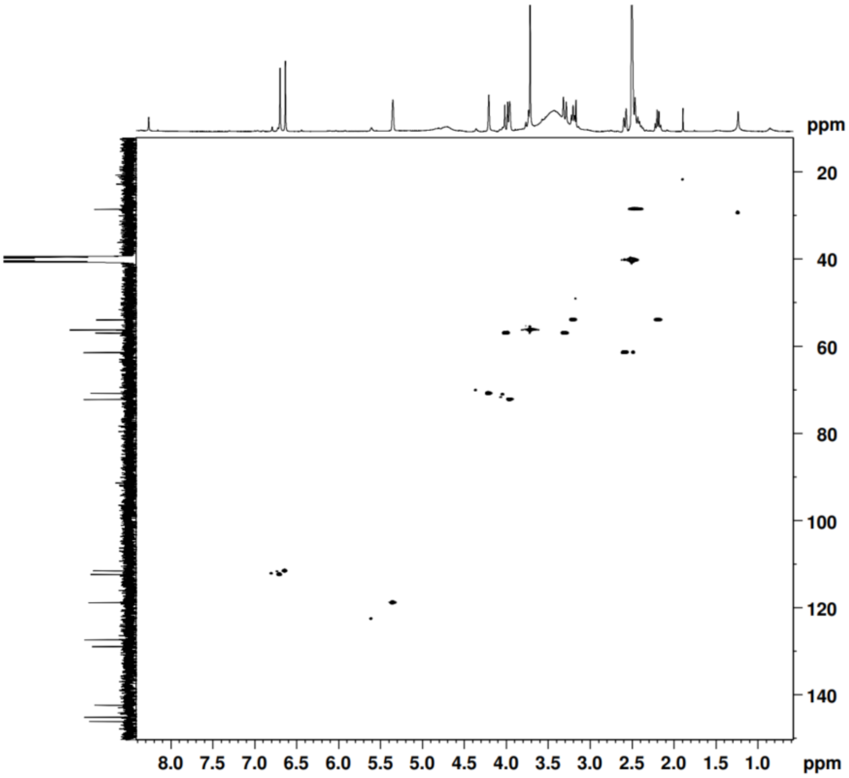

Figure S16. HSQC spectrum of compounds **2** and **3** in $(CD_3)_2SO$

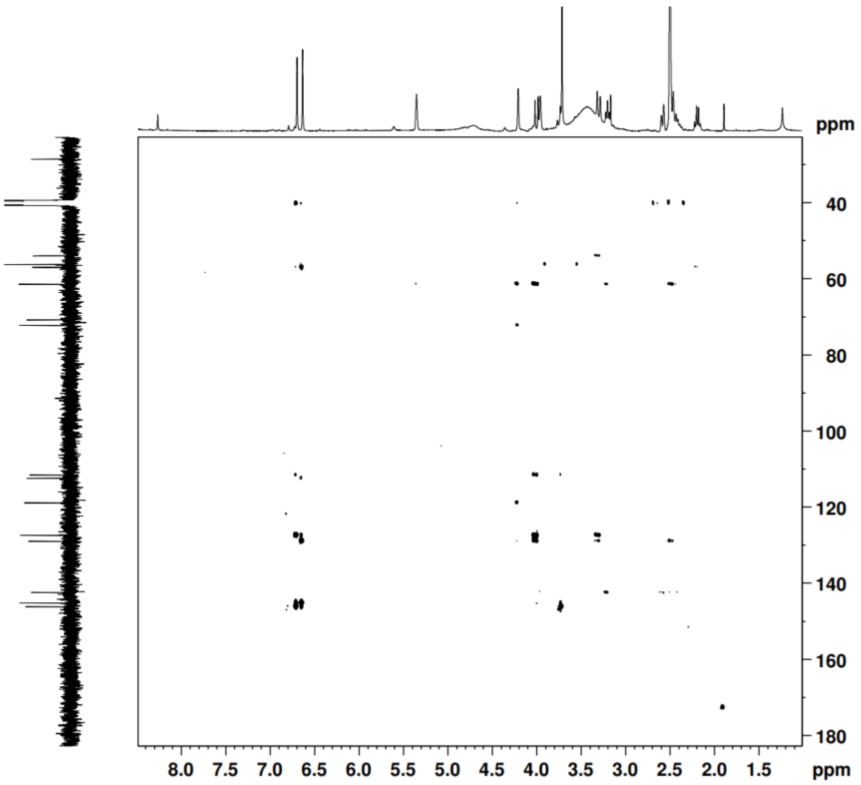

Figure S17. HMBC spectrum of compounds **2** and **3** in $(CD_3)_2SO$

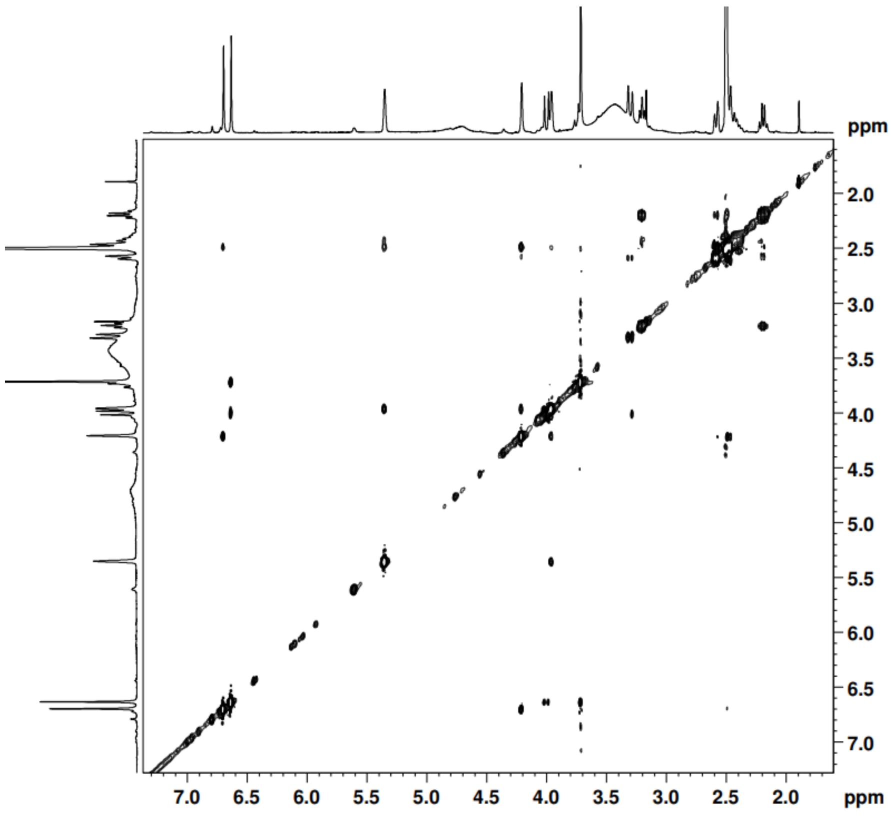

Figure S18. NOESY spectrum of compounds **2** and **3** in $(CD_3)_2SO$

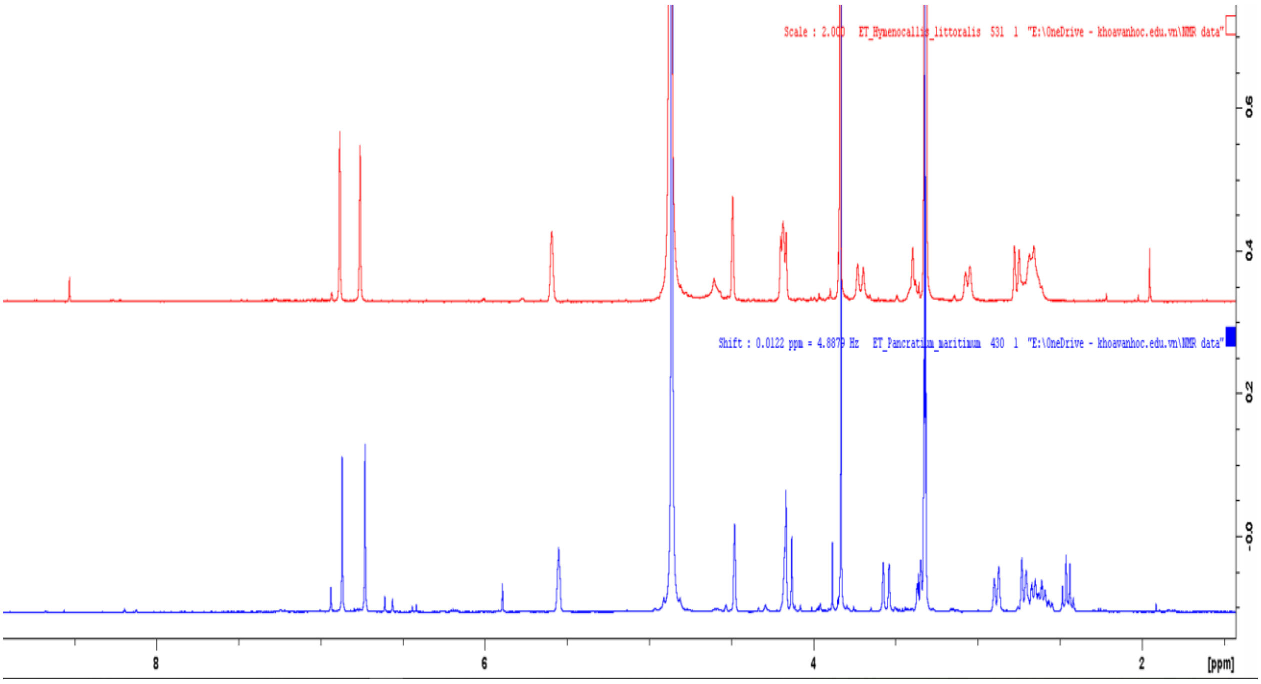

Figure S19. ^{1}H spectra of compounds **2** and **3** (in red) and pseudolycorine (in blue) in CD_3OD

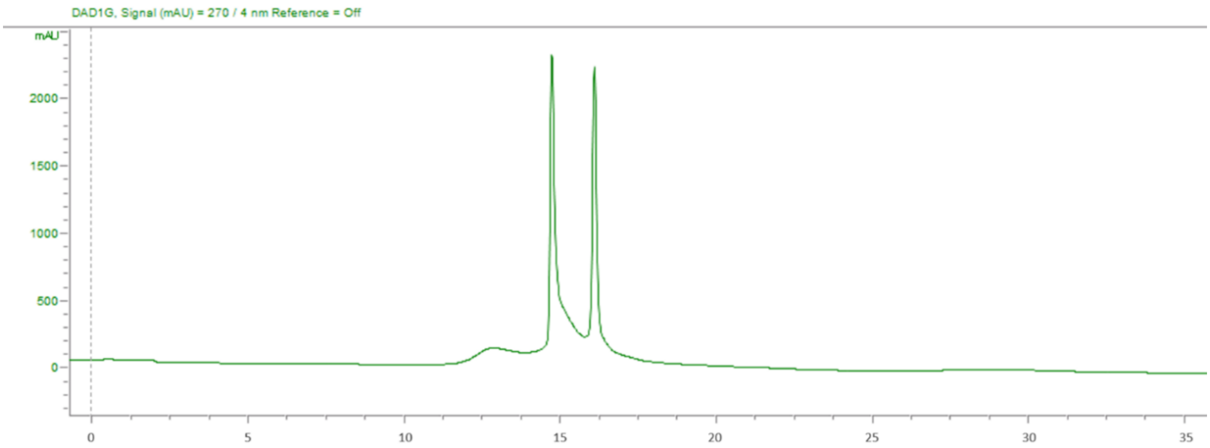

Figure S20. HPLC-UV chromatogram (270 nm) of compounds **2** and **3** after enantioseparation

4. DP4+ probability results

Functional	Solvent?	Basis Set	Type of Data
mPW1PW91	PCM	6-311+G(d,p)	Shielding Tensors

	Isomer 1	Isomer 2	Isomer 3	Isomer 4	Isomer 5	Isomer 6
sDP4+ (H data)	0.41%	0.00%	99.59%	0.00%	-	-
sDP4+ (C data)	93.96%	0.00%	6.04%	0.00%	-	-
sDP4+ (all data)	6.09%	0.00%	93.91%	0.00%	-	-
uDP4+ (H data)	4.76%	0.00%	95.24%	0.00%	-	-
uDP4+ (C data)	99.12%	0.00%	0.88%	0.00%	-	-
uDP4+ (all data)	84.94%	0.00%	15.06%	0.00%	-	-
DP4+ (H data)	0.02%	0.00%	99.98%	0.00%	-	-
DP4+ (C data)	99.94%	0.00%	0.06%	0.00%	-	-
DP4+ (all data)	26.78%	0.00%	73.22%	0.00%	-	-

Figure S21. DP4+ probability of compound **1** (*O*-Demethyl-norlycoramine)

Functional	Solvent?	Basis Set	Type of Data
mPW1PW91	PCM	6-311+G(d,p)	Shielding Tensors

	Isomer 1	Isomer 2	Isomer 3	Isomer 4	Isomer 5	Isomer 6	Isomer 7	Isomer 8
sDP4+ (H data)	0.00%	98.10%	0.00%	0.88%	0.69%	0.28%	0.00%	0.04%
sDP4+ (C data)	0.23%	0.14%	0.21%	0.07%	0.00%	13.79%	0.05%	85.52%
sDP4+ (all data)	0.00%	64.62%	0.00%	0.28%	0.00%	18.63%	0.00%	16.46%
uDP4+ (H data)	0.00%	68.59%	0.06%	0.14%	4.46%	26.20%	0.05%	0.50%
uDP4+ (C data)	92.15%	0.03%	0.00%	0.03%	0.03%	0.05%	0.00%	7.71%
uDP4+ (all data)	3.44%	27.67%	0.00%	0.05%	1.63%	15.95%	0.00%	51.25%
DP4+ (H data)	0.00%	99.84%	0.00%	0.00%	0.05%	0.11%	0.00%	0.00%
DP4+ (C data)	3.12%	0.00%	0.00%	0.00%	0.00%	0.09%	0.00%	96.78%
DP4+ (all data)	0.00%	61.05%	0.00%	0.00%	0.00%	10.15%	0.00%	28.80%

Figure S22. DP4+ probability of compounds **2** and **3** ((-)-2-*epi*-pseudolycorine and (+)-2-*epi*-pseudolycorine)

5. Contributing conformers and Boltzmann-weighted population

5.1. Compound **1**

5.1.1. SRR configuration

Conformer	Population (%)
SRR_c0.log	74.184
SRR-inv-N_c1.log	7.777
SRR-inv-N_c0.log	6.251
SRR_c1.log	5.046
SRR-inv-N_c2.log	4.690
SRR-inv-N_c5.log	2.053

Cartesian coordinate of SRR_c0:

Center Number	Atomic Number	Atomic Type	Coordinates (Angstroms) X	Y	Z
1	7	0	-1.399861	-2.565134	2.569253
2	6	0	0.060383	-2.508195	2.655650
3	6	0	0.709260	-1.135607	2.586680
4	6	0	0.357161	-0.195069	1.612621
5	6	0	-0.588897	-0.254775	0.416267
6	6	0	-1.972143	-0.868004	0.720081
7	6	0	-1.991506	-2.315186	1.257843
8	8	0	0.550816	1.858438	0.552081
9	6	0	0.990755	1.040355	1.568473
10	6	0	-0.722267	1.278383	0.132029
11	6	0	1.698596	-0.754621	3.508329
12	6	0	2.329565	0.492344	3.456476
13	6	0	1.979114	1.421060	2.473491
14	6	0	0.136924	-0.965244	-0.768876
15	6	0	-0.392485	-0.559880	-2.151982
16	6	0	-0.252239	0.951225	-2.358718
17	6	0	-1.045828	1.701984	-1.293045
18	8	0	2.579163	2.647791	2.418537
19	8	0	-0.769394	1.379593	-3.617391
20	1	0	-1.809188	-1.941043	3.262737
21	1	0	2.157608	3.146006	1.698145
22	1	0	-0.255664	0.937618	-4.311367
23	1	0	0.448150	-3.151150	1.851981
24	1	0	0.360344	-2.989801	3.595086
25	1	0	-2.590778	-0.836301	-0.188736
26	1	0	-2.480388	-0.228149	1.455631

27	1	0	-3.041885	-2.632337	1.305248
28	1	0	-1.505444	-2.995826	0.546929
29	1	0	-1.482208	1.688728	0.813574
30	1	0	1.984402	-1.452289	4.292212
31	1	0	3.085764	0.766799	4.185718
32	1	0	0.078969	-2.051524	-0.640741
33	1	0	1.203261	-0.712729	-0.719671
34	1	0	-1.448362	-0.833936	-2.278721
35	1	0	0.168359	-1.098338	-2.929219
36	1	0	0.812548	1.224102	-2.271950
37	1	0	-0.892130	2.781966	-1.395346
38	1	0	-2.112409	1.515763	-1.476013

Cartesian coordinate of SRR-inv-N_c1:

Center Number	Atomic Number	Atomic Type	Coordinates (Angstroms) X	Y	Z
1	7	0	-1.300395	-2.605939	2.361123
2	6	0	0.162506	-2.552463	2.433330
3	6	0	0.800249	-1.173034	2.407927
4	6	0	0.483125	-0.233079	1.419086
5	6	0	-0.430698	-0.322384	0.204332
6	6	0	-1.838701	-0.850815	0.553326
7	6	0	-1.910537	-2.305646	1.067004
8	8	0	0.641898	1.855850	0.424517
9	6	0	1.050224	1.030717	1.443920
10	6	0	-0.497683	1.192549	-0.210708
11	6	0	1.708988	-0.770636	3.398594
12	6	0	2.286523	0.504401	3.408700
13	6	0	1.957406	1.436421	2.423226

14	6	0	0.214166	-1.159586	-0.940543
15	6	0	1.328533	-0.385721	-1.652064
16	6	0	0.782866	0.846006	-2.403865
17	6	0	-0.452042	1.446883	-1.707418
18	8	0	2.499612	2.691436	2.430563
19	8	0	0.337836	0.511559	-3.724194
20	1	0	-1.697119	-2.002419	3.079841
21	1	0	2.105019	3.183317	1.690949
22	1	0	1.114943	0.230223	-4.232155
23	1	0	0.541754	-3.168841	1.605666
24	1	0	0.470860	-3.063305	3.354228
25	1	0	-2.475914	-0.772424	-0.341089
26	1	0	-2.284990	-0.192393	1.312282
27	1	0	-2.970896	-2.581627	1.136821
28	1	0	-1.469283	-2.988402	0.329694
29	1	0	-1.399975	1.647763	0.219646
30	1	0	1.970180	-1.469161	4.190223
31	1	0	2.982063	0.796517	4.189630
32	1	0	-0.558194	-1.425308	-1.675518
33	1	0	0.604329	-2.100662	-0.539865
34	1	0	1.848041	-1.035335	-2.368880
35	1	0	2.079902	-0.066952	-0.919590
36	1	0	1.576006	1.605230	-2.464278
37	1	0	-0.525065	2.520320	-1.910482
38	1	0	-1.339685	0.981662	-2.154299

Cartesian coordinate of SRR-inv-N_c0:

Center Number	Atomic Number	Atomic Type	Coordinates (Angstroms) X	Y	Z

1	7	0	-1.424209	-2.304417	2.577358
2	6	0	0.042572	-2.352135	2.651132
3	6	0	0.695958	-0.987911	2.581007
4	6	0	0.349240	-0.047231	1.607917
5	6	0	-0.596382	-0.100832	0.411433
6	6	0	-1.974126	-0.731153	0.704778
7	6	0	-1.972830	-2.176034	1.225400
8	8	0	0.535484	2.012476	0.559071
9	6	0	0.984365	1.187658	1.566580
10	6	0	-0.740775	1.433508	0.145882
11	6	0	1.695792	-0.617134	3.494424
12	6	0	2.334565	0.625681	3.440657
13	6	0	1.980128	1.560941	2.465549
14	6	0	0.138781	-0.786637	-0.783069
15	6	0	-0.397711	-0.371806	-2.160727
16	6	0	-0.279615	1.143382	-2.350105
17	6	0	-1.077868	1.870730	-1.271806
18	8	0	2.584580	2.786114	2.412070
19	8	0	-0.808255	1.578921	-3.601929
20	1	0	-1.784484	-3.164279	2.983829
21	1	0	2.154462	3.291133	1.701590
22	1	0	-0.291354	1.151535	-4.302650
23	1	0	0.465419	-3.005953	1.860538
24	1	0	0.309745	-2.814241	3.607703
25	1	0	-2.582127	-0.703256	-0.210282
26	1	0	-2.490013	-0.116128	1.451902
27	1	0	-3.016955	-2.508174	1.266764
28	1	0	-1.462279	-2.842332	0.502754
29	1	0	-1.495611	1.832617	0.839355
30	1	0	1.980857	-1.315817	4.277398
31	1	0	3.097245	0.892763	4.165771
32	1	0	0.095839	-1.875009	-0.668066

33	1	0	1.201474	-0.519659	-0.731419
34	1	0	-1.449796	-0.659188	-2.289710
35	1	0	0.169584	-0.892858	-2.945272
36	1	0	0.781578	1.430122	-2.265049
37	1	0	-0.938458	2.953772	-1.361843
38	1	0	-2.143046	1.673119	-1.451267

Cartesian coordinate of SRR_c1:

Center Number	Atomic Number	Atomic Type	Coordinates (Angstroms) X	Y	Z
1	7	0	-1.418491	-2.305006	2.316442
2	6	0	0.048497	-2.352667	2.387537
3	6	0	0.700914	-0.987791	2.320770
4	6	0	0.352767	-0.044836	1.350434
5	6	0	-0.593596	-0.096992	0.154383
6	6	0	-1.971215	-0.727882	0.447872
7	6	0	-1.969122	-2.173849	0.965407
8	8	0	0.535274	2.018801	0.307742
9	6	0	0.986434	1.190894	1.313005
10	6	0	-0.739089	1.437662	-0.106120
11	6	0	1.700717	-0.618552	3.234863
12	6	0	2.337998	0.625079	3.184729
13	6	0	1.981928	1.562787	2.212584
14	6	0	0.139575	-0.780637	-1.043187
15	6	0	-0.392672	-0.366888	-2.422317
16	6	0	-0.270291	1.141717	-2.609699
17	6	0	-1.072836	1.871772	-1.526777
18	8	0	2.584875	2.789180	2.163187
19	8	0	-0.758992	1.456152	-3.913002

20	1	0	-1.777668	-3.166183	2.721127
21	1	0	2.154552	3.295414	1.453883
22	1	0	-0.643356	2.409623	-4.049767
23	1	0	0.470399	-3.003481	1.594145
24	1	0	0.317514	-2.817652	3.342189
25	1	0	-2.579135	-0.698907	-0.467181
26	1	0	-2.487037	-0.114592	1.196581
27	1	0	-3.013198	-2.505991	1.007616
28	1	0	-1.459723	-2.838368	0.240616
29	1	0	-1.496010	1.834957	0.586360
30	1	0	1.986982	-1.319341	4.015494
31	1	0	3.100762	0.890853	3.910219
32	1	0	0.092509	-1.868990	-0.930039
33	1	0	1.203265	-0.518311	-0.989951
34	1	0	-1.445375	-0.652273	-2.551765
35	1	0	0.170986	-0.876859	-3.212071
36	1	0	0.792198	1.422231	-2.524793
37	1	0	-0.935557	2.959193	-1.603287
38	1	0	-2.137212	1.672640	-1.708709

Cartesian coordinate of SRR-inv-N_c2:

Center Number	Atomic Number	Atomic Type	Coordinates (Angstroms) X	Y	Z
1	7	0	-1.289899	-2.605506	2.355378
2	6	0	0.173855	-2.551827	2.412823
3	6	0	0.809158	-1.171427	2.385004
4	6	0	0.483029	-0.230763	1.399763
5	6	0	-0.439280	-0.321468	0.191745
6	6	0	-1.844731	-0.848678	0.553889

7	6	0	-1.913363	-2.303623	1.068053
8	8	0	0.623672	1.863079	0.411675
9	6	0	1.044208	1.035459	1.425043
10	6	0	-0.510014	1.192892	-0.224377
11	6	0	1.722348	-0.767732	3.371000
12	6	0	2.294951	0.509448	3.380464
13	6	0	1.955686	1.442673	2.399577
14	6	0	0.193674	-1.160122	-0.958971
15	6	0	1.320896	-0.407317	-1.674379
16	6	0	0.822781	0.860178	-2.388715
17	6	0	-0.449285	1.433214	-1.724311
18	8	0	2.492319	2.700115	2.407857
19	8	0	0.552155	0.497939	-3.748453
20	1	0	-1.679253	-2.003070	3.079020
21	1	0	2.095790	3.191008	1.668764
22	1	0	0.245544	1.294268	-4.210471
23	1	0	0.545311	-3.165546	1.579841
24	1	0	0.491611	-3.064724	3.329327
25	1	0	-2.490115	-0.770211	-0.334849
26	1	0	-2.283690	-0.189570	1.316571
27	1	0	-2.973400	-2.577762	1.149306
28	1	0	-1.481364	-2.986715	0.325745
29	1	0	-1.416959	1.646966	0.197401
30	1	0	1.991169	-1.467065	4.159326
31	1	0	2.994570	0.802384	4.157411
32	1	0	-0.586597	-1.419457	-1.687822
33	1	0	0.575010	-2.106407	-0.562047
34	1	0	1.797356	-1.046695	-2.424851
35	1	0	2.093996	-0.135096	-0.946876
36	1	0	1.618343	1.618133	-2.363731
37	1	0	-0.550134	2.505453	-1.934517
38	1	0	-1.317045	0.934862	-2.175892

Cartesian coordinate of SRR-inv-N_c5:

Center Number	Atomic Number	Atomic Type	Coordinates (Angstroms) X	Y	Z
1	7	0	-1.291626	-2.603441	2.211041
2	6	0	0.172398	-2.550859	2.264063
3	6	0	0.808848	-1.171316	2.226939
4	6	0	0.482155	-0.235851	1.236831
5	6	0	-0.443658	-0.331938	0.032003
6	6	0	-1.848603	-0.855443	0.401444
7	6	0	-1.918847	-2.307287	0.924662
8	8	0	0.622131	1.852135	0.236675
9	6	0	1.043484	1.030483	1.253765
10	6	0	-0.513095	1.180532	-0.394647
11	6	0	1.723598	-0.762373	3.209183
12	6	0	2.296768	0.514607	3.210267
13	6	0	1.956791	1.442765	2.224780
14	6	0	0.186552	-1.179301	-1.113858
15	6	0	1.317774	-0.431842	-1.832011
16	6	0	0.830042	0.849485	-2.547938
17	6	0	-0.451178	1.413640	-1.895726
18	8	0	2.494271	2.699170	2.225694
19	8	0	0.631165	0.656872	-3.950444
20	1	0	-1.678695	-1.998193	2.933507
21	1	0	2.100120	3.186870	1.483134
22	1	0	-0.051415	-0.025163	-4.059087
23	1	0	0.540858	-3.170134	1.433721
24	1	0	0.491933	-3.059020	3.182487
25	1	0	-2.497982	-0.780279	-0.485019

26	1	0	-2.283418	-0.190764	1.161337
27	1	0	-2.979308	-2.578087	1.011178
28	1	0	-1.491373	-2.996378	0.184978
29	1	0	-1.418867	1.637336	0.026510
30	1	0	1.993225	-1.457124	4.001240
31	1	0	2.997786	0.811524	3.984410
32	1	0	-0.603060	-1.444900	-1.833771
33	1	0	0.562054	-2.126885	-0.714719
34	1	0	1.800496	-1.073638	-2.577823
35	1	0	2.089537	-0.171004	-1.098213
36	1	0	1.619499	1.603606	-2.498773
37	1	0	-0.556097	2.478979	-2.125879
38	1	0	-1.325570	0.915315	-2.339799

5.1.2. SRS configuration

Conformer	Population (%)
SRS-inv-N_c0.log	48.199
SRS_c0.log	40.463
SRS-inv-N_c3.log	8.247
SRS_c6.log	1.577
SRS_c7.log	1.513

Cartesian coordinate of SRS-inv-N_c0:

Center Number	Atomic Number	Atomic Type	Coordinates (Angstroms) X	Y	Z
1	7	0	-0.702554	-2.716359	-2.122995
2	6	0	0.770440	-2.674757	-2.172659
3	6	0	1.315553	-1.260755	-2.157381
4	6	0	1.057544	-0.447654	-1.051054
5	6	0	0.328559	-0.743125	0.252516

6	6	0	-1.146289	-1.044797	-0.143897
7	6	0	-1.350389	-2.421714	-0.841186
8	8	0	0.649378	1.629846	-0.079556
9	6	0	1.162577	0.935406	-1.159445
10	6	0	0.605856	0.617443	0.956823
11	6	0	1.847568	-0.641958	-3.298346
12	6	0	2.054863	0.742105	-3.359626
13	6	0	1.681173	1.562411	-2.292247
14	6	0	0.865300	-1.780749	1.269247
15	6	0	0.112971	-1.630298	2.616724
16	6	0	0.080814	-0.202971	3.200543
17	6	0	-0.280651	0.884870	2.150531
18	8	0	1.803642	2.921648	-2.375046
19	8	0	1.384757	0.033029	3.742563
20	1	0	-1.065720	-2.089808	-2.839813
21	1	0	1.512591	3.291450	-1.524627
22	1	0	1.389065	0.919973	4.135865
23	1	0	1.087754	-3.186172	-3.089621
24	1	0	1.146339	-3.271561	-1.332781
25	1	0	-1.507677	-0.242501	-0.799958
26	1	0	-1.804259	-1.042445	0.734586
27	1	0	-1.065508	-3.221128	-0.145488
28	1	0	-2.428267	-2.544847	-1.006417
29	1	0	1.635805	0.553697	1.333057
30	1	0	2.080045	-1.246706	-4.172120
31	1	0	2.470214	1.205750	-4.249330
32	1	0	1.937274	-1.617483	1.424300
33	1	0	0.755227	-2.810649	0.917525
34	1	0	0.574739	-2.278088	3.369925
35	1	0	-0.921264	-1.975397	2.501696
36	1	0	-0.664488	-0.176992	4.010816
37	1	0	-1.345100	0.840156	1.894053

38	1	0	-0.091518	1.880396	2.574301

Cartesian coordinate of SRS_c0:

Center Number	Atomic Number	Atomic Type	Coordinates (Angstroms) X	Y	Z
1	7	0	-0.703733	-2.715930	-2.243917
2	6	0	0.769524	-2.675985	-2.292782
3	6	0	1.316627	-1.262827	-2.276173
4	6	0	1.059459	-0.450521	-1.169026
5	6	0	0.328959	-0.744900	0.133702
6	6	0	-1.145921	-1.043239	-0.265191
7	6	0	-1.352167	-2.419640	-0.962664
8	8	0	0.648564	1.625818	-0.197386
9	6	0	1.162608	0.932885	-1.276321
10	6	0	0.609188	0.614360	0.840526
11	6	0	1.847998	-0.643141	-3.416920
12	6	0	2.055387	0.741069	-3.476725
13	6	0	1.681804	1.560642	-2.408674
14	6	0	0.861878	-1.785454	1.148802
15	6	0	0.099480	-1.634395	2.490037
16	6	0	0.072787	-0.203175	3.079589
17	6	0	-0.274625	0.885799	2.036726
18	8	0	1.804299	2.919527	-2.489936
19	8	0	1.353137	0.166641	3.604253
20	1	0	-1.065214	-2.088430	-2.960787
21	1	0	1.518944	3.288431	-1.637010
22	1	0	1.623770	-0.518040	4.235527
23	1	0	1.086462	-3.187367	-3.209910
24	1	0	1.143956	-3.274434	-1.453260

25	1	0	-1.502866	-0.239995	-0.922320
26	1	0	-1.806718	-1.037233	0.611155
27	1	0	-1.069837	-3.220077	-0.266781
28	1	0	-2.430121	-2.540812	-1.128956
29	1	0	1.640363	0.552357	1.211785
30	1	0	2.079822	-1.247014	-4.291494
31	1	0	2.470806	1.205520	-4.365985
32	1	0	1.932789	-1.618268	1.310084
33	1	0	0.757375	-2.815025	0.794430
34	1	0	0.540135	-2.307510	3.239547
35	1	0	-0.935950	-1.974871	2.365662
36	1	0	-0.676804	-0.173959	3.886175
37	1	0	-1.338836	0.849510	1.780648
38	1	0	-0.063414	1.865800	2.476678

Cartesian coordinate of SRS-inv-N_c3:

Center Number	Atomic Number	Atomic Type	Coordinates (Angstroms) X	Y	Z
1	7	0	-0.708951	-2.720504	-1.960208
2	6	0	0.763789	-2.680564	-2.006594
3	6	0	1.310899	-1.267093	-1.991738
4	6	0	1.049368	-0.451090	-0.888369
5	6	0	0.315102	-0.742616	0.412790
6	6	0	-1.158755	-1.044150	0.013204
7	6	0	-1.361034	-2.422627	-0.681609
8	8	0	0.636234	1.627405	0.078423
9	6	0	1.154450	0.931809	-0.999198
10	6	0	0.586745	0.620773	1.115360
11	6	0	1.847507	-0.650175	-3.131573

12	6	0	2.054959	0.733692	-3.194816
13	6	0	1.676888	1.557207	-2.131094
14	6	0	0.846829	-1.780802	1.431338
15	6	0	0.087558	-1.630194	2.777085
16	6	0	0.055611	-0.198892	3.367393
17	6	0	-0.292319	0.892483	2.312951
18	8	0	1.799708	2.915228	-2.218157
19	8	0	1.276617	0.118477	4.042260
20	1	0	-1.070920	-2.097967	-2.680991
21	1	0	1.502863	3.291105	-1.372343
22	1	0	2.016686	-0.073687	3.446256
23	1	0	1.082888	-3.193380	-2.922074
24	1	0	1.137169	-3.277496	-1.165511
25	1	0	-1.515519	-0.243004	-0.646413
26	1	0	-1.819464	-1.036872	0.889161
27	1	0	-1.080246	-3.221187	0.016960
28	1	0	-2.438366	-2.544802	-0.850184
29	1	0	1.626867	0.558785	1.476782
30	1	0	2.083186	-1.256383	-4.003418
31	1	0	2.473304	1.195426	-4.084062
32	1	0	1.924348	-1.623830	1.576134
33	1	0	0.742424	-2.810902	1.079660
34	1	0	0.532948	-2.288053	3.532288
35	1	0	-0.944884	-1.974161	2.642312
36	1	0	-0.697087	-0.167986	4.161604
37	1	0	-1.355118	0.855641	2.048100
38	1	0	-0.085395	1.874247	2.750600

Cartesian coordinate of SRS_c6:

Center Atomic Atomic Coordinates (Angstroms)

Number	Number	Type	X	Y	Z
1	7	0	-1.124946	-2.434750	-1.673689
2	6	0	0.349492	-2.515438	-1.645237
3	6	0	1.002050	-1.149349	-1.693447
4	6	0	0.366555	-0.019405	-1.171857
5	6	0	-1.008955	0.172777	-0.545414
6	6	0	-2.031151	-0.079990	-1.694390
7	6	0	-1.713106	-1.371847	-2.503479
8	8	0	0.016757	2.283579	-1.084514
9	6	0	0.849241	1.253525	-1.475454
10	6	0	-0.830389	1.647231	-0.093258
11	6	0	2.209290	-0.945184	-2.381273
12	6	0	2.740049	0.331952	-2.587468
13	6	0	2.045060	1.465339	-2.156739
14	6	0	-1.423436	-0.563925	0.751273
15	6	0	-2.708520	0.077852	1.334582
16	6	0	-2.680816	1.615317	1.500017
17	6	0	-2.124225	2.347389	0.257117
18	8	0	2.515562	2.721261	-2.420561
19	8	0	-1.839479	2.013573	2.589210
20	1	0	-1.497479	-3.339147	-1.943851
21	1	0	1.900513	3.351943	-2.009185
22	1	0	-2.111633	1.508723	3.371151
23	1	0	0.748611	-3.100213	-2.492539
24	1	0	0.633260	-3.061589	-0.732757
25	1	0	-2.025801	0.770423	-2.388496
26	1	0	-3.047467	-0.150234	-1.292664
27	1	0	-2.650104	-1.753328	-2.924584
28	1	0	-1.069405	-1.118791	-3.359029
29	1	0	-0.223472	1.615164	0.820777
30	1	0	2.738533	-1.802169	-2.791396

31	1	0	3.676445	0.466995	-3.120206
32	1	0	-0.610882	-0.489970	1.484564
33	1	0	-1.599299	-1.622822	0.550902
34	1	0	-2.927577	-0.372090	2.314124
35	1	0	-3.568446	-0.177141	0.702573
36	1	0	-3.708989	1.964739	1.686561
37	1	0	-2.847583	2.309663	-0.564986
38	1	0	-1.955565	3.397843	0.516046

Cartesian coordinate of SRS_c7:

Center Number	Atomic Number	Atomic Type	Coordinates (Angstroms) X	Y	Z
1	7	0	-1.123367	-2.431373	-1.375453
2	6	0	0.350511	-2.511876	-1.350497
3	6	0	1.002766	-1.145575	-1.398275
4	6	0	0.368160	-0.015883	-0.875130
5	6	0	-1.006120	0.174881	-0.244996
6	6	0	-2.032493	-0.078488	-1.390139
7	6	0	-1.714890	-1.368666	-2.202082
8	8	0	0.021559	2.287972	-0.782437
9	6	0	0.852925	1.256641	-1.176798
10	6	0	-0.827302	1.649340	0.205219
11	6	0	2.209804	-0.941448	-2.086593
12	6	0	2.741480	0.335251	-2.292175
13	6	0	2.047639	1.468576	-1.859490
14	6	0	-1.416504	-0.560325	1.054412
15	6	0	-2.696522	0.080702	1.650598
16	6	0	-2.671127	1.613462	1.810803
17	6	0	-2.122725	2.344584	0.554825

18	8	0	2.519112	2.724714	-2.122901
19	8	0	-1.853308	1.887851	2.954042
20	1	0	-1.497786	-3.336084	-1.641509
21	1	0	1.901497	3.355423	-1.715819
22	1	0	-1.830187	2.849714	3.079509
23	1	0	0.748323	-3.095306	-2.199481
24	1	0	0.636786	-3.058574	-0.439234
25	1	0	-2.033385	0.772146	-2.084242
26	1	0	-3.045992	-0.152587	-0.982069
27	1	0	-2.652608	-1.750410	-2.621303
28	1	0	-1.073845	-1.113323	-3.059075
29	1	0	-0.223410	1.615590	1.122032
30	1	0	2.738095	-1.798511	-2.497732
31	1	0	3.677351	0.470133	-2.825857
32	1	0	-0.600306	-0.492154	1.783414
33	1	0	-1.598156	-1.617925	0.852598
34	1	0	-2.892545	-0.347457	2.640030
35	1	0	-3.562263	-0.175547	1.028185
36	1	0	-3.698446	1.960658	2.003988
37	1	0	-2.848481	2.299746	-0.265626
38	1	0	-1.959443	3.406009	0.786414

5.1.3. SSR configuration

Conformer	Population (%)
ssr_c1.log	60.697
ssr_c2.log	15.489
ssr_c3.log	15.229
ssr_c5.log	2.885
ssr_c4.log	2.806
ssr_c7.log	1.771
ssr_c18.log	1.122

Cartesian coordinate of SSR_c1:

Center Number	Atomic Number	Atomic Type	Coordinates (Angstroms) X	Y	Z
1	6	0	-2.199831	-1.989568	2.001952
2	6	0	-2.753852	-1.551792	0.794109
3	6	0	-2.040831	-0.749648	-0.115448
4	6	0	-0.740457	-0.383791	0.249210
5	6	0	-0.200978	-0.825071	1.453294
6	6	0	-0.896137	-1.627522	2.353100
7	6	0	-2.662608	-0.323743	-1.435993
8	7	0	-2.530826	1.082821	-1.816908
9	6	0	-1.212331	1.533882	-2.253645
10	6	0	-0.111107	1.678421	-1.181262
11	6	0	0.357883	0.384938	-0.481714
12	6	0	1.267055	0.733023	0.740164
13	8	0	1.097308	-0.397721	1.665628
14	6	0	1.098737	-0.592429	-1.450558
15	6	0	2.554664	-0.205025	-1.744941
16	6	0	3.380813	-0.076569	-0.454913
17	6	0	2.747032	0.967589	0.472317
18	8	0	-0.352846	-2.046964	3.537046
19	8	0	3.543022	-1.339941	0.199923
20	1	0	0.838851	1.604885	1.254805
21	1	0	-2.774715	-2.601475	2.689720
22	1	0	-3.773853	-1.841000	0.553965
23	1	0	-2.235955	-0.916414	-2.257454
24	1	0	-3.731076	-0.567926	-1.412758
25	1	0	-2.897840	1.686062	-1.085055
26	1	0	-1.340594	2.515390	-2.727049
27	1	0	-0.883651	0.856922	-3.051972
28	1	0	0.754090	2.165716	-1.653380

29	1	0	-0.475901	2.376449	-0.414921
30	1	0	0.533801	-0.679252	-2.384048
31	1	0	1.093629	-1.594117	-1.007582
32	1	0	2.606917	0.746062	-2.292958
33	1	0	3.012153	-0.966995	-2.385178
34	1	0	4.397023	0.243985	-0.710504
35	1	0	2.846209	1.958474	0.010831
36	1	0	3.285778	1.007261	1.426003
37	1	0	0.526899	-1.654726	3.635797
38	1	0	2.785527	-1.483190	0.786578

Cartesian coordinate of SSR_c2:

Center Number	Atomic Number	Atomic Type	Coordinates (Angstroms) X	Y	Z
1	6	0	-2.599112	0.436956	0.053025
2	6	0	-1.788916	1.564880	0.234500
3	6	0	-0.422902	1.463430	0.547172
4	6	0	0.105024	0.171704	0.671187
5	6	0	-0.708022	-0.938856	0.498503
6	6	0	-2.064823	-0.845640	0.191084
7	6	0	0.429939	2.702325	0.770712
8	7	0	1.189488	2.762297	2.022197
9	6	0	2.357691	1.891301	2.140254
10	6	0	2.113704	0.369265	2.236017
11	6	0	1.517150	-0.307374	0.981814
12	6	0	1.250417	-1.830438	1.258751
13	8	0	-0.057035	-2.142333	0.656359
14	6	0	2.463917	-0.150183	-0.246367
15	6	0	2.181173	-1.189511	-1.340430

16	6	0	2.416774	-2.642611	-0.861127
17	6	0	2.295487	-2.763721	0.673807
18	8	0	-2.857383	-1.950930	0.031629
19	8	0	1.545975	-3.560396	-1.528443
20	1	0	1.134867	-1.995916	2.337520
21	1	0	-3.654578	0.540271	-0.177813
22	1	0	-2.237612	2.550398	0.139603
23	1	0	1.153732	2.821585	-0.047234
24	1	0	-0.219178	3.584494	0.729683
25	1	0	0.567371	2.640110	2.817405
26	1	0	2.901567	2.199072	3.041677
27	1	0	3.022277	2.115495	1.296614
28	1	0	3.074538	-0.113565	2.471031
29	1	0	1.449519	0.177180	3.089740
30	1	0	2.365820	0.856677	-0.662768
31	1	0	3.505534	-0.242205	0.092891
32	1	0	2.791289	-0.987913	-2.226935
33	1	0	1.137455	-1.094788	-1.663109
34	1	0	3.420043	-2.969581	-1.152770
35	1	0	3.255285	-2.519779	1.147863
36	1	0	2.073822	-3.804668	0.931115
37	1	0	-2.342646	-2.741083	0.250725
38	1	0	0.643951	-3.361046	-1.236884

Cartesian coordinate of SSR_c3:

Center Number	Atomic Number	Atomic Type	Coordinates (Angstroms) X	Y	Z
1	6	0	-2.316827	-2.180534	1.893509
2	6	0	-2.847331	-1.715998	0.685311

3	6	0	-2.096184	-0.945862	-0.220045
4	6	0	-0.779887	-0.648345	0.142880
5	6	0	-0.266008	-1.109622	1.350804
6	6	0	-0.999737	-1.877040	2.249567
7	6	0	-2.694760	-0.488657	-1.533904
8	7	0	-2.455706	0.930684	-1.828050
9	6	0	-1.136782	1.247032	-2.382413
10	6	0	-0.040123	1.361532	-1.313462
11	6	0	0.358984	0.058619	-0.588095
12	6	0	1.271327	0.380627	0.637387
13	8	0	1.048506	-0.736758	1.568382
14	6	0	1.064534	-0.968266	-1.532206
15	6	0	2.538400	-0.649817	-1.820559
16	6	0	3.359591	-0.533078	-0.526246
17	6	0	2.761647	0.550923	0.379015
18	8	0	-0.480493	-2.318531	3.436810
19	8	0	3.465495	-1.790797	0.150677
20	1	0	0.874669	1.272914	1.141733
21	1	0	-2.922080	-2.764286	2.579571
22	1	0	-3.880728	-1.953239	0.446406
23	1	0	-2.317936	-1.134535	-2.351937
24	1	0	-3.777659	-0.640973	-1.490908
25	1	0	-3.170353	1.253367	-2.472373
26	1	0	-1.216227	2.222015	-2.875795
27	1	0	-0.835225	0.523685	-3.163286
28	1	0	0.852269	1.786252	-1.793178
29	1	0	-0.383744	2.089457	-0.568947
30	1	0	0.504039	-1.047456	-2.468990
31	1	0	1.011822	-1.960443	-1.071268
32	1	0	2.636110	0.287806	-2.385394
33	1	0	2.967763	-1.442508	-2.442837
34	1	0	4.389964	-0.258396	-0.778753

35	1	0	2.904672	1.529781	-0.096573
36	1	0	3.294145	0.583506	1.336494
37	1	0	0.413075	-1.960499	3.540872
38	1	0	2.695478	-1.894063	0.729604

Cartesian coordinate of SSR_c5:

Center Number	Atomic Number	Atomic Type	Coordinates (Angstroms) X	Y	Z
1	6	0	0.138782	2.565245	2.691720
2	6	0	1.274699	2.471357	1.877962
3	6	0	1.381372	1.522585	0.845813
4	6	0	0.302305	0.647307	0.676361
5	6	0	-0.816713	0.741428	1.496419
6	6	0	-0.936165	1.692469	2.507963
7	6	0	2.613594	1.464871	-0.042250
8	7	0	3.218522	0.148958	-0.260493
9	6	0	2.504502	-0.774037	-1.139403
10	6	0	1.192848	-1.398418	-0.615712
11	6	0	0.013018	-0.434557	-0.359454
12	6	0	-1.150044	-1.181824	0.374018
13	8	0	-1.797630	-0.175567	1.210645
14	6	0	-0.514976	0.235478	-1.664211
15	6	0	-1.344464	-0.708063	-2.545069
16	6	0	-2.578462	-1.231931	-1.796056
17	6	0	-2.172823	-1.916595	-0.487751
18	8	0	-2.047149	1.773510	3.303686
19	8	0	-3.494240	-0.176953	-1.474291
20	1	0	-0.701828	-1.901998	1.074870
21	1	0	0.080871	3.305383	3.483602

22	1	0	2.100472	3.156385	2.053113
23	1	0	2.381703	1.875262	-1.035237
24	1	0	3.381457	2.121717	0.382957
25	1	0	3.431830	-0.293231	0.630285
26	1	0	3.189830	-1.597964	-1.375329
27	1	0	2.328139	-0.250836	-2.087634
28	1	0	0.881985	-2.173140	-1.331603
29	1	0	1.419180	-1.923420	0.322436
30	1	0	-1.146184	1.084121	-1.382866
31	1	0	0.323763	0.647465	-2.234203
32	1	0	-0.747387	-1.565496	-2.884031
33	1	0	-1.663370	-0.181143	-3.454598
34	1	0	-3.094248	-1.977223	-2.420982
35	1	0	-3.065339	-2.119729	0.113361
36	1	0	-1.737217	-2.890165	-0.746752
37	1	0	-2.679725	1.096502	3.021331
38	1	0	-3.811307	0.221147	-2.295916

Cartesian coordinate of SSR_c4:

Center Number	Atomic Number	Atomic Type	Coordinates (Angstroms) X	Y	Z
1	6	0	-0.854200	-0.197437	-2.728096
2	6	0	0.366764	0.481486	-2.631370
3	6	0	0.976756	0.749926	-1.395582
4	6	0	0.312949	0.300263	-0.249035
5	6	0	-0.903718	-0.357695	-0.354393
6	6	0	-1.518958	-0.623062	-1.576439
7	6	0	2.283340	1.511739	-1.311453
8	7	0	2.215501	2.675750	-0.415275

9	6	0	2.359275	2.390480	1.015732
10	6	0	1.082633	1.822221	1.654127
11	6	0	0.685576	0.391790	1.224516
12	6	0	-0.675571	-0.018295	1.890944
13	8	0	-1.450086	-0.724312	0.856115
14	6	0	1.791802	-0.639865	1.603175
15	6	0	1.255922	-2.076929	1.672024
16	6	0	0.173859	-2.260506	2.763602
17	6	0	-0.530183	-0.927126	3.098549
18	8	0	-2.723778	-1.268767	-1.666004
19	8	0	-0.769277	-3.272271	2.398801
20	1	0	-1.251142	0.881955	2.141192
21	1	0	-1.313047	-0.382451	-3.694036
22	1	0	0.845792	0.824192	-3.544665
23	1	0	3.099659	0.823907	-1.014838
24	1	0	2.533914	1.878621	-2.311421
25	1	0	2.930425	3.341528	-0.690937
26	1	0	2.581782	3.341586	1.511772
27	1	0	3.214417	1.719568	1.221359
28	1	0	1.218906	1.829101	2.745397
29	1	0	0.258017	2.506407	1.422386
30	1	0	2.609619	-0.587415	0.878538
31	1	0	2.223965	-0.358414	2.574053
32	1	0	2.075501	-2.783332	1.840053
33	1	0	0.820042	-2.343736	0.701937
34	1	0	0.634331	-2.645228	3.679353
35	1	0	0.043953	-0.377619	3.856137
36	1	0	-1.507748	-1.143921	3.541611
37	1	0	-3.077160	-1.398503	-0.774104
38	1	0	-1.259831	-2.940436	1.632170

Cartesian coordinate of SSR_c7:

Center Number	Atomic Number	Atomic Type	Coordinates (Angstroms) X	Y	Z
1	6	0	3.295972	0.244938	0.418485
2	6	0	2.935780	-0.304831	-0.819306
3	6	0	1.595019	-0.511208	-1.184291
4	6	0	0.619606	-0.150416	-0.245308
5	6	0	0.986436	0.372457	0.985933
6	6	0	2.315408	0.593116	1.349012
7	6	0	1.227007	-1.123733	-2.525950
8	7	0	0.322124	-2.276508	-2.500138
9	6	0	-1.083359	-2.018194	-2.190689
10	6	0	-1.436086	-1.582791	-0.751435
11	6	0	-0.901758	-0.203476	-0.306740
12	6	0	-1.256375	0.066619	1.201752
13	8	0	-0.067931	0.663436	1.811845
14	6	0	-1.469340	0.935718	-1.206977
15	6	0	-1.455567	2.294991	-0.493937
16	6	0	-2.445393	2.306938	0.682223
17	6	0	-2.461691	0.964152	1.442101
18	8	0	2.658749	1.118493	2.565164
19	8	0	-2.139874	3.334737	1.634251
20	1	0	-1.409801	-0.893412	1.712812
21	1	0	4.339134	0.393400	0.679136
22	1	0	3.722506	-0.584088	-1.515588
23	1	0	2.148323	-1.432326	-3.033265
24	1	0	0.761933	-0.370309	-3.176965
25	1	0	0.695869	-3.002020	-1.893151
26	1	0	-1.447052	-1.274247	-2.910668
27	1	0	-1.637393	-2.940330	-2.405339

28	1	0	-2.532825	-1.582392	-0.656976
29	1	0	-1.061907	-2.346446	-0.056139
30	1	0	-0.894668	0.995560	-2.135603
31	1	0	-2.501140	0.687968	-1.496682
32	1	0	-1.706728	3.096297	-1.200255
33	1	0	-0.450740	2.506373	-0.111283
34	1	0	-3.458029	2.485456	0.287532
35	1	0	-2.558071	1.171629	2.512254
36	1	0	-3.350509	0.390323	1.151658
37	1	0	1.849230	1.297061	3.066364
38	1	0	-2.104158	4.184662	1.175061

Cartesian coordinate of SSR_c18:

Center Number	Atomic Number	Atomic Type	Coordinates (Angstroms) X	Y	Z
1	6	0	0.205937	0.217522	3.682374
2	6	0	0.016837	-1.088658	3.216578
3	6	0	-0.145968	-1.373153	1.851407
4	6	0	-0.140291	-0.283639	0.972191
5	6	0	0.040271	1.016376	1.444264
6	6	0	0.226205	1.301134	2.797432
7	6	0	-0.295100	-2.807922	1.369654
8	7	0	-1.360076	-3.088460	0.407162
9	6	0	-1.155882	-2.620915	-0.961616
10	6	0	-1.253263	-1.103019	-1.220118
11	6	0	-0.189635	-0.206109	-0.552320
12	6	0	-0.582076	1.296302	-0.702083
13	8	0	0.037868	1.962428	0.445954
14	6	0	1.249277	-0.452311	-1.112034

15	6	0	1.521355	0.216073	-2.466719
16	6	0	1.268964	1.731788	-2.412225
17	6	0	-0.178360	2.005587	-1.987285
18	8	0	0.405914	2.604451	3.176758
19	8	0	2.210212	2.391847	-1.558732
20	1	0	-1.667907	1.388390	-0.553186
21	1	0	0.327014	0.398949	4.748403
22	1	0	-0.001846	-1.903354	3.935850
23	1	0	0.644731	-3.144856	0.910187
24	1	0	-0.450714	-3.452618	2.242751
25	1	0	-2.257745	-2.774666	0.767799
26	1	0	-0.184762	-3.006197	-1.296416
27	1	0	-1.907107	-3.112291	-1.592607
28	1	0	-2.246975	-0.767762	-0.891750
29	1	0	-1.221089	-0.945837	-2.307854
30	1	0	1.975449	-0.063695	-0.390367
31	1	0	1.438396	-1.528739	-1.175264
32	1	0	0.892156	-0.218228	-3.256331
33	1	0	2.562990	0.039529	-2.756323
34	1	0	1.434017	2.157089	-3.409091
35	1	0	-0.344684	3.082582	-1.870219
36	1	0	-0.852350	1.664378	-2.783454
37	1	0	0.530184	2.648767	4.133873
38	1	0	1.840855	2.439535	-0.663744

5.1.4. SSS configuration

Conformer	Population (%)
SSS-inv-N_c0.log	47.949
SSS-inv-N_c1.log	45.667
SSS-inv-N_c5.log	2.210
SSS-inv-N_c7.log	2.208
SSS-inv-N_c6.log	1.967

Cartesian coordinate of SSS-inv-N_c0:

Center Number	Atomic Number	Atomic Type	Coordinates (Angstroms) X	Y	Z
1	7	0	-0.704583	-2.723089	-2.298785
2	6	0	0.768205	-2.680200	-2.345128
3	6	0	1.312165	-1.265571	-2.333956
4	6	0	1.049008	-0.446990	-1.232789
5	6	0	0.315386	-0.736958	0.069034
6	6	0	-1.157610	-1.044302	-0.327338
7	6	0	-1.356158	-2.424304	-1.019643
8	8	0	0.635183	1.634923	-0.272381
9	6	0	1.153822	0.935645	-1.347839
10	6	0	0.586525	0.628786	0.766590
11	6	0	1.848674	-0.651464	-3.475363
12	6	0	2.054993	0.732408	-3.542810
13	6	0	1.676158	1.558137	-2.481335
14	6	0	0.850664	-1.768152	1.093093
15	6	0	0.107348	-1.606186	2.443288
16	6	0	0.088109	-0.174322	3.012144
17	6	0	-0.298679	0.898609	1.960933
18	8	0	1.798291	2.916600	-2.571103
19	8	0	-0.815907	-0.194658	4.112542
20	1	0	-1.067345	-2.101160	-3.019777
21	1	0	1.496141	3.292951	-1.727479
22	1	0	-0.785435	0.676844	4.537629
23	1	0	1.088703	-3.194609	-3.259258
24	1	0	1.142696	-3.273530	-1.502063
25	1	0	-1.520100	-0.245198	-0.986631
26	1	0	-1.814198	-1.040567	0.551303

27	1	0	-1.072124	-3.220614	-0.320095
28	1	0	-2.433286	-2.550011	-1.186725
29	1	0	1.624209	0.563660	1.133877
30	1	0	2.085226	-1.260147	-4.345258
31	1	0	2.473103	1.191859	-4.433359
32	1	0	1.927895	-1.611996	1.232141
33	1	0	0.734340	-2.799689	0.749672
34	1	0	0.554842	-2.265767	3.195292
35	1	0	-0.935084	-1.928958	2.339150
36	1	0	1.104961	0.067486	3.367427
37	1	0	-1.363073	0.819804	1.717659
38	1	0	-0.119941	1.902781	2.367888

Cartesian coordinate of SSS-inv-N_c1:

Center Number	Atomic Number	Atomic Type	Coordinates (Angstroms) X	Y	Z
1	7	0	-0.700449	-2.724143	-2.126075
2	6	0	0.772589	-2.681009	-2.167935
3	6	0	1.315870	-1.266223	-2.152325
4	6	0	1.049297	-0.449948	-1.050248
5	6	0	0.312984	-0.742534	0.249575
6	6	0	-1.158541	-1.048705	-0.153303
7	6	0	-1.355950	-2.427638	-0.849121
8	8	0	0.630436	1.629291	-0.087077
9	6	0	1.152550	0.933087	-1.162224
10	6	0	0.582166	0.622177	0.951104
11	6	0	1.854599	-0.649382	-3.291167
12	6	0	2.060008	0.734761	-3.355094
13	6	0	1.677697	1.558268	-2.293044

14	6	0	0.846169	-1.777852	1.270731
15	6	0	0.102534	-1.615749	2.622097
16	6	0	0.081623	-0.179358	3.197639
17	6	0	-0.300258	0.895291	2.147526
18	8	0	1.798816	2.916588	-2.380203
19	8	0	-0.729234	-0.100906	4.363922
20	1	0	-1.061297	-2.101672	-2.847521
21	1	0	1.499475	3.291650	-1.534927
22	1	0	-1.636913	-0.328178	4.104616
23	1	0	1.095526	-3.193610	-3.082173
24	1	0	1.144729	-3.276275	-1.325172
25	1	0	-1.518519	-0.247872	-0.811703
26	1	0	-1.820412	-1.046329	0.722025
27	1	0	-1.074444	-3.225316	-0.149939
28	1	0	-2.432706	-2.552343	-1.019964
29	1	0	1.619745	0.557129	1.318411
30	1	0	2.093988	-1.256053	-4.161684
31	1	0	2.480290	1.196384	-4.243492
32	1	0	1.922678	-1.621815	1.414055
33	1	0	0.731155	-2.808382	0.923665
34	1	0	0.546330	-2.276886	3.374951
35	1	0	-0.938343	-1.949694	2.504285
36	1	0	1.089614	0.060992	3.557839
37	1	0	-1.365136	0.828884	1.891116
38	1	0	-0.121645	1.891213	2.566877

Cartesian coordinate of SSS-inv-N_c5:

Center Number	Atomic Number	Atomic Type	Coordinates (Angstroms) X	Y	Z

1	7	0	-0.799377	-2.453791	-1.175322
2	6	0	0.674013	-2.532776	-1.137759
3	6	0	1.325270	-1.165965	-1.190404
4	6	0	0.681421	-0.030744	-0.690768
5	6	0	-0.702046	0.166614	-0.084069
6	6	0	-1.713259	-0.103125	-1.237975
7	6	0	-1.383638	-1.405051	-2.025959
8	8	0	0.329442	2.273451	-0.632228
9	6	0	1.168759	1.238836	-1.001314
10	6	0	-0.532674	1.648307	0.348591
11	6	0	2.542332	-0.967872	-1.862583
12	6	0	3.075086	0.307052	-2.075997
13	6	0	2.373139	1.444922	-1.668674
14	6	0	-1.130901	-0.553451	1.217354
15	6	0	-2.412335	0.106635	1.790671
16	6	0	-2.366780	1.644954	1.937655
17	6	0	-1.829795	2.351725	0.677665
18	8	0	2.846990	2.697570	-1.941197
19	8	0	-3.662507	2.180349	2.197204
20	1	0	-1.170541	-3.362987	-1.430720
21	1	0	2.220499	3.334331	-1.557642
22	1	0	-3.996141	1.761604	3.006165
23	1	0	1.079762	-3.121744	-1.979175
24	1	0	0.953339	-3.072985	-0.220401
25	1	0	-1.705756	0.738367	-1.942697
26	1	0	-2.731210	-0.170415	-0.841301
27	1	0	-2.315751	-1.794946	-2.449859
28	1	0	-0.732762	-1.162854	-2.879247
29	1	0	0.072750	1.617640	1.269778
30	1	0	3.078050	-1.828837	-2.255549
31	1	0	4.018416	0.436732	-2.597668
32	1	0	-0.316502	-0.489406	1.951620

33	1	0	-1.317147	-1.611432	1.023030
34	1	0	-2.634238	-0.335606	2.772398
35	1	0	-3.272425	-0.126352	1.151472
36	1	0	-1.686672	1.896969	2.770059
37	1	0	-2.560143	2.275368	-0.135017
38	1	0	-1.674628	3.414797	0.891569

Cartesian coordinate of SSS-inv-N_c7:

Center Number	Atomic Number	Atomic Type	Coordinates (Angstroms) X	Y	Z
1	7	0	-0.689371	-2.449324	-1.139560
2	6	0	0.784288	-2.527438	-1.097135
3	6	0	1.434808	-1.160453	-1.150957
4	6	0	0.791381	-0.025874	-0.649357
5	6	0	-0.590673	0.169319	-0.038783
6	6	0	-1.604094	-0.099834	-1.191259
7	6	0	-1.272413	-1.397273	-1.985855
8	8	0	0.438237	2.278000	-0.586927
9	6	0	1.277569	1.244213	-0.959194
10	6	0	-0.421302	1.650547	0.395512
11	6	0	2.650385	-0.961157	-1.825361
12	6	0	3.182234	0.314228	-2.038295
13	6	0	2.480855	1.451483	-1.628251
14	6	0	-1.015473	-0.554938	1.262149
15	6	0	-2.294181	0.098635	1.848306
16	6	0	-2.253731	1.636341	1.999498
17	6	0	-1.717903	2.350658	0.733485
18	8	0	2.954137	2.704327	-1.900112
19	8	0	-3.514842	2.152786	2.411764

20	1	0	-1.059569	-3.357656	-1.399159
21	1	0	2.330955	3.340873	-1.510882
22	1	0	-4.162633	1.915283	1.728573
23	1	0	1.192501	-3.118382	-1.935764
24	1	0	1.060923	-3.064983	-0.177503
25	1	0	-1.602960	0.744368	-1.892995
26	1	0	-2.620767	-0.175444	-0.791583
27	1	0	-2.203644	-1.785370	-2.413449
28	1	0	-0.620638	-1.149167	-2.836680
29	1	0	0.186041	1.619298	1.315316
30	1	0	3.185903	-1.821488	-2.219895
31	1	0	4.124727	0.444802	-2.561224
32	1	0	-0.198998	-0.491594	1.993985
33	1	0	-1.199279	-1.612764	1.064705
34	1	0	-2.517684	-0.336327	2.829205
35	1	0	-3.154211	-0.149804	1.209038
36	1	0	-1.579572	1.888084	2.827659
37	1	0	-2.442264	2.278362	-0.088346
38	1	0	-1.565345	3.413252	0.951295

Cartesian coordinate of SSS-inv-N_c6:

Center Number	Atomic Number	Atomic Type	Coordinates (Angstroms) X	Y	Z
1	7	0	-0.970691	-2.446829	-1.110043
2	6	0	0.501837	-2.530250	-1.072400
3	6	0	1.156980	-1.165406	-1.129462

4	6	0	0.515278	-0.025884	-0.636811
5	6	0	-0.868721	0.178173	-0.033020
6	6	0	-1.879695	-0.095066	-1.186275
7	6	0	-1.552627	-1.402287	-1.966732
8	8	0	0.171321	2.280351	-0.589518
9	6	0	1.008260	1.240463	-0.951900
10	6	0	-0.695569	1.661262	0.391385
11	6	0	2.376578	-0.974366	-1.799171
12	6	0	2.914361	0.297596	-2.017040
13	6	0	2.215029	1.439547	-1.616796
14	6	0	-1.302608	-0.532516	1.272339
15	6	0	-2.583275	0.126515	1.845352
16	6	0	-2.535037	1.658545	1.985607
17	6	0	-1.994757	2.362236	0.714919
18	8	0	2.693883	2.689619	-1.893750
19	8	0	-3.859477	2.076815	2.305573
20	1	0	-1.346163	-3.356719	-1.356163
21	1	0	2.068510	3.329787	-1.514255
22	1	0	-3.835448	3.032353	2.471508
23	1	0	0.906284	-3.123002	-1.911930
24	1	0	0.779794	-3.068023	-0.153244

25	1	0	-1.869956	0.742130	-1.896280
26	1	0	-2.897491	-0.158281	-0.788669
27	1	0	-2.485533	-1.792509	-2.388496
28	1	0	-0.901056	-1.166342	-2.821337
29	1	0	-0.093787	1.634308	1.315137
30	1	0	2.910414	-1.839001	-2.186579
31	1	0	3.859589	0.421680	-2.536626
32	1	0	-0.487981	-0.467246	2.006416
33	1	0	-1.489979	-1.591064	1.082826
34	1	0	-2.810098	-0.301370	2.828588
35	1	0	-3.445408	-0.108529	1.209836
36	1	0	-1.853023	1.910847	2.816349
37	1	0	-2.724346	2.279198	-0.097722
38	1	0	-1.832209	3.429819	0.915553

5.2. Compounds **2** and **3**

5.2.1. SRRR configuration

Conformer	Population (%)
SRRR_c2.log	34.0872
SRRR_c1.log	26.96617
SRRR_c5.log	23.34481
SRRR_c3.log	4.854899
SRRR_c4.log	4.422376
SRRR_c0.log	3.857006

SRRR_c12.log	1.413027
SRRR_c6.log	1.05451

Cartesian coordinate of SRRR_c2:

Center Number	Atomic Number	Atomic Type	Coordinates (Angstroms) X	Y	Z
1	6	0	2.273749	-1.950178	-0.703234
2	6	0	3.829183	-1.994523	-0.607393
3	6	0	4.515418	-0.648928	-0.588582
4	6	0	3.826971	0.488170	-0.484744
5	6	0	2.327268	0.484978	-0.460169
6	7	0	1.992641	1.667163	0.319746
7	6	0	0.581758	1.984389	0.187345
8	6	0	-0.295192	0.741833	0.313968
9	6	0	-1.676977	0.944812	0.465670
10	6	0	-2.550170	-0.131625	0.540371
11	6	0	-2.047807	-1.444386	0.466196
12	6	0	-0.683398	-1.651566	0.324463
13	6	0	0.210526	-0.570678	0.239383
14	6	0	1.719583	-0.776487	0.139411
15	6	0	4.278224	1.922057	-0.265412
16	6	0	2.941842	2.694745	-0.110773
17	8	0	1.776637	-3.232117	-0.359026
18	8	0	4.115715	-2.693727	0.633631
19	8	0	-2.900445	-2.508550	0.540646
20	8	0	-3.917941	-0.062395	0.689775
21	6	0	-4.516312	1.219497	0.777512
22	1	0	2.341583	-3.522449	0.381742
23	1	0	4.958836	-3.159213	0.534097
24	1	0	-3.796540	-2.142890	0.639483
25	1	0	1.997468	-1.786570	-1.752599

26	1	0	4.191166	-2.612489	-1.441480
27	1	0	5.605612	-0.658335	-0.579212
28	1	0	1.948060	0.588326	-1.504723
29	1	0	0.378250	2.465036	-0.795652
30	1	0	0.302127	2.720026	0.953537
31	1	0	-2.056702	1.960806	0.522626
32	1	0	-0.310814	-2.666677	0.266718
33	1	0	2.126888	-0.881193	1.155561
34	1	0	4.882306	2.314670	-1.091152
35	1	0	4.884778	1.993818	0.644778
36	1	0	2.996893	3.509297	0.619779
37	1	0	2.646203	3.137426	-1.083467
38	1	0	-4.336015	1.807632	-0.131962
39	1	0	-4.142116	1.776296	1.646652
40	1	0	-5.588440	1.048040	0.891333

Cartesian coordinate of SRRR_c1:

Center Number	Atomic Number	Atomic Type	Coordinates (Angstroms) X	Y	Z
1	6	0	2.250938	-1.968897	-0.375670
2	6	0	3.815541	-2.021806	-0.252534
3	6	0	4.513517	-0.686312	-0.214429
4	6	0	3.842988	0.464963	-0.243187
5	6	0	2.347051	0.486194	-0.339589
6	7	0	1.978587	1.726421	0.327173
7	6	0	0.589147	2.057102	0.070516
8	6	0	-0.319758	0.841376	0.224585
9	6	0	-1.704655	1.075136	0.253940
10	6	0	-2.603068	0.020673	0.341311

11	6	0	-2.123040	-1.300988	0.402455
12	6	0	-0.756206	-1.537707	0.381459
13	6	0	0.164322	-0.479872	0.287978
14	6	0	1.673760	-0.714068	0.312513
15	6	0	4.307566	1.902007	-0.080174
16	6	0	2.981413	2.706207	-0.091898
17	8	0	1.737886	-3.198484	0.125374
18	8	0	4.172149	-2.706505	0.971594
19	8	0	-2.999381	-2.344606	0.488290
20	8	0	-3.976069	0.120614	0.379699
21	6	0	-4.553906	1.414094	0.325241
22	1	0	2.102441	-3.265054	1.026209
23	1	0	4.224391	-3.651465	0.759468
24	1	0	-3.893239	-1.960463	0.490483
25	1	0	1.979502	-1.950201	-1.437591
26	1	0	4.190796	-2.617230	-1.098081
27	1	0	5.595601	-0.722417	-0.101225
28	1	0	2.046476	0.513978	-1.413650
29	1	0	0.464571	2.466713	-0.956805
30	1	0	0.272282	2.851817	0.759372
31	1	0	-2.066610	2.097971	0.204755
32	1	0	-0.404309	-2.560872	0.426316
33	1	0	2.010699	-0.733418	1.360501
34	1	0	4.987891	2.226783	-0.875436
35	1	0	4.836850	2.024864	0.871726
36	1	0	2.993422	3.568213	0.584156
37	1	0	2.776527	3.085326	-1.113539
38	1	0	-4.293939	1.928509	-0.609216
39	1	0	-4.235157	2.029770	1.176505
40	1	0	-5.634674	1.266732	0.369144

Cartesian coordinate of SRRR_c5:

Center Number	Atomic Number	Atomic Type	Coordinates (Angstroms) X	Y	Z
1	6	0	2.199185	-1.921732	-0.855277
2	6	0	3.754115	-1.980004	-0.783554
3	6	0	4.436944	-0.638214	-0.803707
4	6	0	3.752706	0.508500	-0.756779
5	6	0	2.245502	0.541829	-0.736472
6	7	0	1.904124	1.827054	-0.094785
7	6	0	0.791840	1.773388	0.855313
8	6	0	-0.257998	0.759034	0.451960
9	6	0	-1.623153	1.060985	0.532364
10	6	0	-2.570325	0.095385	0.205776
11	6	0	-2.156930	-1.184536	-0.208985
12	6	0	-0.801283	-1.485053	-0.294137
13	6	0	0.156148	-0.518057	0.034673
14	6	0	1.658136	-0.716310	-0.059528
15	6	0	4.233351	1.917276	-0.526025
16	6	0	3.131170	2.445096	0.414924
17	8	0	1.689484	-3.174914	-0.437399
18	8	0	4.140953	-2.696191	0.416521
19	8	0	-3.085908	-2.131035	-0.530069
20	8	0	-3.938164	0.255702	0.238918
21	6	0	-4.454358	1.514034	0.639180
22	1	0	2.315929	-3.484906	0.244348
23	1	0	4.142370	-2.048708	1.141105
24	1	0	-3.959440	-1.721181	-0.404569
25	1	0	1.903002	-1.795321	-1.905313
26	1	0	4.107323	-2.628764	-1.592510
27	1	0	5.525608	-0.650279	-0.766650

28	1	0	1.853585	0.547786	-1.767759
29	1	0	0.341634	2.771399	0.941313
30	1	0	1.165954	1.518900	1.870546
31	1	0	-1.932409	2.052639	0.847264
32	1	0	-0.503300	-2.481105	-0.597992
33	1	0	2.069490	-0.774227	0.963392
34	1	0	4.226602	2.489959	-1.463323
35	1	0	5.237455	1.976339	-0.095744
36	1	0	3.350986	2.138692	1.457319
37	1	0	3.049497	3.539053	0.406670
38	1	0	-4.126629	2.313692	-0.037915
39	1	0	-4.150575	1.762378	1.664603
40	1	0	-5.541442	1.424765	0.595109

Cartesian coordinate of SRRR_c3:

Center Number	Atomic Number	Atomic Type	Coordinates (Angstroms) X	Y	Z
1	6	0	2.218671	-1.978267	-0.870474
2	6	0	3.770300	-2.024407	-0.800601
3	6	0	4.438149	-0.673002	-0.825717
4	6	0	3.744687	0.465626	-0.776127
5	6	0	2.237555	0.482697	-0.732841
6	7	0	1.893926	1.758190	-0.071884
7	6	0	0.801544	1.679802	0.899391
8	6	0	-0.246547	0.662493	0.499416
9	6	0	-1.612573	0.952606	0.603022
10	6	0	-2.557101	-0.015299	0.275436
11	6	0	-2.139611	-1.285045	-0.164110
12	6	0	-0.782926	-1.573795	-0.272006

13	6	0	0.172553	-0.605352	0.058522
14	6	0	1.675735	-0.787342	-0.054996
15	6	0	4.214427	1.877638	-0.542286
16	6	0	3.123836	2.382969	0.424015
17	8	0	1.714521	-3.244325	-0.483665
18	8	0	4.084029	-2.682993	0.456610
19	8	0	-3.066240	-2.234337	-0.487086
20	8	0	-3.926271	0.134150	0.330054
21	6	0	-4.445077	1.381698	0.758504
22	1	0	2.270867	-3.509879	0.272086
23	1	0	4.887767	-3.208228	0.333004
24	1	0	-3.940480	-1.832423	-0.342514
25	1	0	1.924500	-1.839199	-1.918821
26	1	0	4.122550	-2.663959	-1.622059
27	1	0	5.527654	-0.666887	-0.792256
28	1	0	1.825741	0.496619	-1.756590
29	1	0	0.344055	2.672250	1.009623
30	1	0	1.197449	1.412097	1.902517
31	1	0	-1.924582	1.937341	0.936486
32	1	0	-0.481851	-2.562815	-0.594880
33	1	0	2.107957	-0.852206	0.956190
34	1	0	4.185228	2.461301	-1.472526
35	1	0	5.224593	1.942710	-0.127237
36	1	0	3.365252	2.065750	1.457700
37	1	0	3.030021	3.476185	0.429572
38	1	0	-4.133277	2.194957	0.090056
39	1	0	-4.127632	1.615784	1.783193
40	1	0	-5.532160	1.285552	0.729284

Cartesian coordinate of SRRR_c4:

Center Number	Atomic Number	Atomic Type	Coordinates (Angstroms) X	Y	Z
1	6	0	2.295389	-1.723759	-0.514313
2	6	0	3.848309	-1.790276	-0.350385
3	6	0	4.538292	-0.446107	-0.356449
4	6	0	3.850929	0.694502	-0.333954
5	6	0	2.350079	0.698395	-0.364200
6	7	0	1.992008	1.910034	0.355990
7	6	0	0.591292	2.229432	0.151413
8	6	0	-0.298226	0.999098	0.300609
9	6	0	-1.682980	1.217640	0.392842
10	6	0	-2.567862	0.152950	0.491881
11	6	0	-2.072619	-1.164142	0.509224
12	6	0	-0.705718	-1.385482	0.428464
13	6	0	0.201024	-0.318130	0.310302
14	6	0	1.712637	-0.536126	0.267348
15	6	0	4.300817	2.134717	-0.154311
16	6	0	2.964261	2.917431	-0.073625
17	8	0	1.766475	-2.975214	-0.036519
18	8	0	4.171137	-2.414872	0.889493
19	8	0	-2.933647	-2.219334	0.612589
20	8	0	-3.938774	0.237625	0.587293
21	6	0	-4.529568	1.526665	0.601544
22	1	0	1.826869	-3.619193	-0.759371
23	1	0	3.479285	-3.092037	1.011092
24	1	0	-3.829763	-1.844844	0.670629
25	1	0	2.045725	-1.606521	-1.578460
26	1	0	4.234762	-2.411012	-1.181972
27	1	0	5.624223	-0.467843	-0.285133
28	1	0	2.003677	0.762147	-1.423439
29	1	0	0.430620	2.665187	-0.860190

30	1	0	0.285405	3.000491	0.871189
31	1	0	-2.055477	2.237790	0.384822
32	1	0	-0.343827	-2.404830	0.458901
33	1	0	2.092783	-0.610676	1.295410
34	1	0	4.931894	2.494897	-0.974617
35	1	0	4.878311	2.239516	0.771176
36	1	0	2.996156	3.755529	0.631156
37	1	0	2.705097	3.328596	-1.070193
38	1	0	-4.316082	2.073705	-0.326075
39	1	0	-4.178071	2.117044	1.457705
40	1	0	-5.605945	1.367028	0.688384

Cartesian coordinate of SRRR_c0:

Center Number	Atomic Number	Atomic Type	Coordinates (Angstroms) X	Y	Z
1	6	0	2.149981	-2.017358	-0.812036
2	6	0	3.707658	-2.102536	-0.724410
3	6	0	4.406838	-0.770757	-0.677516
4	6	0	3.747074	0.387897	-0.690370
5	6	0	2.241747	0.450691	-0.739423
6	7	0	1.898773	1.746739	-0.119033
7	6	0	0.752704	1.722069	0.790911
8	6	0	-0.301079	0.724858	0.358396
9	6	0	-1.662108	1.053547	0.382906
10	6	0	-2.615125	0.105160	0.024271
11	6	0	-2.210673	-1.183835	-0.369319
12	6	0	-0.858666	-1.510396	-0.399418
13	6	0	0.105096	-0.562394	-0.034539
14	6	0	1.607218	-0.787597	-0.066900

15	6	0	4.247162	1.788082	-0.449737
16	6	0	3.116307	2.346857	0.436375
17	8	0	1.609455	-3.255668	-0.355317
18	8	0	4.084145	-2.804972	0.477791
19	8	0	-3.144192	-2.114280	-0.724174
20	8	0	-3.979902	0.292079	0.003245
21	6	0	-4.486189	1.563080	0.375673
22	1	0	1.844070	-3.307270	0.586963
23	1	0	3.971488	-3.749606	0.285827
24	1	0	-4.013705	-1.686394	-0.636329
25	1	0	1.856446	-1.949097	-1.866231
26	1	0	4.059371	-2.685444	-1.589748
27	1	0	5.488818	-0.806639	-0.564213
28	1	0	1.886835	0.455480	-1.784118
29	1	0	0.318507	2.729062	0.853047
30	1	0	1.084731	1.468808	1.820675
31	1	0	-1.963420	2.053222	0.679582
32	1	0	-0.570303	-2.512781	-0.691956
33	1	0	1.990044	-0.834505	0.966457
34	1	0	4.297897	2.354875	-1.389491
35	1	0	5.230419	1.827164	0.028120
36	1	0	3.284510	2.046814	1.489326
37	1	0	3.054529	3.442105	0.413104
38	1	0	-4.116530	2.351545	-0.292825
39	1	0	-4.217526	1.812164	1.410629
40	1	0	-5.572305	1.494861	0.289799

Cartesian coordinate of SRRR_c12:

Center Number	Atomic Number	Atomic Type	Coordinates (Angstroms) X	Y	Z

1	6	0	2.242151	-1.894477	-0.726611
2	6	0	3.802327	-1.945299	-0.650941
3	6	0	4.497408	-0.606691	-0.620198
4	6	0	3.814889	0.534201	-0.492429
5	6	0	2.315391	0.541820	-0.463535
6	7	0	1.990845	1.715434	0.333081
7	6	0	0.579669	2.044837	0.213353
8	6	0	-0.305169	0.806104	0.318349
9	6	0	-1.687504	1.008476	0.466852
10	6	0	-2.583388	-0.055256	0.521741
11	6	0	-2.078964	-1.370313	0.426414
12	6	0	-0.712814	-1.574960	0.290344
13	6	0	0.194070	-0.503406	0.225423
14	6	0	1.698788	-0.721409	0.120953
15	6	0	4.275924	1.962898	-0.261178
16	6	0	2.945326	2.742557	-0.088834
17	8	0	1.751020	-3.172806	-0.353835
18	8	0	4.185022	-2.709617	0.521186
19	8	0	-2.984015	-2.394613	0.480928
20	8	0	-3.935961	0.055642	0.664392
21	6	0	-4.486838	1.355864	0.764202
22	1	0	2.393418	-3.496823	0.307670
23	1	0	4.183446	-2.090508	1.270357
24	1	0	-2.494455	-3.227493	0.392722
25	1	0	1.945821	-1.735634	-1.771655
26	1	0	4.157550	-2.561831	-1.483885
27	1	0	5.586408	-0.630908	-0.621674
28	1	0	1.936981	0.657925	-1.506369
29	1	0	0.375591	2.546462	-0.758268
30	1	0	0.309331	2.766522	0.995697
31	1	0	-2.058679	2.026184	0.537235

32	1	0	-0.327063	-2.587870	0.219158
33	1	0	2.101411	-0.833920	1.140844
34	1	0	4.876349	2.357622	-1.088323
35	1	0	4.891575	2.022872	0.643773
36	1	0	3.010203	3.546349	0.652499
37	1	0	2.647947	3.200385	-1.053431
38	1	0	-4.285503	1.952560	-0.136299
39	1	0	-4.102945	1.893427	1.642307
40	1	0	-5.564466	1.216831	0.870201

Cartesian coordinate of SRRR_c6:

Center Number	Atomic Number	Atomic Type	Coordinates (Angstroms) X	Y	Z
1	6	0	2.380734	-1.943152	-0.578234
2	6	0	3.943210	-1.952657	-0.608427
3	6	0	4.601739	-0.597608	-0.682113
4	6	0	3.898919	0.531140	-0.556535
5	6	0	2.405014	0.505034	-0.425145
6	7	0	2.104792	1.703444	0.343148
7	6	0	0.680667	1.995748	0.307644
8	6	0	-0.163024	0.743004	0.523856
9	6	0	-1.534714	0.906732	0.749354
10	6	0	-2.396036	-0.173139	0.914920
11	6	0	-1.863501	-1.474367	0.856773
12	6	0	-0.501378	-1.651148	0.626638
13	6	0	0.364060	-0.562313	0.451885
14	6	0	1.864096	-0.746453	0.252902
15	6	0	4.336898	1.978862	-0.417600
16	6	0	3.001568	2.733982	-0.184274

17	8	0	1.951013	-3.214802	-0.116940
18	8	0	4.424558	-2.655408	0.565763
19	8	0	-2.716641	-2.531318	1.048115
20	8	0	-3.717381	0.073476	1.200159
21	6	0	-4.666145	-0.404363	0.243757
22	1	0	2.646670	-3.493429	0.510694
23	1	0	4.461637	-2.003512	1.285638
24	1	0	-2.201040	-3.350281	0.979001
25	1	0	2.009825	-1.838908	-1.606197
26	1	0	4.257516	-2.596411	-1.437217
27	1	0	5.688415	-0.595439	-0.756926
28	1	0	1.953917	0.566882	-1.443538
29	1	0	0.398441	2.450352	-0.667188
30	1	0	0.444321	2.742691	1.076602
31	1	0	-1.968318	1.902238	0.807866
32	1	0	-0.089544	-2.655452	0.580009
33	1	0	2.335442	-0.802493	1.247440
34	1	0	4.867720	2.351309	-1.300770
35	1	0	5.012405	2.092005	0.438201
36	1	0	3.095992	3.568723	0.518558
37	1	0	2.626963	3.144725	-1.143055
38	1	0	-4.474188	0.030361	-0.746794
39	1	0	-5.644018	-0.070976	0.599422
40	1	0	-4.653112	-1.495878	0.172732

5.2.2. SRRS configuration

Conformer	Population (%)
SRRS_c0.log	100

Cartesian coordinate of SRRS_c0:

Center Number	Atomic Number	Atomic Type	Coordinates (Angstroms) X	Y	Z
1	6	0	2.251758	-0.642654	-0.222748
2	6	0	3.230219	-1.828238	-0.028887
3	6	0	2.454650	-3.127522	0.082956
4	6	0	1.193959	-3.165102	0.519913
5	6	0	0.441277	-1.939153	0.972767
6	7	0	-0.683967	-1.979566	0.019726
7	6	0	-1.713100	-0.997533	0.325632
8	6	0	-1.116179	0.384784	0.517300
9	6	0	-1.965956	1.503246	0.435799
10	6	0	-1.474388	2.784406	0.653507
11	6	0	-0.110841	2.967781	0.961427
12	6	0	0.725647	1.865461	1.038440
13	6	0	0.240660	0.565226	0.814603
14	6	0	1.203282	-0.611121	0.906574
15	6	0	0.142099	-4.233301	0.313783
16	6	0	-1.137898	-3.387563	0.015022
17	8	0	1.658962	-0.727465	-1.520908
18	8	0	4.129912	-1.886258	-1.132806
19	8	0	0.385620	4.220285	1.184333
20	8	0	-2.199565	3.951769	0.600648
21	6	0	-3.583642	3.877520	0.277963
22	1	0	0.068898	-2.086217	2.009556
23	1	0	1.771106	-0.513161	1.842537
24	1	0	2.835110	0.284208	-0.220064
25	1	0	3.856810	-1.644194	0.853675
26	1	0	2.933784	-4.007855	-0.341456
27	1	0	-2.267538	-1.288921	1.242985
28	1	0	-2.443760	-0.982919	-0.492295
29	1	0	-3.014311	1.352825	0.198677

30	1	0	1.771986	2.033984	1.276570
31	1	0	-0.010936	-4.841943	1.212820
32	1	0	0.402446	-4.909783	-0.504330
33	1	0	-1.596328	-3.633220	-0.948111
34	1	0	-1.897162	-3.550381	0.799464
35	1	0	0.822528	-1.222028	-1.439797
36	1	0	3.588392	-1.729504	-1.924275
37	1	0	-0.340354	4.854535	1.080956
38	1	0	-3.949039	4.904988	0.283684
39	1	0	-3.733152	3.440805	-0.716861
40	1	0	-4.131785	3.289980	1.024519

5.2.3. SRSR configuration

Conformer	Population (%)
SRSR_c1.log	29.414
SRSR_c2.log	26.148
SRSR_c0.log	25.872
SRSR_c3.log	18.565

Cartesian coordinate of SRSR_c1:

Center Number	Atomic Number	Atomic Type	Coordinates (Angstroms) X	Y	Z
1	6	0	-0.760458	-1.400331	3.257812
2	6	0	0.782924	-1.424850	3.236894
3	6	0	1.389003	-0.049204	3.238216
4	6	0	0.664749	1.031137	2.947949
5	6	0	-0.803466	0.991957	2.555899
6	7	0	-0.894715	1.974395	1.452421
7	6	0	-0.282903	1.462632	0.215043
8	6	0	-0.775598	0.086137	-0.166977

9	6	0	-0.722022	-0.300378	-1.517756
10	6	0	-1.124243	-1.569758	-1.907743
11	6	0	-1.608836	-2.476327	-0.943643
12	6	0	-1.671095	-2.089079	0.385917
13	6	0	-1.240503	-0.814004	0.796214
14	6	0	-1.375313	-0.400035	2.253292
15	6	0	1.109317	2.469631	2.765854
16	6	0	-0.093126	3.097739	1.981616
17	8	0	-1.210561	-1.028672	4.552332
18	8	0	1.250797	-2.108677	4.421070
19	8	0	-2.025104	-3.720396	-1.319358
20	8	0	-1.122109	-2.070018	-3.189553
21	6	0	-0.683251	-1.220767	-4.237865
22	1	0	-0.520877	-1.355483	5.159769
23	1	0	1.095609	-3.058484	4.289211
24	1	0	-1.914797	-3.773513	-2.284743
25	1	0	-1.116210	-2.417908	3.022692
26	1	0	1.123965	-1.977156	2.347370
27	1	0	2.455700	0.006772	3.450249
28	1	0	-1.383667	1.409572	3.390241
29	1	0	0.822323	1.429124	0.283023
30	1	0	-0.517945	2.174585	-0.586213
31	1	0	-0.363872	0.411427	-2.255266
32	1	0	-2.078460	-2.800335	1.098027
33	1	0	-2.447932	-0.379341	2.488907
34	1	0	2.051735	2.536326	2.211447
35	1	0	1.268112	2.975436	3.726306
36	1	0	-0.725581	3.680417	2.661087
37	1	0	0.218845	3.759401	1.168348
38	1	0	0.361293	-0.916179	-4.092567
39	1	0	-1.313573	-0.325512	-4.315284
40	1	0	-0.766473	-1.804272	-5.156547

Cartesian coordinate of SRSR_c2:

Center Number	Atomic Number	Atomic Type	Coordinates (Angstroms) X	Y	Z
1	6	0	-0.808142	-1.607486	3.278661
2	6	0	0.738755	-1.647940	3.259535
3	6	0	1.371335	-0.282745	3.238007
4	6	0	0.655535	0.824407	3.036367
5	6	0	-0.828190	0.822916	2.705535
6	7	0	-0.951168	1.870305	1.667057
7	6	0	-0.398148	1.431961	0.374270
8	6	0	-0.899285	0.076012	-0.064555
9	6	0	-0.889141	-0.233237	-1.436197
10	6	0	-1.296491	-1.481692	-1.883421
11	6	0	-1.740592	-2.445345	-0.955764
12	6	0	-1.758675	-2.134439	0.394911
13	6	0	-1.323789	-0.880588	0.861711
14	6	0	-1.422154	-0.544956	2.342643
15	6	0	1.118993	2.262357	2.902840
16	6	0	-0.114222	2.951267	2.226494
17	8	0	-1.264829	-1.304788	4.593089
18	8	0	1.185015	-2.405496	4.408937
19	8	0	-2.161783	-3.669318	-1.386307
20	8	0	-1.335229	-1.908853	-3.190649
21	6	0	-0.929617	-1.001356	-4.202774
22	1	0	-0.774083	-1.919815	5.165778
23	1	0	1.465769	-1.755798	5.072912
24	1	0	-2.078137	-3.670557	-2.355772
25	1	0	-1.168367	-2.605738	2.990629

26	1	0	1.067069	-2.228864	2.387918
27	1	0	2.453547	-0.248106	3.373662
28	1	0	-1.371958	1.194726	3.585132
29	1	0	0.709124	1.403545	0.387297
30	1	0	-0.674297	2.187517	-0.372094
31	1	0	-0.560334	0.521744	-2.143858
32	1	0	-2.132129	-2.890447	1.078565
33	1	0	-2.489839	-0.522234	2.599123
34	1	0	2.027304	2.340754	2.295379
35	1	0	1.347535	2.712706	3.876791
36	1	0	-0.704509	3.491416	2.975576
37	1	0	0.162900	3.663438	1.444017
38	1	0	0.118115	-0.701220	-4.071653
39	1	0	-1.563989	-0.105611	-4.212028
40	1	0	-1.038809	-1.533510	-5.149487

Cartesian coordinate of SRSR_c0:

Center Number	Atomic Number	Atomic Type	Coordinates (Angstroms) X	Y	Z
1	6	0	-0.743106	-1.504965	3.256512
2	6	0	0.798303	-1.528773	3.213509
3	6	0	1.403464	-0.148687	3.221386
4	6	0	0.675491	0.940535	2.977624
5	6	0	-0.800434	0.904788	2.614469
6	7	0	-0.916866	1.918473	1.542578
7	6	0	-0.331075	1.447964	0.276224
8	6	0	-0.816163	0.076887	-0.133641
9	6	0	-0.784451	-0.270329	-1.495762
10	6	0	-1.177521	-1.533642	-1.913447

11	6	0	-1.628660	-2.474312	-0.965807
12	6	0	-1.668674	-2.126053	0.375343
13	6	0	-1.248887	-0.856536	0.812630
14	6	0	-1.367242	-0.482514	2.281918
15	6	0	1.115181	2.383317	2.820129
16	6	0	-0.109517	3.029407	2.086747
17	8	0	-1.181060	-1.149441	4.560215
18	8	0	1.190986	-2.300450	4.369969
19	8	0	-2.035262	-3.712769	-1.368412
20	8	0	-1.194992	-1.997149	-3.208982
21	6	0	-0.786934	-1.113220	-4.240613
22	1	0	-0.543097	-1.580629	5.157761
23	1	0	2.081097	-2.019054	4.630192
24	1	0	-1.940054	-3.738956	-2.336447
25	1	0	-1.096392	-2.517596	3.010939
26	1	0	1.121066	-2.069908	2.310355
27	1	0	2.482784	-0.083209	3.381028
28	1	0	-1.366283	1.293463	3.472100
29	1	0	0.775941	1.426510	0.315937
30	1	0	-0.593734	2.179893	-0.498022
31	1	0	-0.450814	0.466884	-2.219723
32	1	0	-2.046536	-2.864594	1.075548
33	1	0	-2.437102	-0.471862	2.529895
34	1	0	2.041007	2.465059	2.240130
35	1	0	1.301813	2.864181	3.788406
36	1	0	-0.727152	3.586118	2.800743
37	1	0	0.179076	3.718600	1.287928
38	1	0	0.256468	-0.799459	-4.106572
39	1	0	-1.429442	-0.224135	-4.280641
40	1	0	-0.880699	-1.671443	-5.173897

Cartesian coordinate of SRSR_c3:

Center Number	Atomic Number	Atomic Type	Coordinates (Angstroms) X	Y	Z
1	6	0	-0.754279	-1.460448	3.359285
2	6	0	0.780518	-1.511471	3.252035
3	6	0	1.373570	-0.118264	3.329034
4	6	0	0.643881	0.978635	3.107028
5	6	0	-0.825781	0.956203	2.702366
6	7	0	-0.893881	1.942182	1.603043
7	6	0	-0.285795	1.420143	0.367929
8	6	0	-0.829822	0.066595	-0.024944
9	6	0	-0.808706	-0.307378	-1.380062
10	6	0	-1.265178	-1.556275	-1.776474
11	6	0	-1.771411	-2.454621	-0.814829
12	6	0	-1.799219	-2.080054	0.519424
13	6	0	-1.316847	-0.825648	0.934024
14	6	0	-1.409997	-0.434025	2.400741
15	6	0	1.088500	2.422660	2.965619
16	6	0	-0.085039	3.057351	2.141670
17	8	0	-1.109664	-1.192066	4.726934
18	8	0	1.305990	-2.371802	4.257255
19	8	0	-2.241138	-3.675938	-1.197166
20	8	0	-1.300022	-2.042431	-3.062250
21	6	0	-0.825496	-1.207488	-4.106883
22	1	0	-0.722927	-0.329580	4.956342
23	1	0	0.741172	-2.218509	5.036547
24	1	0	-2.136499	-3.728793	-2.163202
25	1	0	-1.151879	-2.460707	3.172905
26	1	0	1.060956	-1.966775	2.293251
27	1	0	2.442387	-0.064512	3.533413

28	1	0	-1.416965	1.395872	3.521984
29	1	0	0.816458	1.347827	0.448165
30	1	0	-0.489320	2.146542	-0.428467
31	1	0	-0.433350	0.397488	-2.115559
32	1	0	-2.217485	-2.784876	1.231898
33	1	0	-2.474756	-0.418814	2.668669
34	1	0	2.052335	2.498499	2.451599
35	1	0	1.205020	2.920010	3.936793
36	1	0	-0.725094	3.661030	2.795466
37	1	0	0.258893	3.702227	1.328187
38	1	0	0.232729	-0.953790	-3.963446
39	1	0	-1.413532	-0.283239	-4.175813
40	1	0	-0.939847	-1.780280	-5.028861

5.2.4. SRSS configuration

Conformer	Population (%)
SRSS_c0.log	65.675
SRSS_c1.log	23.681
SRSS_c3.log	6.712
SRSS_c2.log	2.181
SRSS_c4.log	1.751

Cartesian coordinate of SRSS_c0:

Center Number	Atomic Number	Atomic Type	Coordinates (Angstroms) X	Y	Z
1	6	0	-0.075783	-2.783812	0.595143
2	6	0	1.332100	-2.754002	0.619690
3	6	0	1.997642	-1.535019	0.645374
4	6	0	1.286738	-0.323388	0.640382
5	6	0	-0.120018	-0.350796	0.615710

6	6	0	-0.782590	-1.588926	0.598738
7	6	0	2.092872	0.975605	0.615975
8	7	0	1.260019	2.135692	0.882645
9	6	0	0.024056	2.057615	0.115832
10	6	0	-0.883176	0.966833	0.655827
11	6	0	-0.567160	3.429390	0.228178
12	6	0	-1.888172	3.607474	0.183058
13	6	0	-2.873426	2.450033	0.100013
14	6	0	-2.215951	1.057783	-0.107194
15	8	0	-0.738398	-3.974823	0.573200
16	6	0	3.329930	-4.080215	0.654989
17	8	0	1.913358	-4.000410	0.619347
18	6	0	0.584898	4.385330	0.498068
19	6	0	1.823609	3.448232	0.553107
20	8	0	-3.883412	2.665507	-0.878299
21	8	0	-2.026980	0.910304	-1.519832
22	1	0	-0.061828	-4.674373	0.584527
23	1	0	-3.490097	2.374519	-1.719554
24	1	0	-1.668905	0.022548	-1.677981
25	1	0	3.083126	-1.508361	0.666560
26	1	0	-1.867353	-1.644742	0.603967
27	1	0	2.587626	1.061820	-0.376989
28	1	0	2.899914	0.925437	1.359392
29	1	0	0.235563	1.839097	-0.952448
30	1	0	-1.095356	1.213822	1.707168
31	1	0	-2.342347	4.593197	0.274399
32	1	0	-3.412934	2.412728	1.057157
33	1	0	-2.932181	0.300882	0.243467
34	1	0	3.777853	-3.600579	-0.224827
35	1	0	3.573790	-5.144007	0.651912
36	1	0	3.731769	-3.616027	1.564749
37	1	0	0.438822	4.903172	1.452979

38	1	0	0.697202	5.154720	-0.274246
39	1	0	2.328843	3.433064	-0.433339
40	1	0	2.564350	3.756366	1.299296

Cartesian coordinate of SRSS_c1:

Center Number	Atomic Number	Atomic Type	Coordinates (Angstroms) X	Y	Z
1	6	0	0.111707	-2.761288	0.664038
2	6	0	1.519559	-2.736227	0.635299
3	6	0	2.188932	-1.519948	0.624899
4	6	0	1.478825	-0.307622	0.641139
5	6	0	0.073181	-0.331702	0.669446
6	6	0	-0.591597	-1.566188	0.684658
7	6	0	2.284533	0.988783	0.577671
8	7	0	1.469049	2.144144	0.910834
9	6	0	0.195878	2.093068	0.202632
10	6	0	-0.684952	0.985974	0.755048
11	6	0	-0.381513	3.459391	0.419385
12	6	0	-1.702521	3.645239	0.479108
13	6	0	-2.688382	2.505713	0.333330
14	6	0	-2.054981	1.094929	0.076164
15	8	0	-0.553593	-3.952768	0.675376
16	6	0	3.513074	-4.066464	0.574308
17	8	0	2.098275	-3.985714	0.622379
18	6	0	0.783130	4.401699	0.676301
19	6	0	2.020085	3.464985	0.606219
20	8	0	-3.648666	2.780392	-0.709180
21	8	0	-1.897847	0.874866	-1.318000
22	1	0	0.122932	-4.651712	0.651340

23	1	0	-3.161075	3.254894	-1.402174
24	1	0	-2.762184	1.106773	-1.700655
25	1	0	3.274458	-1.494880	0.602789
26	1	0	-1.675410	-1.616784	0.702683
27	1	0	2.716791	1.089578	-0.442378
28	1	0	3.135112	0.933745	1.270385
29	1	0	0.345564	1.919242	-0.882977
30	1	0	-0.845770	1.217750	1.819344
31	1	0	-2.136645	4.624956	0.678957
32	1	0	-3.292260	2.445843	1.248233
33	1	0	-2.750301	0.348451	0.492445
34	1	0	3.908968	-3.591650	-0.332909
35	1	0	3.756837	-5.130451	0.562985
36	1	0	3.969606	-3.597579	1.455785
37	1	0	0.690630	4.862389	1.666503
38	1	0	0.846617	5.214815	-0.055757
39	1	0	2.461869	3.498113	-0.409993
40	1	0	2.806702	3.737464	1.318460

Cartesian coordinate of SRSS_c3:

Center Number	Atomic Number	Atomic Type	Coordinates (Angstroms) X	Y	Z
1	6	0	0.329186	-2.775815	0.653188
2	6	0	1.737060	-2.747965	0.665428
3	6	0	2.404403	-1.530415	0.672254
4	6	0	1.692571	-0.319075	0.665024
5	6	0	0.286511	-0.346226	0.652241
6	6	0	-0.376329	-1.581747	0.651069
7	6	0	2.498553	0.978516	0.620003

8	7	0	1.674747	2.137331	0.921772
9	6	0	0.420966	2.074346	0.180355
10	6	0	-0.473381	0.970966	0.715649
11	6	0	-0.163731	3.443249	0.350989
12	6	0	-1.484159	3.633514	0.367449
13	6	0	-2.461120	2.482598	0.296035
14	6	0	-1.822973	1.091604	-0.007816
15	8	0	-0.334084	-3.968688	0.648102
16	6	0	3.733898	-4.074545	0.660596
17	8	0	2.318187	-3.996575	0.672282
18	6	0	0.996047	4.392343	0.605049
19	6	0	2.233611	3.452470	0.601156
20	8	0	-3.433806	2.782883	-0.729186
21	8	0	-1.636604	0.941225	-1.403857
22	1	0	0.344489	-4.666070	0.643478
23	1	0	-4.225411	2.250328	-0.546159
24	1	0	-2.307502	1.523414	-1.808791
25	1	0	3.490077	-1.503697	0.681568
26	1	0	-1.460272	-1.634162	0.638676
27	1	0	2.960025	1.072926	-0.387779
28	1	0	3.329055	0.926346	1.336988
29	1	0	0.600596	1.884971	-0.897591
30	1	0	-0.662616	1.201562	1.776041
31	1	0	-1.926123	4.618561	0.508435
32	1	0	-2.983582	2.418970	1.264412
33	1	0	-2.526031	0.322048	0.359176
34	1	0	4.151918	-3.600287	-0.236887
35	1	0	3.979932	-5.138066	0.657296
36	1	0	4.166807	-3.603385	1.552737
37	1	0	0.879023	4.889765	1.574678
38	1	0	1.081707	5.177519	-0.154650
39	1	0	2.707466	3.458082	-0.400836

40	1	0	2.997548	3.744352	1.330325

Cartesian coordinate of SRSS_c2:

Center Number	Atomic Number	Atomic Type	Coordinates (Angstroms) X	Y	Z
1	6	0	0.009711	-2.810089	0.520539
2	6	0	1.417084	-2.788724	0.529285
3	6	0	2.095597	-1.573275	0.551474
4	6	0	1.383221	-0.367441	0.566031
5	6	0	-0.021456	-0.386315	0.552684
6	6	0	-0.698518	-1.611717	0.529148
7	6	0	2.094681	0.968618	0.662849
8	7	0	1.521925	1.977348	-0.228027
9	6	0	0.069657	1.859512	-0.473451
10	6	0	-0.692687	0.972467	0.530315
11	6	0	-0.453743	3.272440	-0.435288
12	6	0	-1.719519	3.543761	-0.111120
13	6	0	-2.725525	2.477941	0.286332
14	6	0	-2.183721	1.031395	0.159957
15	8	0	-0.659179	-3.997084	0.497553
16	6	0	3.405777	-4.129464	0.511223
17	8	0	1.989275	-4.039732	0.509702
18	6	0	0.735013	4.185007	-0.601981
19	6	0	1.828898	3.369097	0.119601
20	8	0	-3.964830	2.606667	-0.398899
21	8	0	-2.411350	0.642788	-1.199925
22	1	0	0.014017	-4.700149	0.493576
23	1	0	-3.830030	2.166991	-1.256482
24	1	0	-2.073098	-0.258621	-1.318802

25	1	0	3.180742	-1.550222	0.555833
26	1	0	-1.783673	-1.667751	0.540038
27	1	0	3.162099	0.843171	0.434891
28	1	0	2.048685	1.312885	1.718675
29	1	0	-0.088829	1.420491	-1.468028
30	1	0	-0.593401	1.430035	1.528177
31	1	0	-2.077549	4.569996	-0.040969
32	1	0	-2.972373	2.642192	1.345003
33	1	0	-2.778913	0.390265	0.827047
34	1	0	3.836234	-3.645040	-0.374568
35	1	0	3.641883	-5.194855	0.493130
36	1	0	3.832293	-3.676545	1.415472
37	1	0	0.589870	5.181436	-0.174471
38	1	0	0.999128	4.300595	-1.662146
39	1	0	2.843949	3.635316	-0.201176
40	1	0	1.769736	3.548150	1.211690

Cartesian coordinate of SRSS_c4:

Center	Atomic	Atomic	Coordinates (Angstroms)		
Number	Number	Type	X	Y	Z
1	6	0	0.013699	-2.815248	0.545035
2	6	0	1.421420	-2.791193	0.558107
3	6	0	2.098502	-1.575549	0.566032
4	6	0	1.379495	-0.373414	0.578984
5	6	0	-0.024111	-0.397898	0.574542
6	6	0	-0.699456	-1.621491	0.544535
7	6	0	2.083987	0.965761	0.646802
8	7	0	1.492260	1.961887	-0.250706
9	6	0	0.028531	1.852164	-0.450546

10	6	0	-0.693652	0.961112	0.578122
11	6	0	-0.479243	3.269187	-0.371248
12	6	0	-1.720939	3.556966	0.029563
13	6	0	-2.726574	2.496120	0.405482
14	6	0	-2.198837	1.027407	0.305179
15	8	0	-0.649629	-4.007526	0.519123
16	6	0	3.413051	-4.125714	0.513126
17	8	0	1.998046	-4.042529	0.545864
18	6	0	0.702378	4.178040	-0.585066
19	6	0	1.823399	3.352175	0.078665
20	8	0	-3.935221	2.613066	-0.376942
21	8	0	-2.457872	0.504467	-0.989637
22	1	0	0.028676	-4.705066	0.502808
23	1	0	-3.660353	2.940303	-1.248950
24	1	0	-3.382218	0.752876	-1.169777
25	1	0	3.183602	-1.548607	0.560773
26	1	0	-1.781176	-1.668501	0.490523
27	1	0	3.147537	0.845122	0.400056
28	1	0	2.055766	1.330423	1.696545
29	1	0	-0.171985	1.423272	-1.441427
30	1	0	-0.540768	1.419133	1.569679
31	1	0	-2.046936	4.589961	0.151322
32	1	0	-3.052810	2.673189	1.438709
33	1	0	-2.738061	0.430854	1.059639
34	1	0	3.820410	-3.645104	-0.385913
35	1	0	3.654830	-5.190121	0.496839
36	1	0	3.860731	-3.664527	1.403237
37	1	0	0.580829	5.173174	-0.147164
38	1	0	0.912798	4.296167	-1.656817
39	1	0	2.822525	3.614319	-0.291542
40	1	0	1.819503	3.532525	1.172086

5.2.5. SSRR configuration

Conformer	Population (%)
SSRR_c0.log	31.954
SSRR_c1.log	25.711
SSRR_c2.log	18.685
SSRR_c3.log	16.006
SSRR_c4.log	3.184
SSRR_c7.log	2.611
SSRR_c5.log	1.850

Cartesian coordinate of SSRR_c0:

Center Number	Atomic Number	Atomic Type	Coordinates (Angstroms) X	Y	Z
1	6	0	2.264046	-1.898165	-0.650494
2	6	0	3.807883	-1.955586	-0.535599
3	6	0	4.502032	-0.616055	-0.562387
4	6	0	3.819184	0.523105	-0.453275
5	6	0	2.317048	0.532555	-0.406375
6	7	0	2.005588	1.697847	0.408330
7	6	0	0.596163	2.035812	0.312128
8	6	0	-0.299022	0.802159	0.393220
9	6	0	-1.678860	1.020135	0.541955
10	6	0	-2.570564	-0.043541	0.561633
11	6	0	-2.089528	-1.359575	0.426993
12	6	0	-0.727187	-1.581729	0.287132
13	6	0	0.185753	-0.513892	0.267599
14	6	0	1.694676	-0.730843	0.178838
15	6	0	4.280747	1.955040	-0.240026
16	6	0	2.954510	2.730198	-0.017702
17	8	0	1.760086	-3.203580	-0.331479
18	8	0	4.344155	-2.794013	-1.553484

19	8	0	-2.958396	-2.411916	0.439127
20	8	0	-3.936924	0.040687	0.702859
21	6	0	-4.518497	1.327847	0.831643
22	1	0	1.919442	-3.351611	0.616345
23	1	0	3.819073	-3.612203	-1.515071
24	1	0	-3.851092	-2.038302	0.540317
25	1	0	2.006761	-1.760220	-1.706210
26	1	0	4.025299	-2.411511	0.453668
27	1	0	5.590139	-0.642803	-0.585293
28	1	0	1.923671	0.662840	-1.441397
29	1	0	0.382332	2.560825	-0.645126
30	1	0	0.336948	2.740711	1.113738
31	1	0	-2.042929	2.038878	0.637537
32	1	0	-0.380193	-2.600828	0.169891
33	1	0	2.088718	-0.826795	1.204415
34	1	0	4.843169	2.355169	-1.091248
35	1	0	4.932058	2.021138	0.638804
36	1	0	3.037834	3.519845	0.737172
37	1	0	2.631109	3.206926	-0.964472
38	1	0	-4.318606	1.946005	-0.053277
39	1	0	-4.146926	1.845516	1.725534
40	1	0	-5.593972	1.167220	0.926599

Cartesian coordinate of SSRR_c1:

Center Number	Atomic Number	Atomic Type	Coordinates (Angstroms) X	Y	Z
1	6	0	2.271739	-1.702581	-0.663269
2	6	0	3.810488	-1.758433	-0.547431
3	6	0	4.507313	-0.416072	-0.561504

4	6	0	3.821816	0.721778	-0.449359
5	6	0	2.319934	0.718428	-0.404269
6	7	0	1.995785	1.888846	0.397325
7	6	0	0.583990	2.211496	0.282295
8	6	0	-0.297269	0.969907	0.387363
9	6	0	-1.677925	1.176284	0.544903
10	6	0	-2.555937	0.102689	0.600449
11	6	0	-2.059609	-1.210818	0.502308
12	6	0	-0.696326	-1.421807	0.355963
13	6	0	0.201456	-0.343241	0.287451
14	6	0	1.711416	-0.549301	0.185266
15	6	0	4.273229	2.155724	-0.229766
16	6	0	2.938767	2.923152	-0.034088
17	8	0	1.753426	-2.964963	-0.258775
18	8	0	4.222362	-2.635807	-1.614001
19	8	0	-2.916725	-2.272418	0.558320
20	8	0	-3.922966	0.175241	0.751981
21	6	0	-4.515686	1.457943	0.865604
22	1	0	2.221494	-3.619060	-0.805031
23	1	0	5.139637	-2.900968	-1.446499
24	1	0	-3.810923	-1.905045	0.667511
25	1	0	2.026034	-1.519597	-1.722088
26	1	0	4.028785	-2.251796	0.415046
27	1	0	5.598239	-0.427987	-0.567138
28	1	0	1.930235	0.838607	-1.442159
29	1	0	0.374759	2.713498	-0.688501
30	1	0	0.311641	2.931200	1.066064
31	1	0	-2.052851	2.192785	0.620756
32	1	0	-0.329967	-2.438147	0.286735
33	1	0	2.111887	-0.661407	1.203890
34	1	0	4.851948	2.557356	-1.069344
35	1	0	4.905028	2.225051	0.663178

36	1	0	3.007659	3.725409	0.708839
37	1	0	2.623017	3.381976	-0.992575
38	1	0	-4.333727	2.062894	-0.032412
39	1	0	-4.138087	1.995688	1.745167
40	1	0	-5.588391	1.288797	0.977182

Cartesian coordinate of SSRR_c2:

Center Number	Atomic Number	Atomic Type	Coordinates (Angstroms) X	Y	Z
1	6	0	2.249146	-1.876977	-0.800775
2	6	0	3.787337	-1.954298	-0.699682
3	6	0	4.474222	-0.607552	-0.690345
4	6	0	3.787200	0.526529	-0.548661
5	6	0	2.286774	0.512765	-0.474486
6	7	0	1.951573	1.690045	0.308909
7	6	0	0.534328	1.977959	0.171369
8	6	0	-0.312776	0.724196	0.380264
9	6	0	-1.686936	0.900943	0.614527
10	6	0	-2.522427	-0.188092	0.822552
11	6	0	-1.983858	-1.488878	0.829265
12	6	0	-0.626390	-1.667961	0.605861
13	6	0	0.223139	-0.577575	0.347029
14	6	0	1.720207	-0.763381	0.132453
15	6	0	4.226778	1.964242	-0.324968
16	6	0	2.884556	2.724338	-0.149170
17	8	0	1.801312	-3.209916	-0.521898
18	8	0	4.311492	-2.744017	-1.764773
19	8	0	-2.788423	-2.564642	1.066953
20	8	0	-3.876953	-0.144374	1.059111

21	6	0	-4.505864	1.126100	1.118526
22	1	0	0.925081	-3.316038	-0.920975
23	1	0	3.839941	-3.592137	-1.703172
24	1	0	-3.685259	-2.217770	1.216594
25	1	0	1.998189	-1.623359	-1.841325
26	1	0	4.006868	-2.456574	0.261049
27	1	0	5.561596	-0.628933	-0.734520
28	1	0	1.874490	0.616482	-1.506241
29	1	0	0.313807	2.402640	-0.833511
30	1	0	0.243851	2.743677	0.902532
31	1	0	-2.090518	1.908893	0.638152
32	1	0	-0.227922	-2.673127	0.676806
33	1	0	2.191561	-0.904466	1.116194
34	1	0	4.816945	2.369954	-1.154258
35	1	0	4.841450	2.039721	0.579502
36	1	0	2.944731	3.546083	0.572684
37	1	0	2.564462	3.153155	-1.119875
38	1	0	-4.406634	1.664290	0.166991
39	1	0	-4.087970	1.739988	1.926833
40	1	0	-5.561543	0.933218	1.317578

Cartesian coordinate of SSRR_c3:

Center Number	Atomic Number	Atomic Type	Coordinates (Angstroms) X	Y	Z
1	6	0	2.207633	-1.709876	-0.441423
2	6	0	3.747328	-1.770088	-0.336347
3	6	0	4.429680	-0.426255	-0.392201
4	6	0	3.739200	0.716215	-0.353395
5	6	0	2.228815	0.738944	-0.305496

6	7	0	1.892739	2.008912	0.367658
7	6	0	0.798693	1.929233	1.337349
8	6	0	-0.259100	0.929375	0.917868
9	6	0	-1.622930	1.232764	1.010931
10	6	0	-2.573998	0.281490	0.654045
11	6	0	-2.165811	-0.984137	0.193501
12	6	0	-0.811276	-1.286037	0.096444
13	6	0	0.149058	-0.335788	0.460426
14	6	0	1.651923	-0.530082	0.363607
15	6	0	4.209268	2.128187	-0.118739
16	6	0	3.126884	2.626945	0.861300
17	8	0	1.675412	-2.949916	0.006273
18	8	0	4.256055	-2.717550	-1.291097
19	8	0	-3.098519	-1.915133	-0.161831
20	8	0	-3.941477	0.444967	0.695175
21	6	0	-4.452777	1.687145	1.148568
22	1	0	2.194519	-3.633297	-0.452779
23	1	0	4.197814	-2.296954	-2.165267
24	1	0	-3.970297	-1.508042	-0.016551
25	1	0	1.945110	-1.552429	-1.504563
26	1	0	3.977403	-2.238771	0.630266
27	1	0	5.518677	-0.434597	-0.362261
28	1	0	1.817779	0.766223	-1.329064
29	1	0	0.351911	2.924912	1.459922
30	1	0	1.189725	1.643727	2.337426
31	1	0	-1.928392	2.214890	1.357759
32	1	0	-0.515298	-2.270831	-0.243853
33	1	0	2.066141	-0.599138	1.382496
34	1	0	4.168330	2.716774	-1.045435
35	1	0	5.223289	2.193303	0.286202
36	1	0	3.375736	2.300096	1.890284
37	1	0	3.034832	3.720112	0.877371

38	1	0	-4.127190	2.512867	0.502424
39	1	0	-4.142747	1.893376	2.181385
40	1	0	-5.540297	1.601873	1.106894

Cartesian coordinate of SSRR_c4:

Center Number	Atomic Number	Atomic Type	Coordinates (Angstroms) X	Y	Z
1	6	0	2.192224	-1.906822	-0.902527
2	6	0	3.733594	-1.964169	-0.826913
3	6	0	4.403455	-0.614871	-0.883378
4	6	0	3.710777	0.522373	-0.806816
5	6	0	2.202259	0.542173	-0.722181
6	7	0	1.879191	1.795322	-0.011506
7	6	0	0.818229	1.690882	0.990569
8	6	0	-0.250013	0.695027	0.587131
9	6	0	-1.610963	0.989271	0.735090
10	6	0	-2.570939	0.042143	0.390960
11	6	0	-2.174912	-1.209885	-0.115369
12	6	0	-0.823329	-1.502401	-0.269185
13	6	0	0.146322	-0.556999	0.085007
14	6	0	1.645998	-0.741536	-0.064147
15	6	0	4.180896	1.933364	-0.564387
16	6	0	3.125214	2.413369	0.453397
17	8	0	1.679359	-3.205701	-0.575101
18	8	0	4.250338	-2.806999	-1.850815
19	8	0	-3.115234	-2.136655	-0.459008
20	8	0	-3.935893	0.197605	0.485567
21	6	0	-4.437096	1.436032	0.961740
22	1	0	1.777317	-3.328067	0.384001

23	1	0	3.736803	-3.630990	-1.788983
24	1	0	-3.983746	-1.733815	-0.284616
25	1	0	1.911127	-1.761961	-1.951770
26	1	0	3.980886	-2.407798	0.160489
27	1	0	5.492012	-0.619554	-0.898225
28	1	0	1.763767	0.588678	-1.733001
29	1	0	0.371092	2.681350	1.148992
30	1	0	1.241974	1.387295	1.972481
31	1	0	-1.907724	1.961967	1.114420
32	1	0	-0.539861	-2.472848	-0.658896
33	1	0	2.093394	-0.814244	0.943475
34	1	0	4.112519	2.535682	-1.480469
35	1	0	5.205311	1.997675	-0.186425
36	1	0	3.404902	2.073473	1.470333
37	1	0	3.027356	3.505660	0.488032
38	1	0	-4.132167	2.266225	0.311512
39	1	0	-4.097892	1.636140	1.986485
40	1	0	-5.525023	1.347680	0.951146

Cartesian coordinate of SSRR_c7:

Center Number	Atomic Number	Atomic Type	Coordinates (Angstroms) X	Y	Z
1	6	0	2.242914	-2.100158	-0.143537
2	6	0	3.801327	-2.152194	-0.083904
3	6	0	4.495857	-0.807294	-0.039498
4	6	0	3.824162	0.342847	0.016066
5	6	0	2.323011	0.359375	0.015146
6	7	0	1.996224	1.555329	0.777863
7	6	0	0.592486	1.894990	0.633466

8	6	0	-0.305878	0.667859	0.754703
9	6	0	-1.686796	0.896574	0.874296
10	6	0	-2.581936	-0.162470	0.933815
11	6	0	-2.102124	-1.484219	0.871975
12	6	0	-0.738823	-1.716600	0.761549
13	6	0	0.178521	-0.653671	0.698846
14	6	0	1.689394	-0.881046	0.632307
15	6	0	4.292020	1.774577	0.214532
16	6	0	2.964886	2.566277	0.349925
17	8	0	1.714172	-3.355925	0.273150
18	8	0	4.333809	-2.982072	-1.118659
19	8	0	-2.973965	-2.532898	0.926499
20	8	0	-3.949992	-0.067808	1.052593
21	6	0	-4.528228	1.225370	1.117870
22	1	0	1.984099	-3.496939	1.195619
23	1	0	4.237394	-2.491768	-1.951303
24	1	0	-3.866525	-2.152881	1.000706
25	1	0	1.942222	-2.015639	-1.195617
26	1	0	4.063228	-2.697293	0.836009
27	1	0	5.584403	-0.843739	-0.013735
28	1	0	1.957220	0.452372	-1.034673
29	1	0	0.403239	2.376127	-0.351871
30	1	0	0.319165	2.637187	1.395671
31	1	0	-2.048416	1.919816	0.916388
32	1	0	-0.389963	-2.739958	0.704935
33	1	0	2.073537	-0.945448	1.663904
34	1	0	4.902977	2.145642	-0.616173
35	1	0	4.897678	1.855960	1.124261
36	1	0	3.022773	3.384738	1.075850
37	1	0	2.684347	3.006780	-0.627941
38	1	0	-4.316838	1.803350	0.208741
39	1	0	-4.164405	1.781262	1.991841

40	1	0	-5.605312	1.072740	1.208088

Cartesian coordinate of SSRR_c5:

Center Number	Atomic Number	Atomic Type	Coordinates (Angstroms) X	Y	Z
1	6	0	2.218632	-1.708613	-0.805079
2	6	0	3.754876	-1.760333	-0.715845
3	6	0	4.431823	-0.411497	-0.769269
4	6	0	3.739887	0.727746	-0.715057
5	6	0	2.230649	0.742251	-0.661276
6	7	0	1.890422	2.007360	0.020175
7	6	0	0.800809	1.917554	0.993310
8	6	0	-0.252782	0.913521	0.573621
9	6	0	-1.617747	1.209497	0.673338
10	6	0	-2.565390	0.255042	0.316011
11	6	0	-2.152390	-1.006258	-0.151572
12	6	0	-0.796733	-1.300635	-0.255345
13	6	0	0.160564	-0.347367	0.108784
14	6	0	1.664577	-0.532520	0.005683
15	6	0	4.204280	2.140496	-0.473618
16	6	0	3.122908	2.629019	0.512534
17	8	0	1.695662	-2.950590	-0.343940
18	8	0	4.154271	-2.661005	-1.766402
19	8	0	-3.081772	-1.940948	-0.506811
20	8	0	-3.933769	0.411396	0.363409
21	6	0	-4.449100	1.651470	0.817493
22	1	0	2.154318	-3.630080	-0.867003
23	1	0	5.083574	-2.896848	-1.622741
24	1	0	-3.954915	-1.537741	-0.359106

25	1	0	1.955256	-1.560692	-1.865484
26	1	0	3.994621	-2.226253	0.255497
27	1	0	5.522531	-0.405550	-0.745146
28	1	0	1.816124	0.771120	-1.682844
29	1	0	0.348548	2.910058	1.122161
30	1	0	1.197009	1.629993	1.990973
31	1	0	-1.926666	2.188649	1.025497
32	1	0	-0.496793	-2.282232	-0.600883
33	1	0	2.082052	-0.601347	1.023291
34	1	0	4.157894	2.733477	-1.397167
35	1	0	5.219332	2.209024	-0.071352
36	1	0	3.376892	2.298225	1.539275
37	1	0	3.025426	3.721697	0.534879
38	1	0	-4.128219	2.478413	0.170531
39	1	0	-4.137785	1.859401	1.849627
40	1	0	-5.536396	1.561852	0.778260

5.2.6. SSRS configuration

Conformer	Population (%)
SSRS_c0.log	60.498
SSRS_c1.log	39.502

Cartesian coordinate of SSRS_c0:

Center Number	Atomic Number	Atomic Type	Coordinates (Angstroms) X	Y	Z
1	6	0	2.499059	-1.788317	0.501788
2	6	0	4.042603	-1.861995	0.451493
3	6	0	4.632041	-0.467383	0.443941
4	6	0	3.961670	0.557654	-0.084452

5	6	0	2.593430	0.421852	-0.708739
6	7	0	1.850846	1.357431	0.158121
7	6	0	0.490601	1.589596	-0.301119
8	6	0	-0.236506	0.282791	-0.557150
9	6	0	-1.640868	0.295315	-0.623446
10	6	0	-2.344355	-0.865838	-0.909233
11	6	0	-1.647917	-2.070678	-1.138359
12	6	0	-0.263906	-2.080264	-1.070473
13	6	0	0.459099	-0.912031	-0.776739
14	6	0	1.976702	-0.981458	-0.707407
15	6	0	4.163504	2.039903	0.137237
16	6	0	2.696404	2.563831	0.244972
17	8	0	2.070021	-1.253278	1.742073
18	8	0	4.384198	-2.651682	-0.699185
19	8	0	-2.333777	-3.213570	-1.426513
20	8	0	-3.712000	-0.989407	-1.001083
21	6	0	-4.504349	0.164177	-0.771748
22	1	0	1.988222	-0.287222	1.623317
23	1	0	5.333030	-2.538919	-0.862111
24	1	0	-3.279371	-2.984194	-1.417269
25	1	0	2.117465	-2.814426	0.444335
26	1	0	4.372891	-2.373673	1.367583
27	1	0	5.558652	-0.293861	0.991272
28	1	0	2.620226	0.802757	-1.752460
29	1	0	0.491865	2.202673	-1.228018
30	1	0	-0.042385	2.177677	0.457401
31	1	0	-2.170214	1.226840	-0.446745
32	1	0	0.252311	-3.018849	-1.251712
33	1	0	2.348819	-1.504621	-1.595961
34	1	0	4.673286	2.518239	-0.708414
35	1	0	4.754159	2.243423	1.035009
36	1	0	2.501509	3.097195	1.181449

37	1	0	2.475582	3.257283	-0.585595
38	1	0	-4.351285	0.557271	0.241672
39	1	0	-5.542727	-0.152582	-0.885314
40	1	0	-4.281678	0.952436	-1.502786

Cartesian coordinate of SSRS_c1:

Center Number	Atomic Number	Atomic Type	Coordinates (Angstroms) X	Y	Z
1	6	0	2.488230	-1.671297	0.467282
2	6	0	4.035818	-1.750256	0.395325
3	6	0	4.631655	-0.363924	0.391910
4	6	0	3.949942	0.675153	-0.089503
5	6	0	2.569376	0.561442	-0.686324
6	7	0	1.843346	1.468204	0.224018
7	6	0	0.476499	1.717449	-0.205912
8	6	0	-0.255794	0.420743	-0.499175
9	6	0	-1.660842	0.437337	-0.548081
10	6	0	-2.370479	-0.711493	-0.867008
11	6	0	-1.679496	-1.908260	-1.148465
12	6	0	-0.294844	-1.921836	-1.097963
13	6	0	0.434532	-0.766791	-0.770414
14	6	0	1.952245	-0.839708	-0.717488
15	6	0	4.159114	2.151175	0.163229
16	6	0	2.695263	2.669924	0.336671
17	8	0	2.073582	-1.164761	1.724073
18	8	0	4.478449	-2.467732	-0.766668
19	8	0	-2.370650	-3.039234	-1.469595
20	8	0	-3.739102	-0.829702	-0.946502
21	6	0	-4.526662	0.316867	-0.669004

22	1	0	1.995176	-0.195054	1.627393
23	1	0	4.215907	-3.395359	-0.647972
24	1	0	-3.315658	-2.808631	-1.442500
25	1	0	2.092658	-2.695010	0.395977
26	1	0	4.373821	-2.262938	1.308307
27	1	0	5.585582	-0.215767	0.894740
28	1	0	2.575189	0.974424	-1.717798
29	1	0	0.463812	2.362985	-1.110239
30	1	0	-0.045602	2.277565	0.580801
31	1	0	-2.186160	1.362723	-0.331680
32	1	0	0.216303	-2.854325	-1.320972
33	1	0	2.319283	-1.339245	-1.622393
34	1	0	4.636977	2.652124	-0.687798
35	1	0	4.781043	2.333539	1.044130
36	1	0	2.528891	3.162821	1.300433
37	1	0	2.450281	3.397775	-0.456426
38	1	0	-4.361329	0.674737	0.355434
39	1	0	-5.566795	0.005665	-0.781518
40	1	0	-4.310551	1.129050	-1.375312

5.2.7. SSSR configuration

Conformer	Population (%)
SSSR_c0.log	40.592
SSSR_c2.log	35.402
SSSR_c1.log	22.945
SSSR_c3.log	1.061

Cartesian coordinate of SSSR_c0:

Center Number	Atomic Number	Atomic Type	Coordinates (Angstroms) X	Y	Z

1	6	0	-0.782020	-1.748217	3.287082
2	6	0	0.761840	-1.792615	3.210151
3	6	0	1.393162	-0.423240	3.260514
4	6	0	0.673574	0.687793	3.098823
5	6	0	-0.817738	0.699965	2.802623
6	7	0	-0.964818	1.785787	1.806549
7	6	0	-0.439019	1.394494	0.488550
8	6	0	-0.966932	0.063582	0.006464
9	6	0	-1.012921	-0.184034	-1.377581
10	6	0	-1.457542	-1.404540	-1.864180
11	6	0	-1.878872	-2.405041	-0.963802
12	6	0	-1.837774	-2.157227	0.398996
13	6	0	-1.369385	-0.929592	0.905620
14	6	0	-1.423400	-0.653138	2.402436
15	6	0	1.133292	2.131205	3.010397
16	6	0	-0.112881	2.844987	2.387045
17	8	0	-1.220365	-1.453608	4.616495
18	8	0	1.211394	-2.532517	2.069648
19	8	0	-2.333644	-3.601648	-1.432550
20	8	0	-1.553303	-1.770828	-3.185721
21	6	0	-1.170983	-0.824909	-4.172181
22	1	0	-0.817896	-2.104959	5.211867
23	1	0	0.955470	-2.019426	1.285232
24	1	0	-2.284869	-3.561597	-2.403590
25	1	0	-1.145952	-2.737745	2.978665
26	1	0	1.112616	-2.393097	4.062486
27	1	0	2.475553	-0.391111	3.381533
28	1	0	-1.341272	1.039013	3.706991
29	1	0	0.668129	1.351858	0.481270
30	1	0	-0.717867	2.181501	-0.223292
31	1	0	-0.701294	0.598423	-2.062631
32	1	0	-2.186629	-2.941287	1.063381

33	1	0	-2.483868	-0.639768	2.686350
34	1	0	2.029982	2.234493	2.389321
35	1	0	1.380472	2.544085	3.996240
36	1	0	-0.686778	3.357519	3.167547
37	1	0	0.146904	3.585319	1.624878
38	1	0	-0.114421	-0.546451	-4.067635
39	1	0	-1.791010	0.079100	-4.117723
40	1	0	-1.324078	-1.313128	-5.136215

Cartesian coordinate of SSSR_c2:

Center Number	Atomic Number	Atomic Type	Coordinates (Angstroms) X	Y	Z
1	6	0	-0.753904	-1.642562	3.348459
2	6	0	0.783181	-1.689174	3.230105
3	6	0	1.388833	-0.303656	3.313150
4	6	0	0.656351	0.798779	3.127349
5	6	0	-0.828802	0.793140	2.780763
6	7	0	-0.939284	1.836376	1.738458
7	6	0	-0.377138	1.386051	0.454463
8	6	0	-0.924820	0.050084	0.009455
9	6	0	-0.959702	-0.246984	-1.365390
10	6	0	-1.433328	-1.471782	-1.812190
11	6	0	-1.895921	-2.426191	-0.881461
12	6	0	-1.864231	-2.130049	0.471692
13	6	0	-1.367400	-0.897840	0.938340
14	6	0	-1.419698	-0.580027	2.426018
15	6	0	1.106857	2.243689	3.015896
16	6	0	-0.109980	2.922842	2.301429
17	8	0	-1.132394	-1.409695	4.707962

18	8	0	1.228225	-2.372641	2.056269
19	8	0	-2.379444	-3.624705	-1.312826
20	8	0	-1.522939	-1.884279	-3.120030
21	6	0	-1.097868	-0.988895	-4.135895
22	1	0	-0.579639	-0.682889	5.038652
23	1	0	0.918690	-1.857866	1.292509
24	1	0	-2.314951	-3.623416	-2.283798
25	1	0	-1.138674	-2.636692	3.108201
26	1	0	1.143756	-2.309216	4.058827
27	1	0	2.467574	-0.248641	3.456867
28	1	0	-1.386299	1.179526	3.647463
29	1	0	0.728602	1.321830	0.487362
30	1	0	-0.616326	2.151231	-0.294191
31	1	0	-0.618640	0.500685	-2.074780
32	1	0	-2.241884	-2.878457	1.161360
33	1	0	-2.478266	-0.573622	2.715307
34	1	0	2.034955	2.332342	2.441006
35	1	0	1.297253	2.694350	3.997924
36	1	0	-0.716458	3.476924	3.027081
37	1	0	0.186034	3.621693	1.514073
38	1	0	-0.034924	-0.738505	-4.025542
39	1	0	-1.690956	-0.065443	-4.125567
40	1	0	-1.251992	-1.509240	-5.082752

Cartesian coordinate of SSSR_c1:

Center Number	Atomic Number	Atomic Type	Coordinates (Angstroms) X	Y	Z
1	6	0	-0.788435	-1.756215	3.546889
2	6	0	0.750016	-1.807606	3.463579

3	6	0	1.386460	-0.440293	3.498099
4	6	0	0.672508	0.674307	3.338323
5	6	0	-0.821824	0.693362	3.058991
6	7	0	-0.981570	1.785456	2.072578
7	6	0	-0.472256	1.402137	0.745076
8	6	0	-0.997270	0.070347	0.262550
9	6	0	-1.049454	-0.174097	-1.121773
10	6	0	-1.489971	-1.395899	-1.609079
11	6	0	-1.899924	-2.401619	-0.709099
12	6	0	-1.852047	-2.157239	0.654085
13	6	0	-1.388909	-0.927748	1.160783
14	6	0	-1.433143	-0.656221	2.658564
15	6	0	1.135857	2.116705	3.247360
16	6	0	-0.117355	2.838348	2.648130
17	8	0	-1.101376	-1.514972	4.922135
18	8	0	1.187866	-2.551970	2.321222
19	8	0	-2.350436	-3.599406	-1.178213
20	8	0	-1.591512	-1.759032	-2.930700
21	6	0	-1.213566	-0.810965	-3.917023
22	1	0	-2.066338	-1.551143	5.014622
23	1	0	0.955299	-2.027600	1.537334
24	1	0	-2.303064	-3.558696	-2.149316
25	1	0	-1.154108	-2.746431	3.245235
26	1	0	1.085377	-2.398874	4.322753
27	1	0	2.469535	-0.413380	3.612731
28	1	0	-1.331690	1.029339	3.973058
29	1	0	0.634610	1.365528	0.723270
30	1	0	-0.764733	2.191135	0.040921
31	1	0	-0.746088	0.611721	-1.806685
32	1	0	-2.188207	-2.947453	1.317700
33	1	0	-2.500139	-0.631945	2.932126
34	1	0	2.022134	2.219573	2.611397

35	1	0	1.401380	2.525138	4.230185
36	1	0	-0.678262	3.346702	3.440823
37	1	0	0.132166	3.583706	1.887464
38	1	0	-0.157415	-0.530420	-3.814466
39	1	0	-1.835583	0.091493	-3.860042
40	1	0	-1.367896	-1.298467	-4.881181

Cartesian coordinate of SSSR_c3:

Center Number	Atomic Number	Atomic Type	Coordinates (Angstroms) X	Y	Z
1	6	0	-0.742756	-1.471800	3.358178
2	6	0	0.797071	-1.505884	3.280303
3	6	0	1.386223	-0.118288	3.355910
4	6	0	0.653457	0.967143	3.099103
5	6	0	-0.813737	0.942292	2.685896
6	7	0	-0.865602	1.910658	1.569796
7	6	0	-0.261163	1.352056	0.347121
8	6	0	-0.879323	0.031275	-0.043153
9	6	0	-0.919996	-0.336912	-1.398578
10	6	0	-1.449712	-1.560850	-1.782254
11	6	0	-1.972216	-2.433699	-0.806960
12	6	0	-1.941576	-2.061541	0.528040
13	6	0	-1.377417	-0.838245	0.929147
14	6	0	-1.406958	-0.447201	2.397224
15	6	0	1.107570	2.404885	2.937567
16	6	0	-0.051731	3.029873	2.089201
17	8	0	-1.143154	-1.219320	4.707332
18	8	0	1.284507	-2.080736	2.064605
19	8	0	-2.515846	-3.629856	-1.177446

20	8	0	-1.549207	-2.041594	-3.068650
21	6	0	-1.063928	-1.228059	-4.124180
22	1	0	-0.621477	-0.461855	5.020273
23	1	0	0.949596	-2.990801	2.012699
24	1	0	-2.448521	-3.679024	-2.146993
25	1	0	-1.126401	-2.473864	3.136671
26	1	0	1.138449	-2.102854	4.142290
27	1	0	2.457203	-0.048392	3.540782
28	1	0	-1.402302	1.394254	3.499500
29	1	0	0.832575	1.214510	0.450962
30	1	0	-0.409883	2.085014	-0.455093
31	1	0	-0.533046	0.350986	-2.144066
32	1	0	-2.379774	-2.742416	1.252634
33	1	0	-2.461358	-0.426917	2.701284
34	1	0	2.077922	2.463556	2.433597
35	1	0	1.216452	2.916540	3.902227
36	1	0	-0.691679	3.654770	2.723081
37	1	0	0.306375	3.652043	1.264272
38	1	0	0.009415	-1.027615	-4.011074
39	1	0	-1.606149	-0.274841	-4.174383
40	1	0	-1.233257	-1.790573	-5.044220

5.2.8. SSSS configuration

Conformer	Population (%)
SSSS_c1.log	63.651
SSSS_c3.log	20.554
SSSS_c6.log	12.714
SSSS_c12.log	3.081

Cartesian coordinate of SSSS_c1:

Center Atomic Atomic Coordinates (Angstroms)

Number	Number	Type	X	Y	Z
1	6	0	1.781798	2.089820	0.717547
2	6	0	3.232259	2.129691	1.279264
3	6	0	4.152865	1.032351	0.783110
4	6	0	3.725207	0.040986	-0.005669
5	6	0	2.314085	0.013123	-0.503108
6	7	0	2.032260	-1.402379	-0.704397
7	6	0	0.806176	-1.567677	-1.469228
8	6	0	-0.314627	-0.673100	-0.940764
9	6	0	-1.623456	-0.926331	-1.386721
10	6	0	-2.686051	-0.127431	-0.977580
11	6	0	-2.453874	0.948157	-0.099303
12	6	0	-1.164148	1.197830	0.350685
13	6	0	-0.081678	0.403206	-0.063616
14	6	0	1.327386	0.640826	0.460864
15	6	0	4.394623	-1.233157	-0.496340
16	6	0	3.263795	-1.951338	-1.282966
17	8	0	1.783429	2.876402	-0.480093
18	8	0	3.209295	2.117798	2.719736
19	8	0	-3.486104	1.739375	0.316774
20	8	0	-4.002577	-0.277471	-1.346961
21	6	0	-4.344797	-1.342878	-2.226610
22	1	0	2.263980	0.561959	-1.468030
23	1	0	1.424486	0.105197	1.418091
24	1	0	1.153799	2.569860	1.478400
25	1	0	3.632284	3.115221	1.018047
26	1	0	5.172097	1.064580	1.165006
27	1	0	0.977503	-1.321892	-2.540265
28	1	0	0.490978	-2.617780	-1.432395
29	1	0	-1.797445	-1.759397	-2.060490
30	1	0	-1.020974	2.019897	1.045450

31	1	0	4.729314	-1.839443	0.352721
32	1	0	5.268839	-1.040314	-1.126975
33	1	0	3.340579	-1.707833	-2.361270
34	1	0	3.293694	-3.040935	-1.182354
35	1	0	0.893290	2.861333	-0.858480
36	1	0	3.159716	1.197751	3.013978
37	1	0	-4.301496	1.409888	-0.091566
38	1	0	-5.422383	-1.272402	-2.378429
39	1	0	-3.831701	-1.238837	-3.190312
40	1	0	-4.100324	-2.315158	-1.782052

Cartesian coordinate of SSSS_c3:

Center Number	Atomic Number	Atomic Type	Coordinates (Angstroms) X	Y	Z
1	6	0	1.325069	0.098402	-2.252681
2	6	0	1.648044	-1.055322	-3.250181
3	6	0	0.567151	-2.101460	-3.414451
4	6	0	-0.593693	-2.042370	-2.758350
5	6	0	-0.885925	-0.910590	-1.824150
6	7	0	-1.803939	-1.482059	-0.847474
7	6	0	-2.407190	-0.430891	-0.045815
8	6	0	-1.361680	0.568092	0.448578
9	6	0	-1.737362	1.466099	1.462565
10	6	0	-0.848118	2.425791	1.935006
11	6	0	0.450474	2.498987	1.397030
12	6	0	0.829393	1.608863	0.400869
13	6	0	-0.062966	0.640795	-0.091041
14	6	0	0.359032	-0.367988	-1.150648
15	6	0	-1.759258	-3.014066	-2.661920

16	6	0	-2.702047	-2.338498	-1.628781
17	8	0	0.792274	1.176080	-3.032313
18	8	0	2.825611	-1.772390	-2.831636
19	8	0	1.338918	3.431508	1.852132
20	8	0	-1.102807	3.353574	2.918511
21	6	0	-2.385290	3.354940	3.535848
22	1	0	-1.378806	-0.087932	-2.386344
23	1	0	0.851377	-1.213817	-0.649483
24	1	0	2.284223	0.405249	-1.811209
25	1	0	1.841680	-0.571579	-4.218790
26	1	0	0.820998	-2.933523	-4.067643
27	1	0	-3.172949	0.121577	-0.634086
28	1	0	-2.927728	-0.876652	0.810854
29	1	0	-2.738542	1.401898	1.877009
30	1	0	1.845606	1.675886	0.024292
31	1	0	-1.412925	-3.988187	-2.299228
32	1	0	-2.260498	-3.178642	-3.621556
33	1	0	-3.470869	-1.737251	-2.154221
34	1	0	-3.220062	-3.055731	-0.984077
35	1	0	0.502550	1.876041	-2.430222
36	1	0	3.602560	-1.227850	-3.016737
37	1	0	0.901899	3.949390	2.545641
38	1	0	-2.362239	4.157304	4.274019
39	1	0	-3.175871	3.554709	2.802401
40	1	0	-2.581996	2.400211	4.038383

Cartesian coordinate of SSSS_c6:

Center Number	Atomic Number	Atomic Type	Coordinates (Angstroms) X	Y	Z

1	6	0	1.464100	-2.468532	-0.593935
2	6	0	0.997635	-3.959344	-0.589037
3	6	0	-0.471462	-4.172835	-0.284858
4	6	0	-1.276873	-3.168973	0.079024
5	6	0	-0.775292	-1.759338	0.140771
6	7	0	-1.587063	-1.145770	1.185087
7	6	0	-1.416206	0.299204	1.175591
8	6	0	0.057383	0.693057	1.092747
9	6	0	0.396853	2.027313	1.378006
10	6	0	1.712876	2.465184	1.279401
11	6	0	2.721792	1.563146	0.892391
12	6	0	2.391713	0.244395	0.615689
13	6	0	1.066337	-0.207144	0.704403
14	6	0	0.702535	-1.665304	0.468565
15	6	0	-2.709738	-3.153436	0.587287
16	6	0	-2.940685	-1.662738	0.958851
17	8	0	1.222489	-1.856527	-1.865612
18	8	0	1.802723	-4.733108	0.321198
19	8	0	4.020861	1.977631	0.792148
20	8	0	2.164518	3.741427	1.532331
21	6	0	1.218534	4.730953	1.918341
22	1	0	-0.954435	-1.265472	-0.837220
23	1	0	0.849239	-2.199963	1.419847
24	1	0	2.539613	-2.484391	-0.377213
25	1	0	1.242088	-4.374376	-1.575670
26	1	0	-0.816237	-5.205505	-0.311856
27	1	0	-1.951838	0.749201	0.311281
28	1	0	-1.864677	0.724050	2.082135
29	1	0	-0.385841	2.717014	1.677928
30	1	0	3.191861	-0.426931	0.322003
31	1	0	-2.810333	-3.800484	1.465627
32	1	0	-3.429735	-3.501613	-0.160951

33	1	0	-3.434182	-1.135949	0.118061
34	1	0	-3.565015	-1.532378	1.848538
35	1	0	1.805878	-2.256711	-2.522962
36	1	0	1.423351	-4.648243	1.206920
37	1	0	4.052782	2.919176	1.020816
38	1	0	1.786181	5.651726	2.058022
39	1	0	0.463268	4.881229	1.137195
40	1	0	0.724603	4.459559	2.859410

Cartesian coordinate of SSSS_c12:

Center Number	Atomic Number	Atomic Type	Coordinates (Angstroms) X	Y	Z
1	6	0	0.001642	-2.398519	0.509219
2	6	0	-1.292828	-3.209799	0.193519
3	6	0	-2.210983	-2.577912	-0.835953
4	6	0	-2.023763	-1.335328	-1.295969
5	6	0	-0.832246	-0.525330	-0.879982
6	7	0	-1.308112	0.848064	-0.926290
7	6	0	-0.195847	1.781680	-0.820168
8	6	0	0.781461	1.382452	0.284173
9	6	0	1.739393	2.331163	0.683231
10	6	0	2.690308	2.020311	1.648865
11	6	0	2.697905	0.740510	2.236155
12	6	0	1.751318	-0.196933	1.848663
13	6	0	0.788057	0.104971	0.874077
14	6	0	-0.300522	-0.886431	0.493625
15	6	0	-2.894372	-0.431214	-2.154720
16	6	0	-2.132660	0.922716	-2.137595
17	8	0	1.072758	-2.778270	-0.358386

18	8	0	-2.039288	-3.467093	1.398660
19	8	0	3.626919	0.419698	3.184883
20	8	0	3.672650	2.861804	2.119472
21	6	0	3.755387	4.176245	1.581927
22	1	0	-0.018362	-0.658833	-1.633561
23	1	0	-1.137729	-0.739479	1.193881
24	1	0	0.323276	-2.721206	1.502063
25	1	0	-0.961381	-4.200072	-0.136889
26	1	0	-3.088052	-3.161648	-1.111616
27	1	0	0.359341	1.831790	-1.782362
28	1	0	-0.586120	2.788648	-0.628548
29	1	0	1.728563	3.315680	0.226218
30	1	0	1.785370	-1.175326	2.315589
31	1	0	-3.891535	-0.331231	-1.712566
32	1	0	-3.025030	-0.809041	-3.174341
33	1	0	-1.501054	1.013937	-3.043526
34	1	0	-2.797991	1.791101	-2.105339
35	1	0	0.884199	-2.487126	-1.260243
36	1	0	-2.591052	-2.695138	1.586419
37	1	0	4.197748	1.191573	3.319630
38	1	0	4.593351	4.656248	2.088711
39	1	0	3.946997	4.149871	0.502219
40	1	0	2.836385	4.741864	1.778555

----

6. VCD spectrum of compounds **2** and **3**

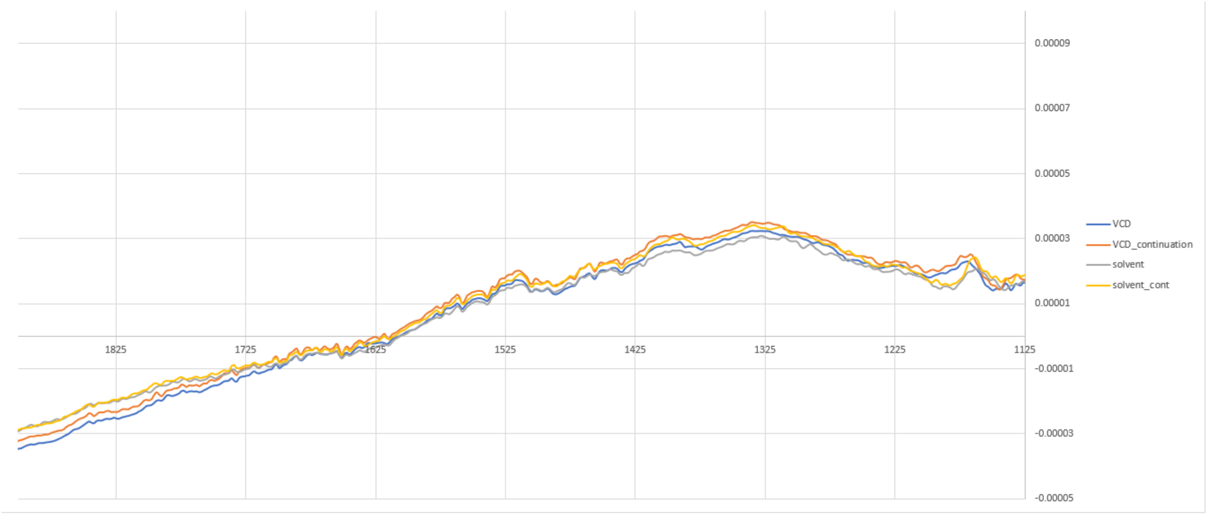

Figure S23. VCD spectrum of compounds **2** and **3** in $(CD_3)_2SO$ without any observed signals

6. Anti-SARS-CoV-2 activity and cytotoxicity

Compound		IC_{50} (μM)		CC_{50} (μM)	SI	av. IC_{50} (μM)	SD	av. CC_{50} (μM)	SD	SI
**1**	=	54.035	>	100	>2	45.80	11.65	>100,00		>2
	=	37.559	>	100	>3					
**2 and 3**	>	100	>	100	X1	>100,00		>100,00		X1
	>	100	>	100	X1					
**4 and 5**	=	44.608	>	100	>2	44.95	0.48	>100,00		>2
	=	45.287	>	100	>2					
**6**	>	100	=	1.2768	<1	>100,00		1.21	0.10	<1
	>	100	=	1.1424	<1					
**7**	=	69.265	>	100	>1	54.24	21.24	>100,00		>2
	=	39.223	>	100	>3					
**8**	=	37.104	>	100	>3	39.63	3.58	>100,00		>3
	=	42.161	>	100	>2					
**9**	>	100	>	100	X1	>100,00		>100,00		X1
	>	100	>	100	X1					
**10**	>	100	=	0.14	<1	>100,00		0.13	0.01	<1
	>	100	=	0.1223	<1					
**11**	=	99.446	>	100	>1	77.17	31.51	>100,00		>1
	=	54.887	>	100	>2					
